# Mapping Functionally Relevant Tractable Lysines of Challenging Protein Targets by Covalent Fragment Screening

**DOI:** 10.1002/cbic.70421

**Published:** 2026-06-11

**Authors:** Noémi Csorba, Péter Ábrányi‐Balogh, Zoltán Orgován, Tibor Viktor Szalai, György G. Ferenczy, Levente Kollár, Nikolett Péczka, Tímea Imre, József Simon, Pál Szabó, Gitta Schlosser, Martina Hrast, Stanislav Gobec, Varbina Ivanova, Carles Galdeano, Aleksandra Kopranovic, Marc Neumann, Franz‐Josef Meyer‐Almes, György Miklós Keserű

**Affiliations:** ^1^ Medicinal Chemistry Research Group HUN‐REN Research Centre for Natural Sciences Budapest Hungary; ^2^ National Laboratory for Drug Research and Development HUN‐REN Research Centre for Natural Sciences Budapest Hungary; ^3^ Department of Organic Chemistry and Technology Faculty of Chemical Technology and Biotechnology Budapest University of Technology and Economics Budapest Hungary; ^4^ Department of Inorganic and Analytical Chemistry Faculty of Chemical Technology and Biotechnology Budapest University of Technology and Economics Budapest Hungary; ^5^ MS Metabolomics Research Group HUN‐REN Research Centre for Natural Sciences Budapest Hungary; ^6^ MTA‐ELTE Lendület (Momentum) Ion Mobility Mass Spectrometry Research Group Institute of Chemistry ELTE Eötvös Loránd University Budapest Hungary; ^7^ Department of Pharmaceutical Chemistry Faculty of Pharmacy University of Ljubljana Ljubljana Slovenia; ^8^ Departament de Farmàcia i Tecnologia Farmacéutica i Fisicoquímica Facultat de Farmàcia i Ciències de l’Alimentació Universitat de Barcelona Barcelona Spain; ^9^ Institut de Química Teòrica i Computacional (IQTC) Universitat de Barcelona Barcelona Spain; ^10^ Institut de Biomedicina de la Universitat de Barcelona (IBUB) Universitat de Barcelona Barcelona Spain; ^11^ Department of Chemical Engineering and Biotechnology University of Applied Sciences Darmstadt Darmstadt Germany

**Keywords:** allosteric sites, covalent fragment, electrophilic warhead, lysine labelling, protein–protein interaction

## Abstract

Covalent fragment screening has become an established strategy for identifying targetable amino acid residues or viable chemical starting points against challenging protein targets. In recent years, lysine has received growing interest as a nucleophilic residue suitable for covalent labelling. Herein, we present a lysine‐targeting covalent fragment library covering a wide range of warheads. The reactivity and stability of the library members have been systematically characterised, and the library has been subsequently screened against a set of therapeutically relevant and challenging protein targets. We have discovered suitable warheads against the targets and characterised binding sites via enzymatic digestion and modelling. These findings highlight the potential of lysine‐targeting covalent chemistry to expand binding site discovery and to support warhead optimisation for diverse protein targets in both medicinal chemistry and chemical biology applications.

## Graphical abstract

Screening a lysine‐targeting covalent fragment library against challenging protein targets revealed suitable warheads and characterized binding sites for the development of targeted covalent ligands.

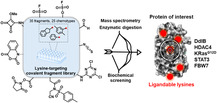

## 1 Introduction

Driven by the clinical success of targeted covalent inhibitors (TCIs) with enhanced potency, selectivity, and prolonged duration of action, covalent drug discovery has gained increasing attention over the past decade [1, 2, 3]. In addition to the ‘ligand‐first’ strategy that typically equips a known ligand of the target of interest with a suitable electrophilic warhead, the ‘electrophile‐first’ approach operates with covalent fragments and develops them into a covalent ligand [1, 3, 4]. Covalent fragments are built from two main components: the scaffold that carries pharmacophore elements and forms non‐covalent interactions at the binding site, and the electrophilic warhead that is able to covalently anchor the ligand to the protein, forming reversible or irreversible covalent bond [5]. Covalent fragments combine the advantages of covalent mechanism of action and fragment‐based drug discovery, effectively mapping the available chemical space for ligand discovery and identifying suitable binding sites on proteins [6]. Covalent fragment approaches have been used successfully against protein–protein interfaces and proteins previously considered ‘undruggable’ with shallow binding sites [7]. Rationally designed covalent inhibitors initially relied on targeting highly nucleophilic cysteine residues, taking advantage of their relatively low pK_a_ and predictable reactivity profiles. However, the number of proteins that contain suitably positioned, functionally relevant, non‐essential cysteine residues is limited, which in turn constrains the accessible chemical space for covalent ligand discovery [4, 8]. Consequently, to overcome this limitation, there is growing interest in extending the toolbox of electrophiles to target alternative amino acid side chains, such as lysine, tyrosine, serine, threonine, histidine, methionine, aspartate, and glutamate [4, 9]. In line with these efforts, the development of amino acid‐specific electrophiles would further enhance target specificity [8]. In the last 5 years, lysine targeting has attracted increasing attention [10]. Although lysine is more abundant than cysteine (5.7% vs. 2.3%) across the proteome [11], only around 3000 of the more than 14,000 mapped lysines have been considered potentially druggable [12]. Importantly, tractable lysine residues are available at challenging protein–protein and protein–RNA interfaces [12]. Lysine‐reactive fragments, therefore, became valuable tools for identifying both active site and allosteric ligands and uncovering functionally important secondary binding sites. Our systematic investigation began with the compilation and detailed characterisation of our lysine targeting electrophilic fragment library, including experimental reactivity parameters and aqueous stability, which together established the reactivity range covered. We have subsequently evaluated the library across a panel of challenging proteins with diverse functional and structural features, including a conventional enzyme, *D*‐alanine:*D*‐alanine ligase B (DdlB), a transcription factor, Signal Transducer and Activator of Transcription 3 (STAT3), an epigenetic target, histone deacetylase 4 (HDAC4), a target involved in protein–protein interactions (PPIs), Kirsten rat sarcoma viral oncogene homologue G12D mutant (KRAS^G12D^), and an E3 ligase, F‐box and WD repeat domain‐containing 7 (FBW7). Collectively, these examples provide a comprehensive view of lysine‐reactive fragment behaviour, revealing how electrophilic reactivity and the local protein microenvironment shape covalent engagement. These insights, in turn, define guiding principles for the identification of tractable and functionally relevant lysines and the rational mapping of binding pockets around those. In addition, we provide insights for the selection of optimal warheads tailored to the given protein target.

## 2 Results and Discussion

### 2.1 Library Design and Characterisation

**FIGURE 1:**
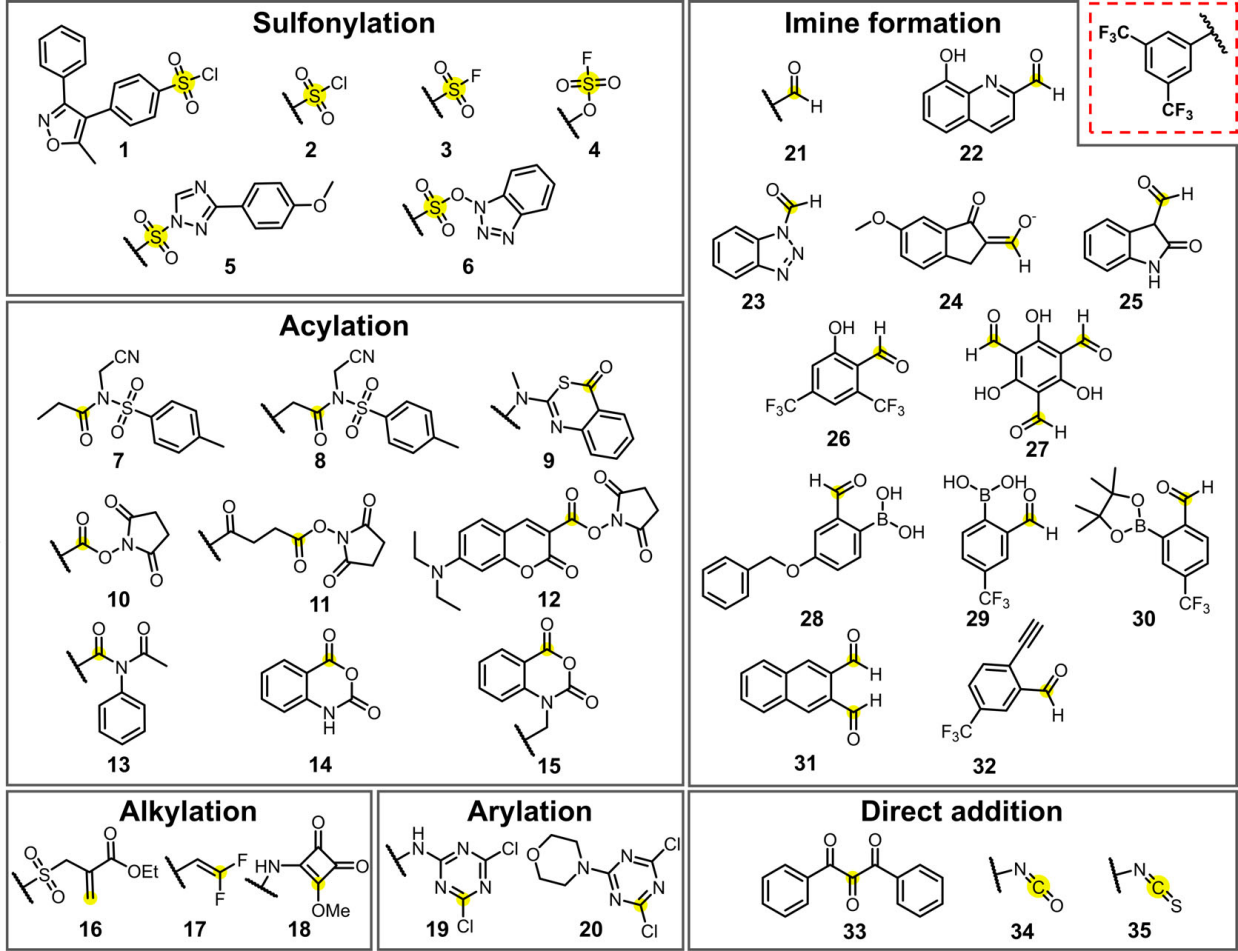
Covalent fragment probes (**1–35**) categorised based on the reaction type of their warheads with the *ε*‐amino group of lysine. They can react either through sulfonylation, acylation, alkylation, arylation, imine formation, or direct addition. The presumed electrophilic centres are highlighted in yellow.

We compiled a 35‐member fragment library (Figure 1, Table S1) equipped with a diverse set of electrophilic warheads for targeting lysine residues [13]. Drawing inspiration from systematic proteome‐wide studies [12], TCI developments and comprehensive warhead reviews [4, 11, 14], the library is composed of 25 distinct aminophilic functional groups, providing broad coverage of labelling chemistries and warheads with diverse reactivity in most cases preferably to the direction of Lys, and in some cases with varying degrees of selectivity for Lys over other residues known from literature precedents (Figure S1) [11, 12]. Since covalent fragment binding is mostly reactivity driven [15], our concept focused on the warheads while keeping non‐covalent interactions similar. Therefore, our probe design kept the non‐covalent moiety less diverse, fragment‐sized, and typically apolar to minimise the contribution of non‐covalent interactions to probe binding and to facilitate the direct comparison of fragment reactivities [2, 16, 17, 18]. Kinetic profiling of the fragment library against *N*‐α‐acetyl‐lysine revealed that the fragments cover a broad reactivity range, including rapid modifications (t_1/2_ < 5 min), intermediate reaction rates (t_1/2_ < 20–90 min), and very slow reactions (t_1/2_ > 24 h). A stability assay at pH 7.4 confirmed that most moderate or weakly reactive fragments retained >80% stability, while several highly reactive fragments also remained sufficiently stable in aqueous conditions, indicating that the library is well‐suited to target lysines in diverse protein environments (Table S1).

### 2.2 Mapping Tractable Lysines at Protein Binding Sites

**FIGURE 2:**
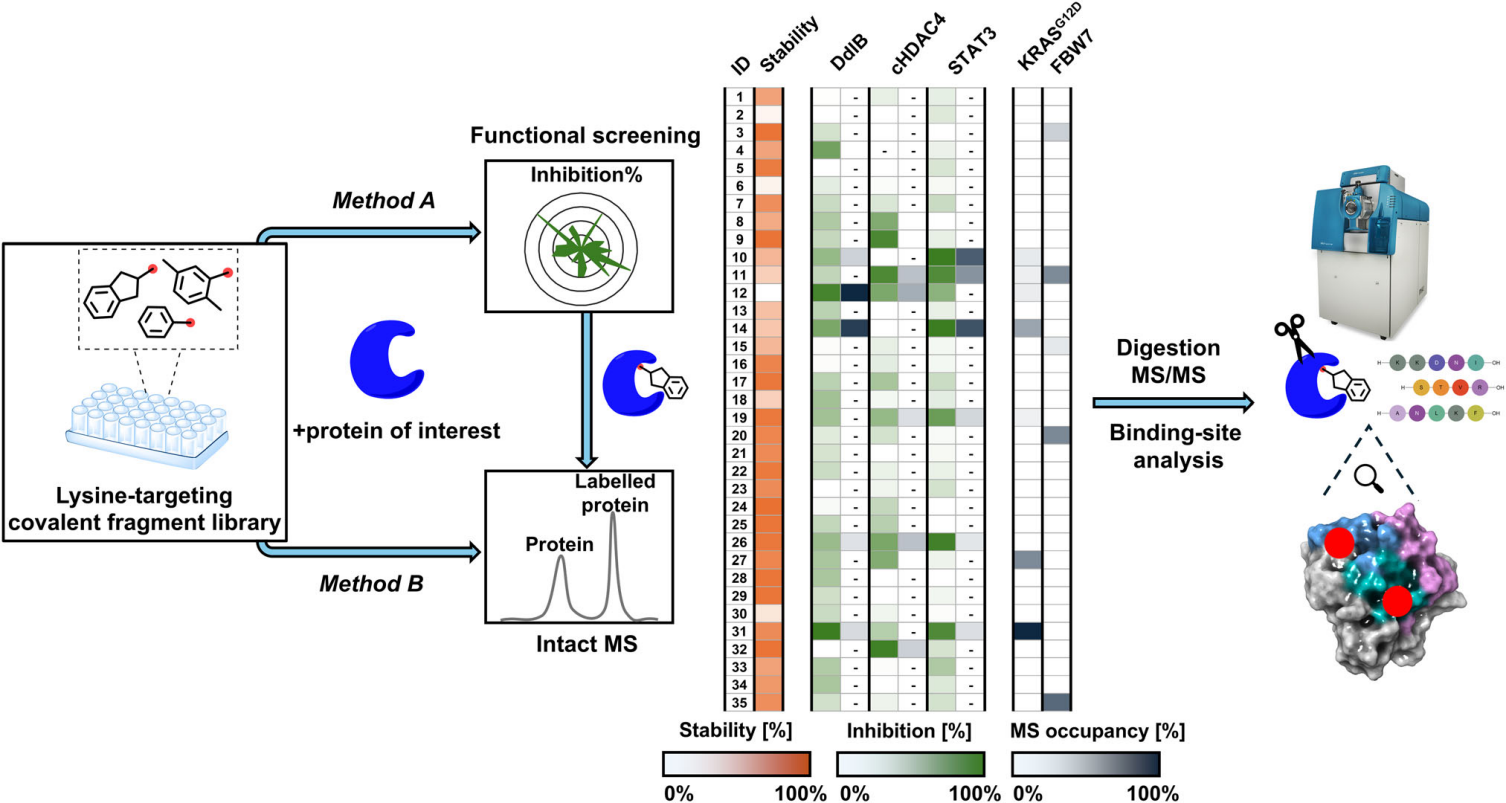
Schematic representation of the applied workflow with the single‐concentration screening results shown on a heat map. The compounds were pre‐incubated with the protein (30 min for DdlB, and 60 min in case of cHDAC4 and STAT3). Intact MS samples were incubated for 1 h and subsequently measured. The heatmap shows functionally relevant hits along with their intact MS occupancy from Method A and Method B, including also the compounds’ stability after 1 h in physiological buffer. (‘‐’ = not determined).

Although surrogate models provide valuable preliminary insights into warhead reactivity and specificity, the outcomes may not fully capture their proteome‐wise behaviour in complex biological systems. Therefore, we aimed to investigate protein‐specific reactivity patterns and map targetable lysine residues across therapeutically relevant proteins. Demonstrating the scope of this binding site mapping protocol, we took five representatives of different protein classes that include a traditional enzyme (DdlB), a transcription factor (STAT3), an epigenetic regulator (HDAC4), a PPI target (KRAS^G12D^), and an E3 ligase (FBW7). We screened the library following two strategies (Figure 2, Table S2). In both strategies, we applied 30 min^−1^ h preincubation of the fragments (as indicated in the specific cases) with the protein prior to the measurements, considering the time‐dependency of the covalent modification. In the case of three targets (DdlB, STAT3, and HDAC4), we performed single concentration biochemical screening to identify functionally relevant covalent fragment hits, and their covalent labelling was subsequently confirmed by intact mass spectrometry (MS) measurements (Method A). Validated covalent ligands were then digested to identify the labelled lysine residues. In the case of KRAS^G12D^ and FBW7, we first screened the fragments by MS and then determined the binding sites by peptide mapping (Method B). Finally, the binding mode of all validated covalent hits was further analysed by computational modelling, including SiteMap [19, 20] and FTMap [21, 22] analysis to gain a deeper understanding of the labelled residue and the surrounding environment.

### 2.3 Profiling an Antibacterial Target: DdlB

**FIGURE 3:**
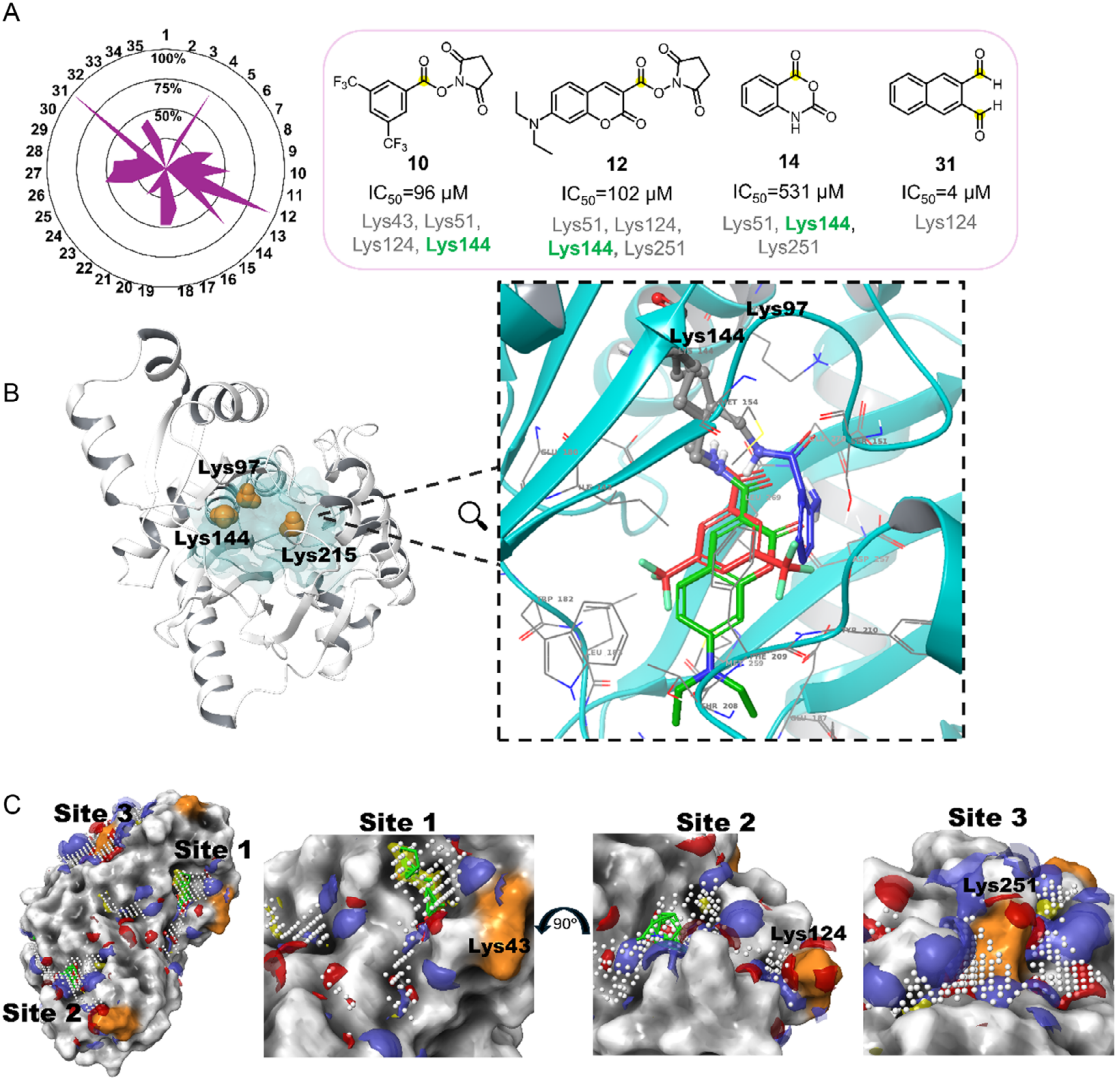
(A) Fragment reactivity pattern against DdlB based on limit‐dose screening at 500 µM after 30 min pre‐incubation at 37 °C, data is represented as mean from duplicates. Active hits are displayed for which covalent labelling and digestion experiments were successful. The corresponding IC_50_ values and labelled residues are indicated (grey: surface lysine; green: active site hit). (B) Left: Structure of *E.* coli DdlB (PDB ID: 4C5A [30]). Protein is shown with ribbons, and the buried binding site with a transparent surface. The ammonium heads of the binding site lysine side chains are shown in red. Right: active site of *E*. coli DdlB. Lysines are shown with ball‐and‐stick and ligands with thick tube representation. Putative complex structures of **10** (red), **12** (green) and **14** (blue) labelling Lys144 of DdlB. The closest residues and the ribbon representation of DdlB are shown. (C) Surface of DdlB with sites identified by SiteMap and FTMap. Labelled lysines are shown in orange, hydrogen‐bond donor regions are shown in blue, hydrogen‐bond acceptor regions are shown in red, and the hydrophobic regions are shown in yellow.

Ddl is an ATP‐dependent enzyme responsible for generating a dipeptide from two *D*‐alanine molecules. This is a key component of the peptidoglycan precursor and thus plays an indispensable role in bacterial cell wall biosynthesis, which is essential for pathogen survival [23, 24, 25]. Consequently, Ddl represents a central target for antimicrobial development. This role has been clinically validated by cycloserine, a second‐line drug used against tuberculosis [26, 27]. In *Escherichia coli*, two isoforms of Ddl, DdlA and DdlB, have been identified [28]. Both exhibit comparable catalytic efficiencies, substrate specificities, and sensitivities to known inhibitors [23, 29]. Our efforts have focused on DdlB, as it is the most characterised isozyme, with available crystal structures that facilitate structure‐based inhibitor design [30, 31]. The active site of *E. coli* DdlB (Figure 3B) contains three lysine residues (Lys97, Lys144, Lys215), each in contact with ATP phosphates and the attachment of a covalent ligand to any of these lysines is assumed to affect ATP binding and enzyme activity [30]. Over the last decades, numerous Ddl inhibitors have been identified; however, only thiocarbamides and chloroalkanes have shown low‐micromolar activity. Most of the other chemotypes exhibited IC_50_s in the 70–500 µM range [25], which highlights the importance of further efforts in Ddl inhibitor discovery. Hence, a spectrophotometric DdlB assay was set up to evaluate inhibition of the covalent fragments against DdlB ligase [31]. First, we performed limit‐dose screening at 500 µM compound concentration, compounds showing >50% inhibition were selected for further evaluation. Among the library members, five hits were identified with acceptable micromolar activity: two *N*‐hydroxysuccinimide esters (**10**, IC_50_ = 96 µM and **12**, IC_50_ = 102 µM), isatoic anhydride (**14**, IC_50_ = 531 µM), a salicylaldehyde derivative (**26**, IC_50_ = 308 µM), and 2,3‐naphthalene‐dicarboxaldehyde (**31**, IC_50_ = 4 µM) (Figure 3A). Their covalent labelling was confirmed by intact MS, followed by tryptic digestion. For the most reactive acylating fragments (**10**, **12**) four lysines were identified by digestion, followed by fragments **14** and **31** in line with their expected warhead reactivity. In the case of salicylaldehyde **26**, despite the multiple labelling observed by intact MS, no labelled residue was identified upon digestion, which could be explained by its reversible binding [32]. Notably, **10**, **12** and **14** labelled Lys144 at the active site (Figure 3B) together with other surface lysines (Lys43, Lys51, Lys124, Lys251). Interestingly, the most potent inhibitor, compound **31**, labelled exclusively Lys124, a surface‐exposed lysine that was identified by SiteMap and FTMap, and may be involved in a putative allosteric mechanism. SiteMap identified a binding pocket close to Lys43 (Figure 3C); however, without direct contact between the Lys43 sidechain atoms and the pocket. A more extended dominantly polar, although shallow pocket delimited by Lys251 was identified both by SiteMap and FTMap. Notably, the active site was also found (Figure S2), where the role of lysines is well established [30]. To the best of our knowledge, this is the first study to identify these residues on DdlB with covalent fragments as possible anchoring points for covalent inhibitor development.

### 2.4 Profiling a Transcription Factor: STAT3

**FIGURE 4:**
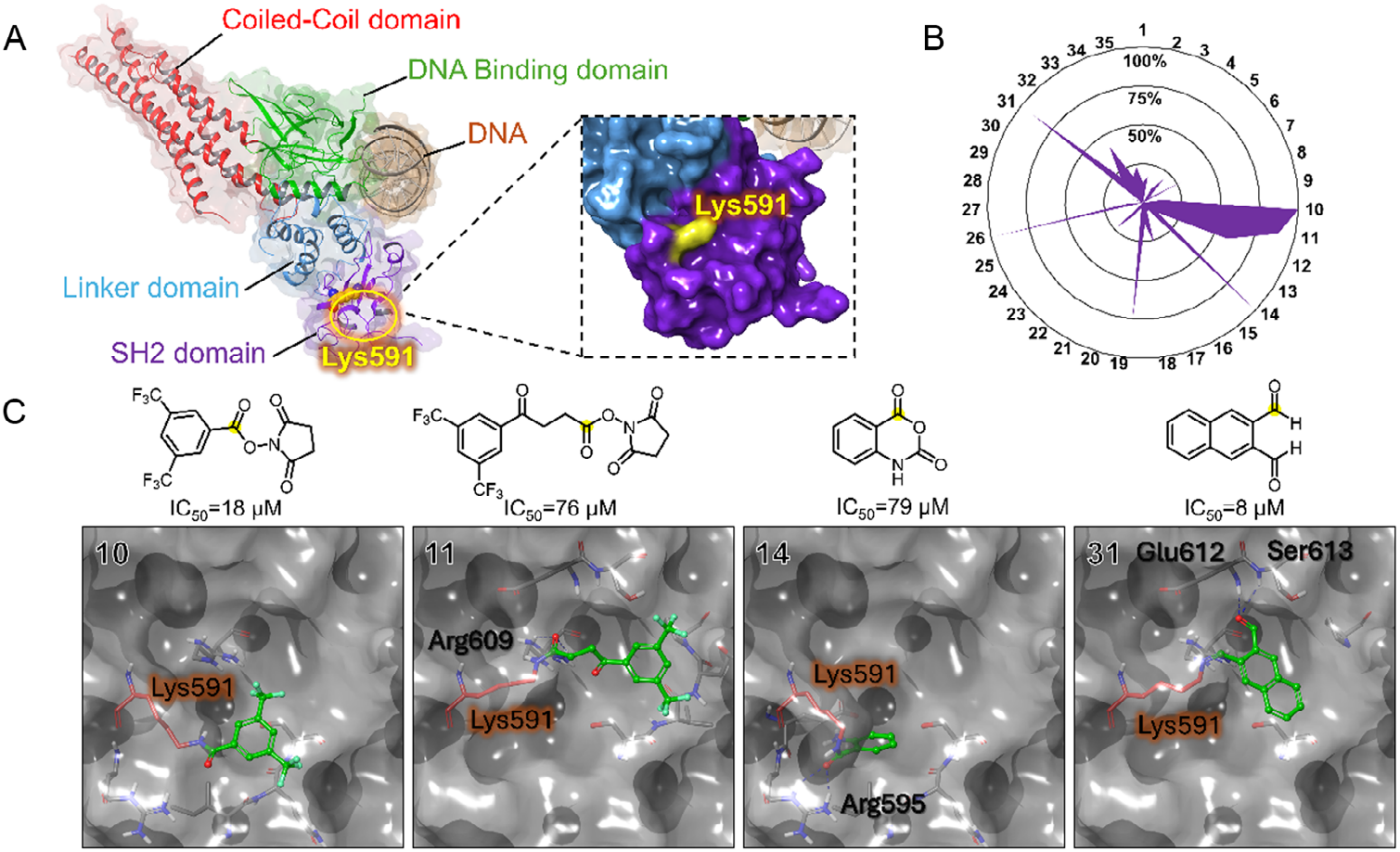
(A) Structural model of STAT3 crystal structure containing DNA (PDB ID: 6QHD [40]) with the individual domains indicated (red = coiled‐coil, green = DNA binding, blue = linker, purple = SH2). Right inset: close‐up of the SH2 domain's phosphopeptide binding interface with the targeted lysine residue (Lys591) highlighted by yellow. (B) Fragment reactivity pattern against STAT3 identified by limit‐dose screening at 500 µM after 60 min pre‐incubation at 37 °C, data is represented as mean from triplicates. (C) Fragment hits with IC_50_ values below 100 µM (FP), along with their best‐scoring predicted binding poses generated by covalent docking (PDB ID: 6QHD). Targeted Lys591 residue is coloured red, and interacting residues are labelled. Interactions are shown as dashed lines, with dark blue colour corresponding to hydrogen bonds.

The STAT protein family plays an essential role in the regulation of DNA transcription in the nucleus by mediating intercellular signalling [33, 34]. Among them, STAT3 is a cytoplasmic transcription factor that has been extensively linked to cancer progression, making it an attractive and validated therapeutic target [35]. Its Src homology‐2 (SH2) domain plays a critical role in STAT3 dimerisation, which is a necessary step for nuclear translocation and oncogenic signalling [36]. However, its shallow binding surface [37] renders small‐molecule inhibition challenging. Therefore, covalent fragment screening could be considered as an effective strategy to identify starting points for novel STAT3 inhibitors. Building on these insights, we aimed to assess lysine reactivity at the SH2 domain and thereby potentially disrupt STAT3 dimerisation and downstream oncogenic signalling. To this end, we screened our fragment library using a modified fluorescence polarisation (FP) assay [38], which enables reliable detection of ligands that interact with the phospho‐Y‐705‐binding site of the SH2 domain by displacing a fluorescein‐labelled phosphotyrosine peptide (GpYLPQTV) from STAT3, inducing inhibition of STAT3 dimerisation [39]. This site is known to accommodate drug‐sized molecules such as BP‐1‐102 and SD‐36, and contains a lysine residue (Lys591) that can presumably be targeted by electrophiles (Figure 4A) [41, 42]. First, we conducted biological screening at 500 and 50 µM. Seven hits showed >50% inhibition at 500 µM (Figure 4B) while six compounds remained active at 50 µM. Next, IC_50_ values were determined for the six hits: naphthalene‐dicarboxaldehyde **31** was the most active (IC_50_ = 8 µM), followed by NHS ester **10** (IC_50_ = 18 µM). IC_50_s of NHS ester **11** and isatoic anhydride **14** were in the range of 70–80 µM, while dichlorotriazine **19** and salicyladehyde **26** showed weaker effect (IC_50_ > 100 µM). Covalent labelling was then proven by intact MS measurements for all the hits, and we further investigated the potential binding modes by covalent docking (Figure 4C, Figure S2), which suggested that covalent targeting of Lys591 can be an efficient strategy to inhibit STAT3.

### 2.5 Profiling an Epigenetic Regulator: HDAC4

**FIGURE 5:**
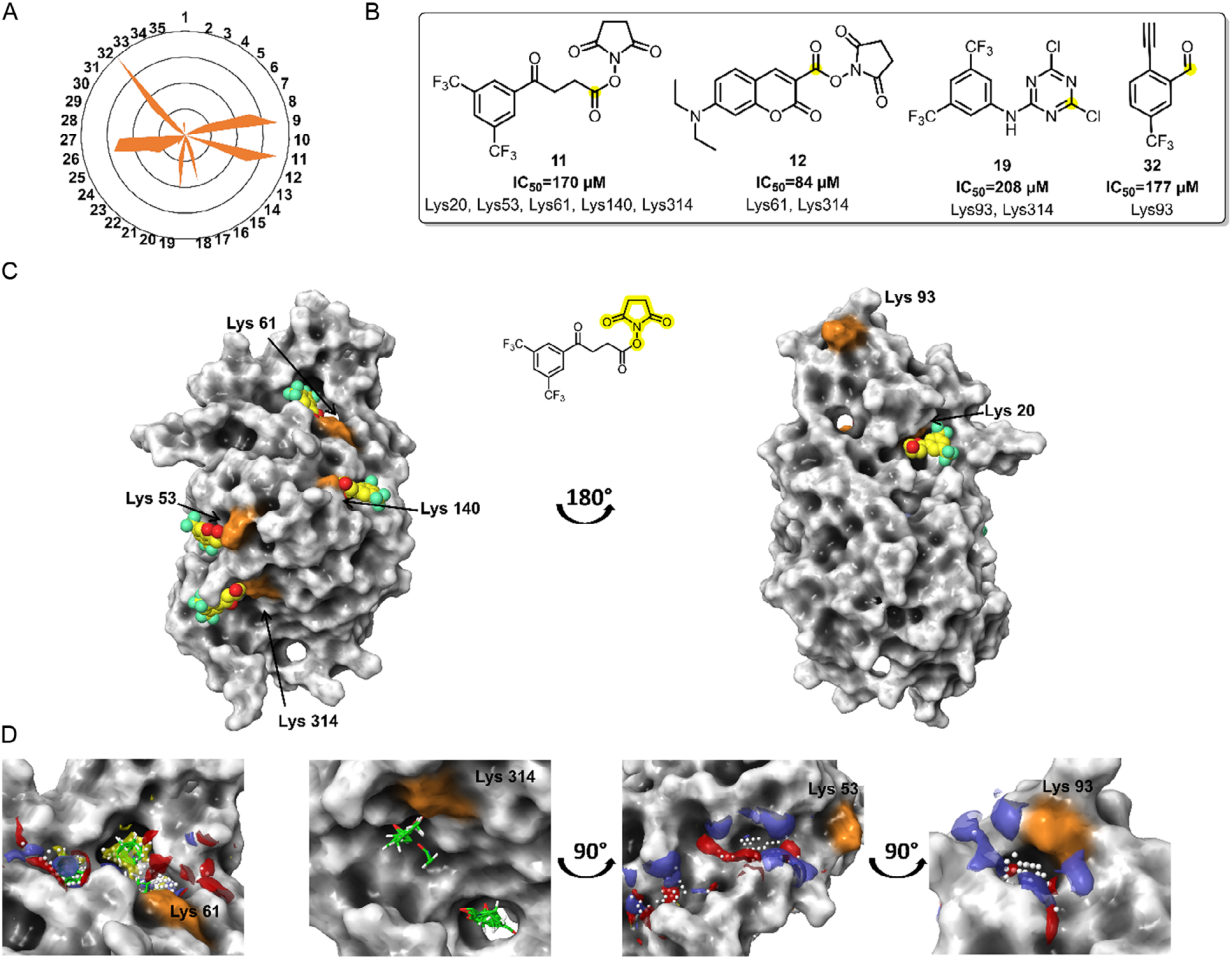
(A) Fragment reactivity pattern on HDAC4 based on limit‐dose screening at 250 µM after 1 h pre‐incubation at 3 °C, data is represented as mean from triplicates. (B) Active hits displayed for which covalent labelling and digestion experiments were successful. The corresponding IC_50_ values and labelled residues are indicated. (C) Structure of HDAC4 (PDB ID: 2VQJ [48]) with the labelled lysine residues highlighted in orange, with the covalent docking results of **11** indicated by a thick tube representation. Lys20 is located at the regulatory Zn^2+^ binding domain, which is a known allosteric site of HDAC4 [49]. Lys20 corresponds to Lys664 on full‐length HDAC4, and so Lys53 = Lys697, Lys61 = Lys705, Lys93 = Lys737, Lys140 = Lys784, Lys314 = Lys958. (D) Surface of HDAC4 with sites identified by SiteMap and FTMap. Labelled lysines are shown in orange, hydrogen‐bond donor regions are shown in blue, hydrogen‐bond acceptor regions are shown in red, the hydrophobic regions are shown in yellow.

Histone deacetylases (HDACs) are divided into four distinct classes: I, II, III, and IV, based on the size, sequence homology, and formation of distinct complexes [43, 44]. HDAC4 belongs to class IIa HDACs, which are primarily expressed in the brain, heart and skeletal muscle tissue, and shuttles between the nucleus and cytoplasm, playing a major role in tissue growth and physiological development [43]. HDAC4 is recognised as a target for neurodegenerative diseases [44], in particular, Huntington's disease [45, 46] and in different types of cancer [47]. Compared to class I HDACs, class IIa HDACs have ~1000‐fold lower enzymatic activity due to a Tyr‐to‐His substitution in the active site. Furthermore, the lack of identified natural substrates suggests that the catalytic domain mainly takes part in PPIs (e.g. coordinating the activity of class I HDACs [47]) and macromolecular signalling rather than deacetylation [46]. HDACs are zinc‐dependent enzymes containing a catalytic zinc ion in the active site and another zinc in the structural zinc‐binding domain, which is known to be highly flexible and plays an important role in substrate recognition [48]. In contrast to the limited selectivity of Zn‐coordinating ligands, selective modulation of HDAC4 is particularly desirable and can be linked to therapeutic benefits [44]. Allosteric binding even to regulatory domains represents a promising strategy to achieve this selectivity. Therefore, we screened the lysine‐reactive fragment library in a biochemical assay and by MS to identify targetable and functional lysine residues that could allow allosteric modulation. We performed a single‐concentration screen at 250 µM to identify compounds that reduced HDAC4 activity by more than 50% (Figure 5A). IC_50_ values were then determined for the resulting hits. To further validate these results, we conducted intact MS measurements. For the hits (**11**, **12**, **19**, **26**, and **32**), we subsequently performed peptide mapping, through which we were able to identify labelled peptides for four of the compounds (**11**, **12**, **19**, and **32**). The catalytic domain of HDAC4 contains 20 lysine residues, six of which were successfully labelled in this study, namely Lys20, Lys53, Lys61, Lys93, Lys140, and Lys314. Fragment **11** modified five of these six lysines, except Lys93 (Figures 5C and S4). NHS ester **12** labelled Lys61 and Lys314, while the dichlorotriazine **19** labelled Lys314 as well as the surface‐exposed Lys93. The ethynyl aldehyde **32** also modified Lys93. The labelled lysines represent potential sites for allosteric targeting. Particularly, Lys20 is known to be located within a previously characterised allosteric site at the regulatory Zn^2+^ binding domain that is able to accommodate drug‐sized molecules (400 Å^3^) based on computations using SiteMap and FTMap (Figure S5). This is also the site where the potent inhibitor tasquinimod binds, which locks the protein in a conformation preventing HDAC4/N‐CoR/HDAC3 complex formation [49]. In addition, FTMap and SiteMap identified three cavities with diverse polarities being part of a similarly large pocket near Lys61 those together might be able to accommodate lead‐like molecules (Figure 5D), while Lys93 lies in a small cavity in the protein surface that fits to the size of the labelling fragment. Near Lys53, another pocket was found by SiteMap, without direct contact between Lys53 sidechain atoms and the pocket, a more extended but shallow pocket was identified by FTMap near Lys314.

### 2.6 Mapping an Oncogenic PPI Target: KRAS^G12D^

**FIGURE 6:**
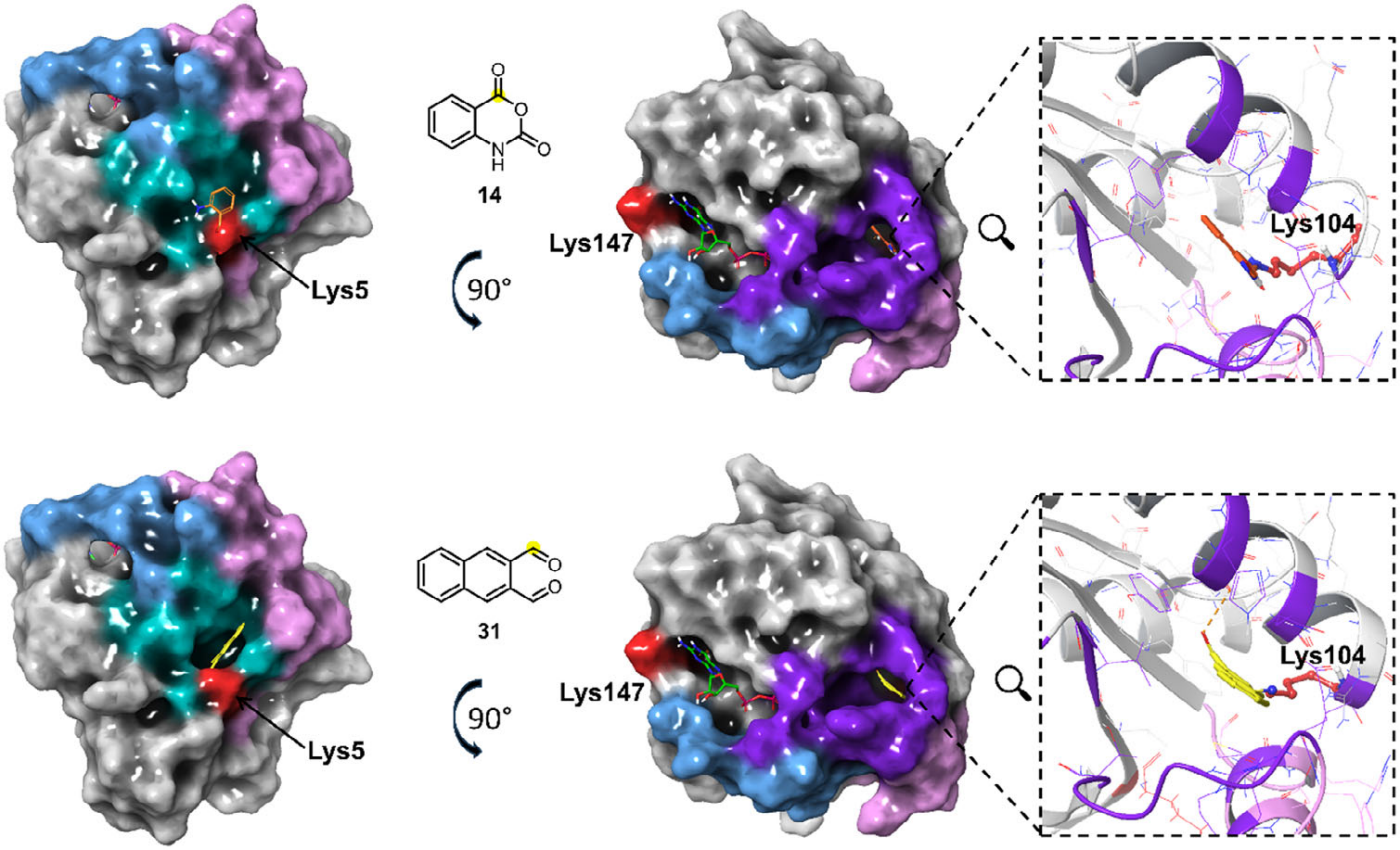
Labelled lysines by the hit compounds identified in the peptide mapping experiments are highlighted in red. Induced fit docking results of the two compounds (**14**, **31**), which labelled lysines at known allosteric sites. The defined regions are highlighted in purple (Switch II pocket), turquoise (Switch I/II pocket region), blue (Switch I region), and light pink (Switch II region).

Kirsten rat sarcoma viral oncogene homologue (*KRAS*) is one of the most frequently mutated oncogenes. It encodes the KRAS protein [50], a key regulator of cell signalling pathways that control proliferation and survival, which functions through specific PPIs with downstream effectors [50, 51]. Aberrant KRAS signalling promotes uncontrolled growth and remains a major focus of targeted cancer therapy development [52]. The most prevalent oncogenic mutations in KRAS occur on the 12th glycine, with aspartic acid being the most common point‐mutation (G12D, 41%), followed by valine (G12V, 28%) and cysteine (G12C, 14%) [53, 54]. Since KRAS was considered undruggable for a long time, the development of two cysteine‐based mutant‐selective covalent drugs, sotorasib [55] and adagrasib [56] represented major breakthroughs in directly targeting the KRAS^G12C^. However, significant efforts have continued to overcome drug resistance and extend targeting to other KRAS variants [57]. Hence, our goal was to map lysines on KRAS^G12D^ and provide alternative sites available for covalent modification. First, we screened our fragment library using intact MS, leading to the identification of eight hits (Table S2). Three of these (**14**, **27** and **31**) exceeded >10% labelling (42%, 54% and 100%, respectively) and were selected for peptide mapping. Enzymatic digestion was successful for hits **14** and **31**, for which we identified covalent modification on the same three lysine residues for both compounds: Lys5, Lys104, and Lys147, respectively. Next, we investigated the binding mode of these hits using induced‐fit docking (Figure 6). Lys5 and Lys104 are part of the RAS‐GAP protein–protein interaction interface. In particular, Lys5 is positioned in a channel adjacent to the Switch I/II pocket, a recently identified promising allosteric site [58] (Figure 6, turquoise, Figure S6), and Lys104 lies in the Switch II pocket (sotorasib binding site, Figure 6, purple, Figure S6) [59]. Lys147 is located at the edge of the GDP‐binding site and is more surface exposed, but still belongs to the large Switch II binding pocket that is able to accommodate even drug‐sized molecules (Figure S7). These results highlight promising opportunities for the covalent modulation of KRAS–protein interactions and might provide covalent anchoring sites for allosteric KRAS^G12D^ inhibitors.

### 2.7 Mapping an E3 Ligase: FBW7

**FIGURE 7:**
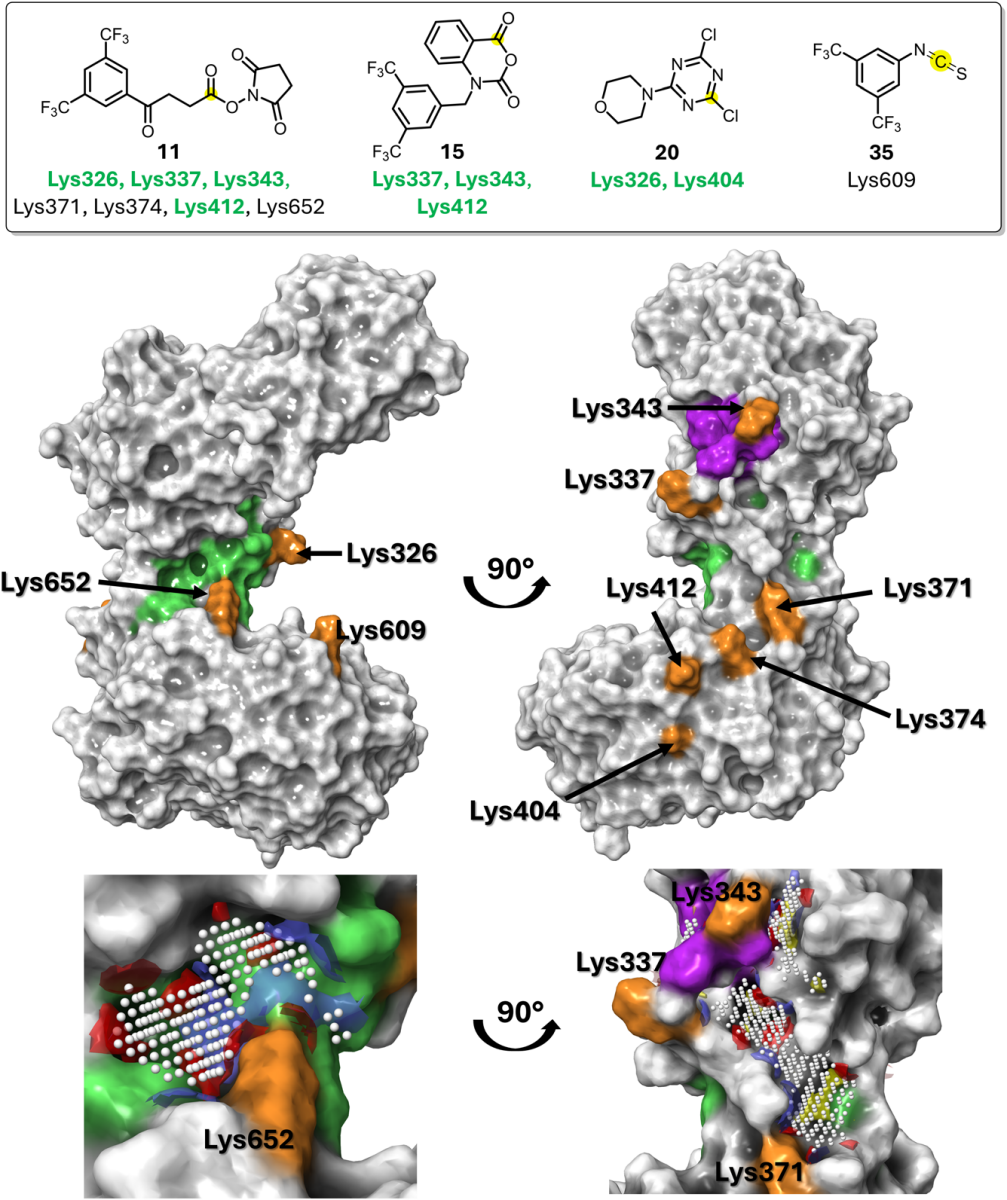
Active hits displayed for which covalent labelling and digestion experiments were successful, indicating labelled surface (black) or functionally relevant (green) lysines. FBW7‐SKP complex (PDB ID: 2OVP [62]) structure coloured by functional sites (purple – Pocket D, green – Pocket G) and labelled lysines (highlighted in orange on the surface) identified in peptide mapping experiments. Sites identified by SiteMap are coloured as follows: hydrogen‐bond donor regions are shown in blue, hydrogen‐bond acceptor regions are shown in red, and the hydrophobic regions are shown in yellow.

E3 ligases play a central role in regulating the ubiquitin–proteasome system (UPS) and have become promising targets for highly specific induced proximity‐based therapeutic strategies [60]. FBW7 is one of the most frequently deregulated components of the UPS in human cancers due to its function in cellular growth and division pathways, including the degradation of c‐MYC, cyclin E, NOTCH, and JUN [61]. FBW7 functions as a tumour suppressor by targeting several oncogenic proteins for degradation, and mutations in its gene occur in approximately 6% of all cancers [61]. Despite its biological importance, FBW7 remains a challenging protein target. Recent computational analyses and mapping by photoactivable fragments revealed previously unrecognised binding sites (pockets D and G) with potential allosteric effects that may enable ligand discovery against this E3 ligase [60]. Thus, we screened our library by intact MS and identified 5 hits that labelled FBW7. We then performed tryptic digestion to identify the labelled sites (Figure 7 for lysines, Figure S8 for docking results, and Figure S9 for further residues). Isothiocyanate **35** labelled a surface lysine (Lys609) and two cysteines (Cys493, Cys533), while sulphonyl fluoride **3** labelled a tyrosine within pocket G (Tyr355). On the contrary, NHS ester **11**, isatoic anhydride **15**, and dichlorotriazine **20** showed lysine selectivity. The NHS ester **11**, corresponding to its high reactivity, labelled seven lysines, among them three surface lysines and one lysine at most sites, particularly targeting the already identified pockets D, G, and a Lys‐rich pocket on the WD40 domain. Isatoic anhydride **15** labelled two lysines in pocket D (Lys337 and Lys343), suggesting its affinity to that site, while dichlorotriazine **20** labelled Lys326 adjacent to pocket G. In addition, both **15** and **20** labelled functionally relevant lysines on the WD40 domain (Lys412 and Lys404, respectively), which are crucial for FBW7 autoubiquitylation, and TRIP12 protein–protein interaction [63]. Our computations revealed that pocked D is large enough to accommodate drug‐like compounds (Figure 7), especially given that the labelled lysines are located within a relatively large loop, which allows substantial movements, this site is able to extend toward Lys371. In addition, a cavity was identified on the protein surface around Lys652 that might be able to accommodate lead‐like molecules. SiteMap also identified pocket G (Figure S10), a previously described [60] targetable binding site. Our results support that lysine‐targeting can be a useful approach to map E3 ligases and to identify new anchoring points for covalent labelling that could be a useful information in targeted protein degradation (TPD). In PROTAC development, ‘binder‐first’ E3 ligase‐centred approaches may offer a promising option for expanding the current limited set of targeted E3 ligases, regardless of their family function and/or structure [60].

## 3 Conclusion

Covalent fragments are small electrophilic species with mostly reactivity‐driven binding, making them useful to characterise protein residues available for covalent labelling and prioritising potential warheads [4, 11]. Targeting lysine residues has emerged as an exciting and expanding area of drug discovery. While cysteine traditionally dominates covalent strategies due to its high nucleophilicity, lysine offers several complementary advantages. Its *ε*‐amino group is abundant and often positioned at functionally relevant regions, including active sites, protein–protein interfaces, and regulatory pockets. Moreover, it is less susceptible to oxidative modifications [64]. The distinct pK_a_ and microenvironment‐dependent reactivity of lysine provide opportunities for selective engagement with carefully tuned electrophiles. Screening covalent fragment libraries is particularly valuable in this context, as they enable the rational mapping of tractable residues, the systematic evaluation of diverse reaction mechanisms and fine‐tuning electrophilic warhead reactivity [4].

In this study, we show that screening a small library of electrophiles provides useful reactivity and accessibility information on targeted lysines across diverse proteins using different screening paradigms. We have compiled a library of covalent fragments sampling the aminophilic warhead space and have screened a set of challenging protein targets that were previously not targeted by Lys‐covalent approaches. We have labelled a functional active site Lys in the antibacterial target DdlB and an allosteric lysine in the epigenetic regulator HDAC4 and E3 ligase FBW7. We have successfully inhibited the transcription factor STAT3 by labelling lysines available at the SH2 domain and have covalently modified protein–protein interaction surfaces of KRAS^G12D^ oncogenic mutant. We have identified functionally relevant lysine and warheads suitable for labelling these targets. Furthermore, exploring their binding sites provides information to the rational design of selective and potent covalent modulators.

## Author Contributions

N.Cs. compiled the library, measured biology for STAT3, measured and analysed protein MS and wrote the manuscript. N.P and L.K. participated in protein MS measurements. G.S., T.I., and J.S. performed enzymatic digestions and MS/MS measurements. P.S. developed the MS methods. Z.O., T.V.S., V.I., and G.G.F. have performed molecular docking studies. P.Á.‐B. supervised the work, analysed data and contributed to manuscript writing. M.H. measured DdlB biology, A.K. and M.N. measured HDAC4 biology. C.G. and F.‐J.M.‐A. provided funding. G.M.K. conceptualised the project, supervised the work, provided funding and wrote the manuscript.

## Funding

This work was supported by the Nemzeti Kutatási Fejlesztési és Innovációs Hivatal (RRF‐2.3.1‐21‐2022‐00015 and STARTING_25 152137), Schweizerischer Nationalfonds zur Förderung der Wissenschaftlichen Forschung (MAPS 230109), Ministerio de Ciencia, Innovación y Universidades (PID2021‐127693OB‐I00), and H2020 Marie Skłodowska‐Curie Actions (956314 (ALLODD)).

## Conflicts of Interest

The authors declare no conflicts of interest.

## Supporting information

Supporting Information contains a scheme of reaction mechanisms, reactivity and stability data, raw data of labelling and bioactivity, additional figures of computations, materials, methods and experimental procedures. The authors have cited additional references within the Supporting Information. [in the following order: [65, 66, 13, 40, 67, 62, 31, 30, 68, 60, 69, 70, 22, 21, 19, 20, 48, 71, 72]

## Data Availability

The data that supports the findings of this study are available in the Supporting Information of this article.
