# Supplementary material for: Mapping Functionally Relevant Tractable Lysines of Challenging Protein Targets by Covalent Fragment Screening
Source: Chembiochem. 2026 Jun 11;27(11):e70421. doi: 10.1002/cbic.70421 (PMC13261308; doi:10.1002/cbic.70421)
Supplement: Supplementary file 1 — Supporting Information contains a scheme of reaction mechanisms, reactivity and stability data, raw data of labelling and bioactivity, additional figures of computations, materials, methods and experimental procedures. The authors have cited additional references within the Supporting Information. [in the following order: [65, 66, 13, 40, 67, 62, 31, 30, 68, 60, 69, 70, 22, 21, 19, 20, 48, 71, 72] [file CBIC-27-e70421-s001.pdf]

Supporting Information

Mapping Functionally Relevant Tractable Lysines of Challenging Protein Targets by Covalent Fragment Screening

Authors

Noémi Csorba[a,b,c], Péter Ábrányi-Balogh[a,b,c], Zoltán Orgován[a,b], Levente Kollár[a,b], Nikolett Péczka[a,b], György G. Ferenczy[a,b], Tibor Viktor Szalai[a,b,d], Tímea Imre[a,b,e], József Simon[a,b,e], Pál Szabó[e], Gitta Schlosser[f], Martina Hrast[g], Stanislav Gobec[g], Varbina Ivanova[h,i], Carles Galdeano[h,j], Aleksandra Kopranovic[k], Marc Neumann[k], Franz-Josef Meyer-Almes[k], György Miklós Keserű*[a,b,c]

Affiliations

a) Medicinal Chemistry Research Group, HUN-REN Research Centre for Natural Sciences, Magyar tudósok krt 2, H-1117 Budapest, Hungary
b) National Laboratory for Drug Research and Development, HUN-REN Research Centre for Natural Sciences, Magyar tudósok krt 2, H-1117 Budapest, Hungary
c) Department of Organic Chemistry and Technology, Faculty of Chemical Technology and Biotechnology, Budapest University of Technology and Economics, Műegyetem rkp. 3., H-1111 Budapest, Hungary
d) Department of Inorganic and Analytical Chemistry, Faculty of Chemical Technology and Biotechnology, Budapest University of Technology and Economics, Műegyetem rkp. 3., H-1111 Budapest, Hungary
e) MS Metabolomics Research Group, HUN-REN Research Centre for Natural Sciences, Magyar tudósok krt 2, H-1117 Budapest, Hungary
f) MTA-ELTE Lendület (Momentum) Ion Mobility Mass Spectrometry Research Group, ELTE Eötvös Loránd University, Institute of Chemistry, Pázmány Péter sétány 1/A, H-1117 Budapest, Hungary
g) Department of Pharmaceutical Chemistry, Faculty of Pharmacy, University of Ljubljana, 1000 Ljubljana, Slovenia
h) Departament de Farmàcia i Tecnologia Farmacéutica, i Fisicoquímica. Facultat de Farmàcia i Ciències de l'Alimentació Universitat de Barcelona, Av. Joan XXIII 27-31, 08028 Barcelona, Spain
i) Institut de Química Teòrica i Computacional (IQTC), Universitat de Barcelona, c/ Martí i Franqués 1, 08028 Barcelona, Spain
j) Institut de Biomedicina de la Universitat de Barcelona (IBUB), Universitat de Barcelona, Av. Diagonal 643, 08028 Barcelona, Spain
k) Department of Chemical Engineering and Biotechnology, University of Applied Sciences Darmstadt, Haardtring 100, 64295 Darmstadt, Germany

E-mail: *keseru.gyorgy@ttk.hu

Table of contents

1.1 Supporting figures and tables 3

1.2 Methods 11

1.2.1 HPLC-MS-based *N*-α-acetyl-lysine reactivity assay[3] 11

1.2.2 HPLC-MS-based assessment of stability at pH 7.4[3] 11

1.2.3 DdlB residual activity measurements and determination of IC_{50} values 12

1.2.4 STAT3 fluorescence polarization assay 12

1.2.5 HDAC4 catalytic domain assay 13

1.2.6 Mass spectrometry experiments 14

1.2.6.1 Sample preparation of* DdlB, cHDAC4, STAT3, $KRas^{G12D}$ and FBW7 *for intact protein LC-MS screen 14

***1.2.6.2 Sample preparation for digestion of* DdlB, cHDAC4, $KRas^{G12D}$ and FBW7** 14

1.2.6.3 Intact mass spectrometry measurements of DdlB, STAT3, cHDAC4, $KRas^{G12D}$ and FBW7 14

1.2.6.4 Digestion and LC-MS/MS measurements of DdlB, cHDAC4 and FBW7 samples on TripleTOF 5600+ instrument 15

1.2.6.5 Digestion and LC-MS/MS measurements of $KRas^{G12D}$ samples on Waters instrument 16

1.2.7 Computational methods 16

1.2.7.1 DdlB 16

1.2.7.2 HDAC4 17

1.2.7.3 STAT3 17

1.2.7.4 $KRas^{G12D}$ 17

1.2.7.5 FBW7 18

1.3 Results 19

1.3.1 Dose-response curves for DdlB 19

1.3.2 Intact mass spectrometry results of DdlB 19

1.3.3 Digestion results of DdlB 21

1.3.4 Dose-response curves for STAT3 26

1.3.5 Intact mass spectrometry results of STAT3 27

1.3.6 Dose-response curves for cHDAC4 28

1.3.7 Intact mass spectrometry results of HDAC4 30

1.3.8 Digestion results of HDAC4 31

1.3.9 Intact MS results of $KRas^{G12D}$ 35

1.3.10 Digestion results of $KRas^{G12D}$ 37

1.3.11 Intact MS results of FBW7 40

1.3.12 Digestion results of FBW7 41

1.4 Bibliography 48

1.1 Supporting figures and tables

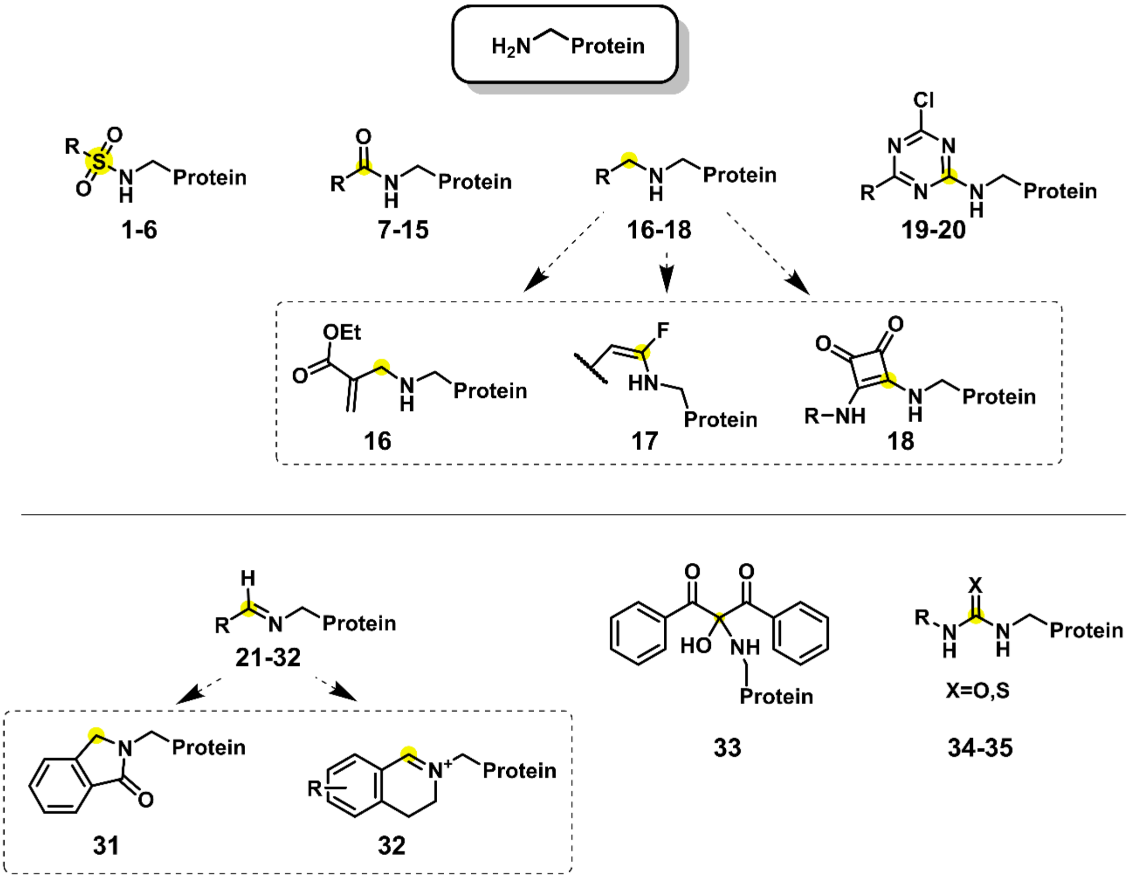

Figure S1. Presumed reactivity modes of the warheads and general formula of the formed adduct with the ε-amino group of lysine at a protein binding site for each warhead type. In the case of **31** and **32** the product will further react and result in an isoindolinone[1] and an isoquinolium[2], respectively.

ID	Warhead type	k_{Lys} pH 10.2 [h^{-1}]	k_{Blank} pH 10.2 [h^{-1}]	$k_{Binding}$ pH 10.2 [h^{-1}]	Lys-reactivity $t_{1/2}$, pH 10.2 [h]	Stability $t_{1/2}$, pH 10.2 [h]	Stability, pH 7.4 [%]
**1**	Sulfonyl chloride	3.5936	1.8704	1.7231	0.40	0.37	66%
**2**	Sulfonyl chloride	-	-	-	<1min	<5min	8%
**3**	Sulfonyl fluoride	-	22.3021	-	<1min	0.03	98%
**4**	Fluorosulfate	1.3672	0.1402	1.2271	0.56	4.94	65%
**5**	Sulfonyl heterocycle (triazole)	1.2901	0.6490	0.6410	1.08	1.07	93%
**6**	*N,O*-sulfonate	-	2.0090	-	<5min	0.35	10%
**7**	*N*-Acyl-*N*-alkyl sulfonamide (NASA)	49.9687	14.6843	35.2845	0.02	0.05	80%
**8**	*N*-Acyl-*N*-alkyl sulfonamide (NASA)	6.6113	3.9798	2.6315	0.26	0.17	60%
**9**	Benzothiazinone	0.0383	0.0293	0.0089	77.96	23.62	98%
**10**	*N*-succinimidyl ester	-	-	-	<1min	<1min	52%
**11**	*N*-succinimidyl esters	-	-	-	<1min	<5min	35%
**12**	*N*-succinimidyl ester	-	8.3649	-	<1 min	0.08	1%
**13**	Bisacetanilide	3.0802	0.4796	2.6006	0.27	1.45	45%
**14**	Isatoic anhydride	-	2.0545	-	<1 min	0.34	41%
**15**	Isatoic anhydride	-	26.5429	-	<1min	0.03	51%
**16**	sulfonyl acrylate	29.8739	0.4554	29.4185	0.02	1.52	87%
**17**	Difluorostyrene	0.3872	0.2487	0.1385	1.28	3.32	96%
**18**	Squarate	6.1278	0.0570	6.0708	0.11	12.17	34%
**19**	Dichlorotriazene	8.8615	0.7139	8.1475	0.09	0.97	95%
**20**	Dichlorotriazene	41.1428	0.1143	41.0284	0.02	6.06	86%
**21**	Aldehyde	0.0012	0.1225	-	24+	5.66	83%
**22**	Heterocyclic aldehyde	0.0045	0.0061	-	24+	113.65	94%
**23**	*N*-formyl heterocycle	0.0297	0.0046	0.0251	27.67	150.39	82%
**24**	Ketoaldehyde (enolate)	0.0064	0.0196	-	24+	35.37	100%
**25**	Ketoaldehyde	0.0150	0.0045	0.0105	65.85	154.23	97%
**26**	Hydroxy aldehyde (salicylaldehyde)	0.0197	0.0028	0.0169	40.93	246.76	96%
**27**	Acylphloroglucinol (salicylaldehyde)	15.8164	0.0339	15.7825	0.04	20.46	86%
**28**	Formylphenyl boronic acid	0.0835	0.0640	0.0196	35.41	10.84	97%
**29**	Formylphenyl boronic acid	0.0078	0.0016	0.0063	110.54	447.17	97%
**30**	Formylphenyl boronic ester	-	-	-	-	<1min	19%
**31**	*ortho*-Phthalaldehyde (OPA)	-	0.0593	-	<1min	11.69	82%
**32**	Ethynyl aldehyde/formylacetylene	3.0240	0.0451	2.9789	0.23	15.36	98%
**33**	Polycarbonyl	-	-	-	-	<1min	66%
**34**	Isocyanate	-	-	-	<1min	<5min	73%
**35**	Isothiocyanate	6.4275	0.5288	5.8987	0.13	1.04	80%

Table S1. Overview of the warhead types and results of fragment reactivity profiling.[3] Fragments were evaluated using an HPLC-based surrogate kinetic assay against *N*-α-acetyl-lysine, at pH 10.2 to determine pseudo first-order rate constants (k) and the corresponding half-lives ($t_{1/2}$). In the case of competing reactions (reaction with *N*-α-acetyl-lysine and degradation), the apparent rate constant is defined as $k_{Lys} = k_{Blank} + k_{Binding}$. Experimentally determined half-lives were calculated as $t_{1/2}(Lys) = \ln 2/(k_{Lys})$. Reactions were monitored for 24

h and performed in duplicates. The compounds' stability was also evaluated at pH 7.4 calculating the % decomposition of the compound within 1 h. Reactions were monitored at 0 and 1 h and performed in duplicates.

ID	DdlB @500 µM	DdlB intact%	cHDAC4 @250 µM	cHDAC4 intact%	STAT3 @500 µM	STAT3 @50 µM	STAT3 intact%	KRAS^{G12D} intact%	FBW7 intact%
1	1±7%	-	12±1%	-	13±4%	0±0%	-	0	0
2	0±5%	-	0±4%	-	17±10%	0±9%	-	0	0
3	23±8%	-	0±3%	-	0±7%	0±4%	-	0	22
4	72±3%	0	-	-	10±7%	9±9%	-	0	0
5	0±9%	-	0±4%	-	20±5%	5±4%	-	0	0
6	14±9%	-	3±9%	-	5±4%	0±6%	-	0	0
7	28±6%	-	19±8%	-	28±4%	0±7%	-	0	0
8	42±6%	-	65±8%	0	0±0%	0±8%	-	0	0
9	30±9%	-	92±1%	0	12±10%	10±9%	-	0	0
10	54±7%	21	0±8%	-	100±9%	59±3%	75*	10	0
11	32±8%	-	91±8%	29*	89±6%	51±8%	50*	8	54*
12	95±2%	92*	67±5%	38*	59±5%	20±2%	-	9	0
13	35±6%	-	0±2%	-	12±4%	3±1%	-	0	0
14	68±8%	81*	0±4%	-	100±9%	51±2%	80*	42	0
15	2±10%	-	12±1%	-	6±4%	4±5%	-	0	11
16	2±9%	-	14±7%	-	6±6%	5±0%	-	0	0
17	35±6%	-	45±1%	-	27±4%	0±2%	-	0	0
18	48±6%	-	6±8%	-	14±3%	0±5%	-	0	0
19	48±7%	-	54±1%	15	76±4%	56±2%	19	7	0
20	16±8%	-	23±8%	-	4±8%	5±6%	-	0	55
21	22±8%	-	0±3%	-	5±8%	3±14%	-	0	0
22	27±8%	-	11±10%	-	11±3%	5±4%	-	0	0
23	0±5%	-	8±2%	-	23±10%	0±8%	-	0	0
24	4±9%	-	30±4%	-	0±5%	0±9%	-	0	0
25	31±7%	-	37±4%	-	0±7%	0±4%	-	0	0
26	52±6%	14	68±2%	29	97±8%	51±4%	12	0	0
27	44±5%	-	64±5%	0	9±5%	0±8%	-	54	0
28	44±5%	-	0±5%	-	0±6%	0±7%	-	0	0
29	25±7%	-	0±9%	-	7±13%	5±5%	-	0	0
30	28±7%	-	8±9%	-	2±3%	0±7%	-	0	0
31	100±3%	17	38±1%	-	90±2%	70±7%	15	100*	0
32	0±1%	-	98±4%	22	22±4%	0±0%	-	0	0
33	40±4%	-	2±6%	-	42±8%	2±9%	-	0	0
34	44±5%	-	0±9%	-	18±1%	4±6%	-	0	0
35	24±3%	-	9±9%	-	23±6%	13±3%	-	0	70

Table S2. Screening results obtained in Method A and Method B. In case of DdlB, cHDAC4 and STAT3 only the functionally relevant hits (>50% inhibition in the single point screen, highlighted in green) were evaluated in intact MS, whereas for KRAS^{G12D} and FBW7 the primary covalent hits identified by MS screening are shown. Labelling% increase is shown by deeper shade of blue, the "*" indicates multiple labelling events. Inhibition data are shown as mean ± S.D from technical duplicates in the case of DdlB, and triplicates in the case of cHDAC4 and STAT3. Detailed descriptions of the experiments are provided below.

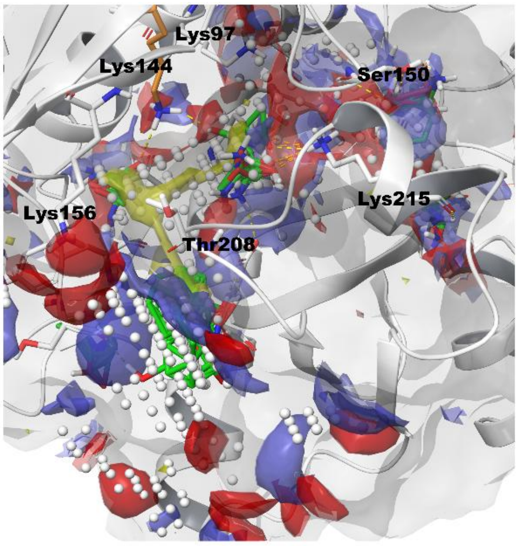

Figure S2. Active site of DdlB found by SiteMap and FTMap. Labelled lysine is shown in orange, hydrogen-bond donor regions are shown in blue, hydrogen-bond acceptor regions are shown in red, the hydrophobic regions are shown in yellow. FTMap ligands are shown as green sticks.

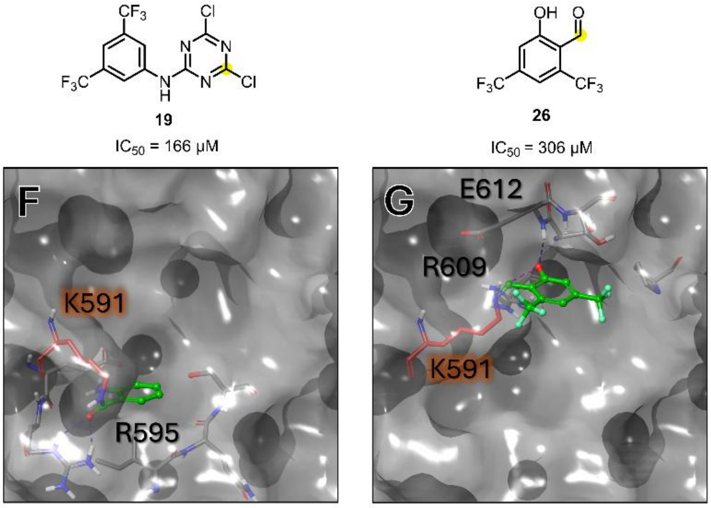

Figure S3. Docking result of STAT3 hits above IC_{50} > 100 μM. Predicted best-scoring binding poses for compound **19** (F) and compound **26** (G) resulted from covalent docking (PDB: 6QHD[4]). Targeted K591 residue is colored red, and interacting residues are labeled. Interactions are shown as dashed lines, with dark blue coloring corresponding to hydrogen bonds, purple coloring corresponding to halogen bonds and magenta coloring corresponding to salt bridges.

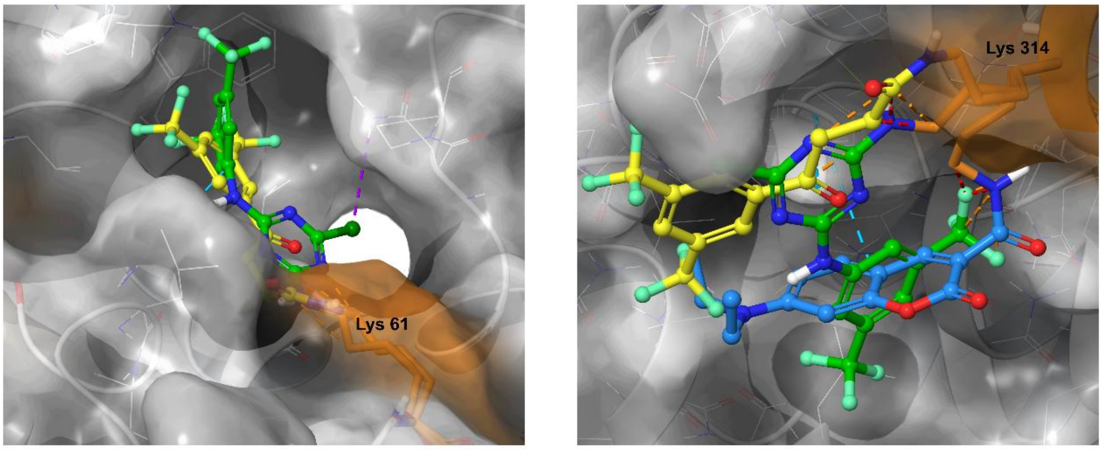

Figure S4. Covalent docking poses of 11 and 12 targeting Lys61, and 11, 12, 19 targeting Lys 314 on HDAC4. Labelled residues are coloured as orange. Docked compounds are shown as sticks (**11** – yellow, **12** – green, 19 – cyan).

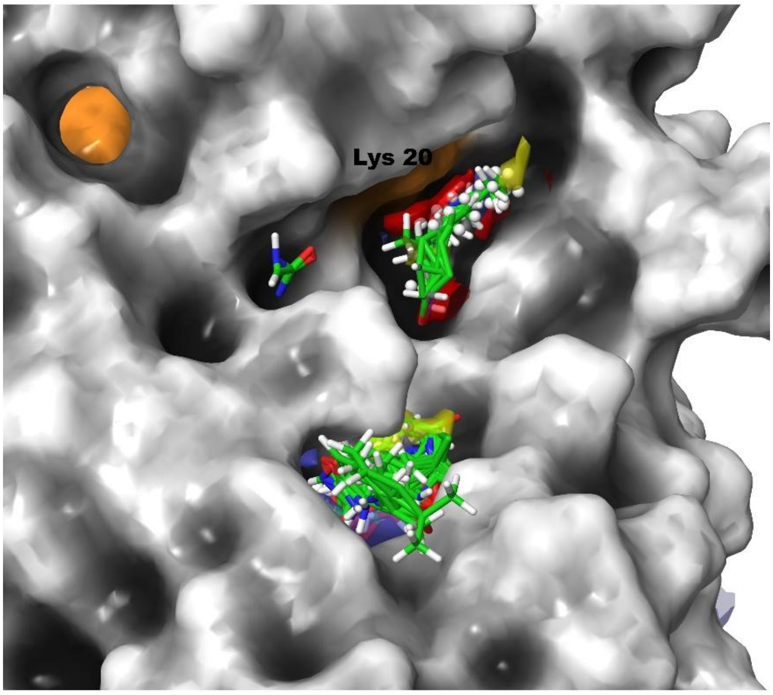

Figure S5. Lys20 site found by FTMap and SiteMap on HDAC4. Labelled lysine is shown in orange, hydrogen-bond donor regions are shown in blue, hydrogen-bond acceptor regions are shown in red, the hydrophobic regions are shown in yellow. FTMap ligands are shown as green sticks.

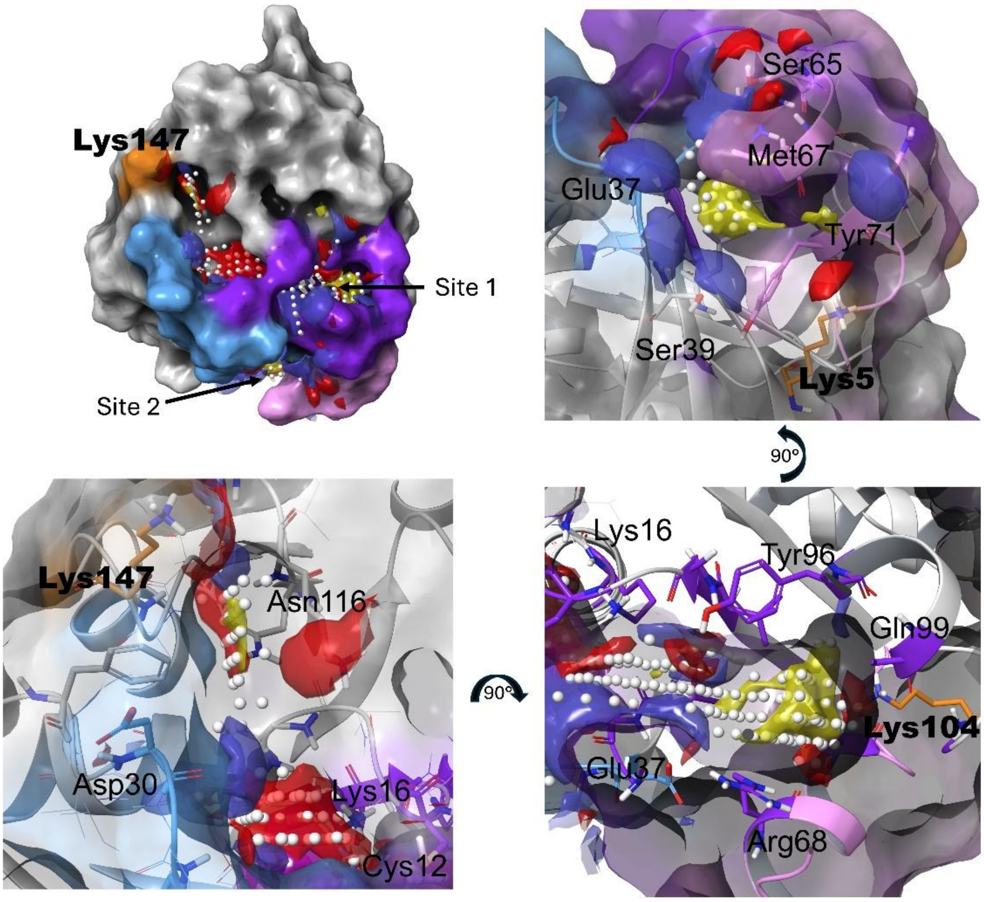

Figure S6. Sites found by SiteMap on KRAS. The defined regions are highlighted in purple (Switch II pocket), turquoise (Switch I/II pocket region), blue (Switch I region), light pink (Switch II region). Labelled lysines are shown in orange, hydrogen-bond donor regions are shown in blue, hydrogen-bond acceptor regions are shown in red, the hydrophobic regions are shown in yellow.

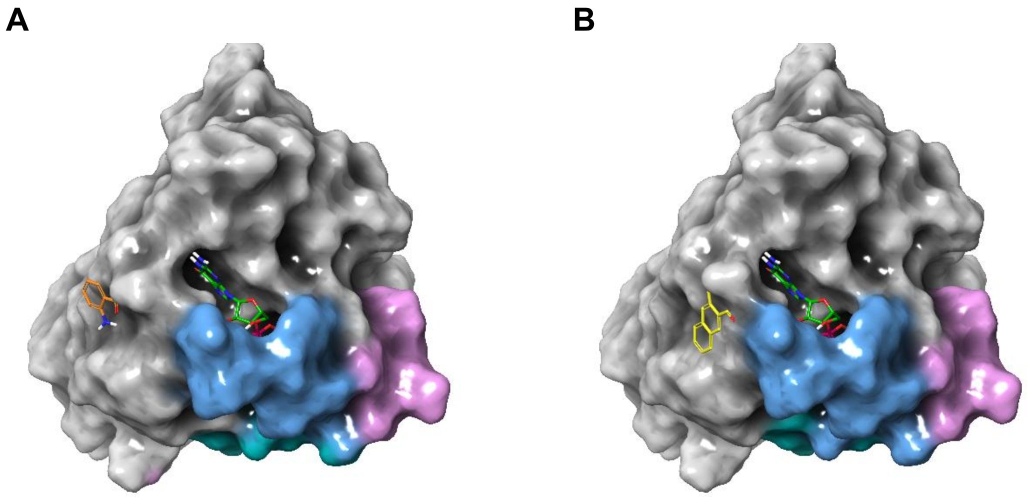

Figure S7. KRas^{G12D} (PDB ID: 6OIM[5]) labeled at Lys147 by **14** (A) and **31** (B) respectively, shown via induced fit docking. The defined regions are highlighted by turquoise (Switch I/II pocket region), blue (Switch I region) and light pink (Switch II region).

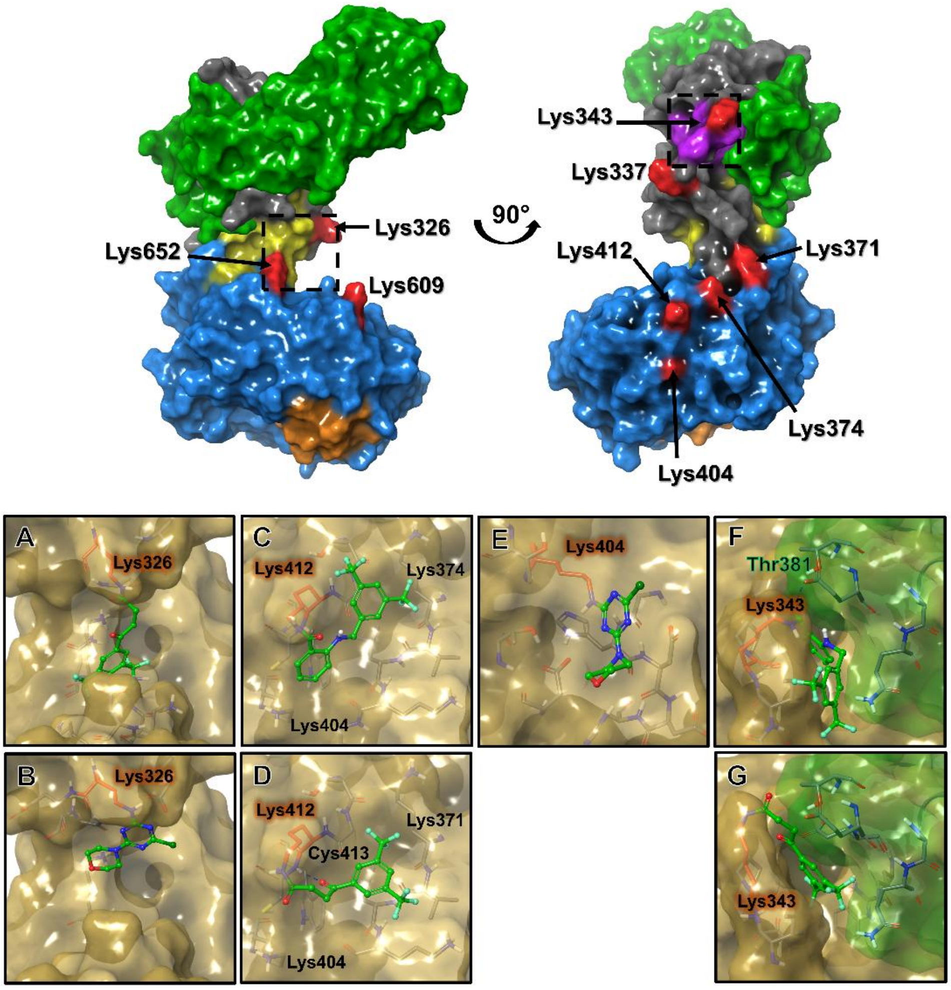

Figure S8. Predicted best scoring binding poses resulted from covalent docking (PDB: 2OVP[6]) of FBW7 (green- SKP1 interaction site, pink – Pocket D, yellow – Pocket G, orange – pocket B, blue – WD40 domain) and labelled lysines (highlighted by red on the surface). Interactions are shown as dashed lines, with dark blue coloring corresponding to hydrogen bonds. A: **11** targeting K326. B: **20** targeting K326.C: **15** targeting K412. D: **11** targeting K412. E: **20** targeting K404 F: **15** targeting K343, with interacting residue being labeled and SKP1 shown with green surface. G: **11** targeting K343 with SKP1 shown with green surface.

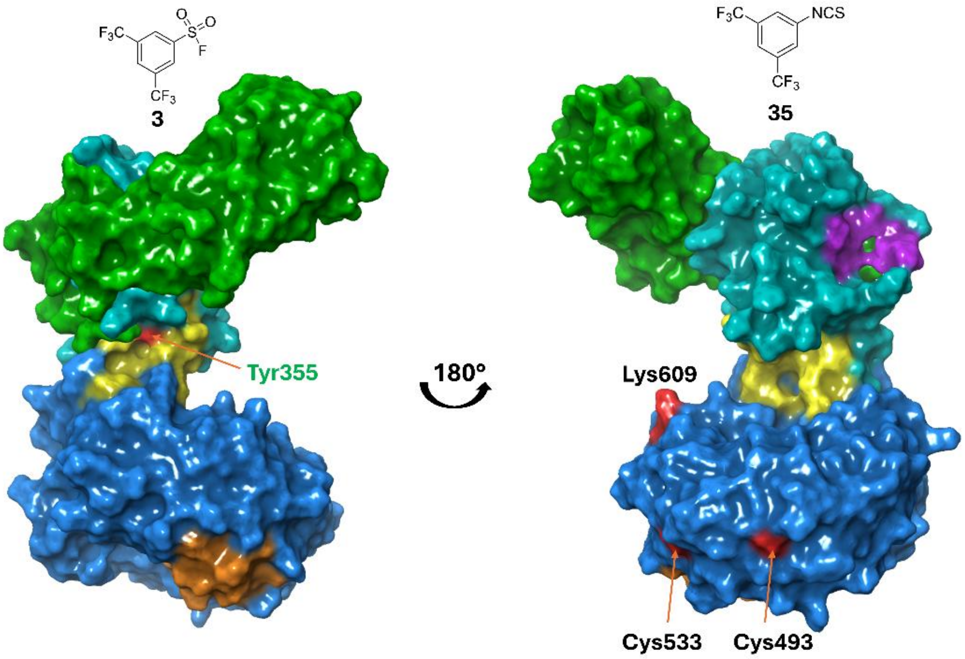

Figure S9. Compound **3** and **35** labelled residues beyond lysine. FBW7-SKP complex (PDB 2OVP)[6] structure colored by functional sites (green – SKP1 interaction site, pink – Pocket D, yellow – Pocket G, orange – pocket B, blue – WD40 domain) and labelled residues (highlighted by red on the surface) identified in peptide mapping experiments.

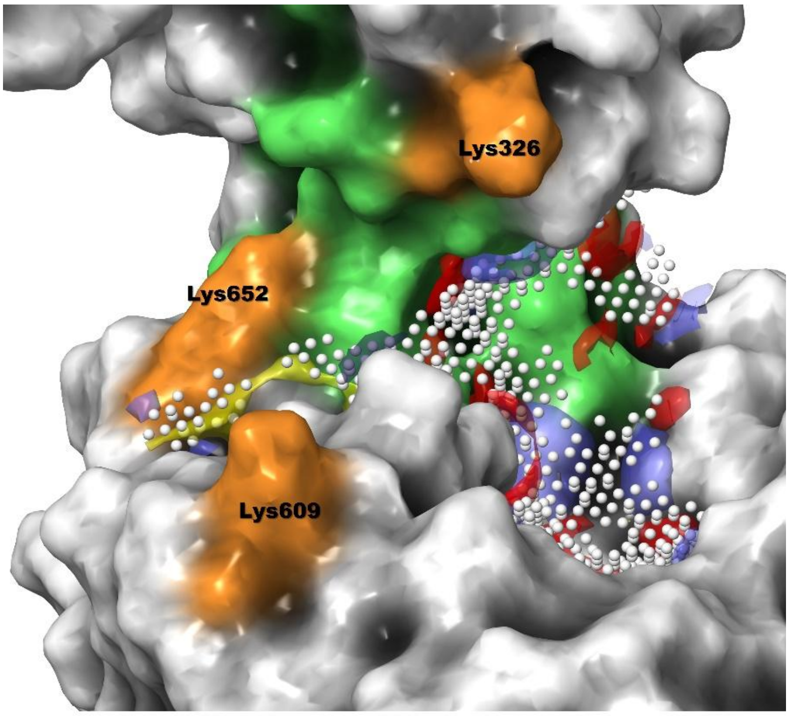

Figure S10. Sites found by SiteMap on FBW7. Pocket G amino acids are highlighted as light green. Labelled lysines are shown in orange, hydrogen-bond donor regions are shown in blue, hydrogen-bond acceptor regions are shown in red, the hydrophobic regions are shown in yellow.

1.2 Methods

The synthesis of the library members has been reported elsewhere.[3]

1.2.1 HPLC-MS-based *N*-α-acetyl-lysine reactivity assay[3]

HPLC-MS measurements were performed using a Shimadzu LCMS-2020 device equipped with a positive–negative double ion source (DUIS±) and a quadrupole MS analyzer in the range of *m/z* 50–1000. The sample was eluted with gradient elution using eluent A (0.1% HCOOH in H_2O) and eluent B (0.1% HCOOH in MeCN). The column temperature was always kept at 30 °C; the injection volume was 10 µL, and the flow rate was set to 1.5 mL/min. A Reprospher C18 (5 µm, 100 mm × 3 mm) column was used along with the following gradient. The initial condition was 0% B eluent, followed by a linear gradient to 100% B eluent by 2 min; from 2 to 3.5 min, 100% B eluent was retained. From 3.5 to 4.5 min, the initial condition with 0% B eluent was restored and retained until 5 min.

For the reactivity and stability assay, in a glass LC-MS vial the internal standard (indoprofen or papaverine or 1,4-dicyanobenzene, 50 µL, 2 mM in acetonitrile) and the 100 mM BBS buffer solution (900 µL, pH 10.2) was mixed with or without 50 mM *N*-α-acetyl-lysine (providing results of reactivity or stability, respectively). Finally, to this mixture the corresponding electrophile was added (50 µl, 20 mM in acetonitrile) right before the measurement. The vial was mixed, then analyzed by HPLC-MS (10 µL injection volume) at intervals of approximately 60 min (0-4 h, 6 h, 8 h, 12 h, 24 h). Final volume was 1 mL. In the case of highly reactive compounds, the measurement was done within 1 h, monitored every 5 minutes. The AUC (area under the curve) values were determined via integration of HPLC or MS chromatograms and then corrected with the internal standard. The fragments' AUC values were subjected to ordinary least-squares (OLS) linear regression, and to compute the important parameters (kinetic rate constant and half-life time), an Excel sheet was applied. The data are expressed as means of duplicate determination. The kinetic rate constant for the degradation and corrected *N*-α-acetyl-lysine reactivity were calculated as follows. The reaction half-life for pseudo-first-order reactions ($t_{1/2}$) is ln 2/k, where k is the reaction rate. In the case of competing reactions (reaction with *N*-α-acetyl-lysine and degradation), the apparent reaction rate is $k_{Lys} = k_{Blank} + k_{Binding}$. When half-lives are measured experimentally, $t_{1/2}(Lys) = \ln 2/(k_{Lys}) = \ln 2/(k_{Blank} + k_{Binding})$. In our case, the corrected k_{Blank} and k_{Lys} (regarding blank and *N*-α-acetyl lysine-containing samples, respectively) can be calculated by linear regression of the measured kinetic data points. The corrected $k_{Binding}$ is calculated as $k_{Lys} – k_{Blank}$, and finally, the half-life is determined using the equation $t_{1/2} = \ln 2/k$.

1.2.2 HPLC-MS-based assessment of stability at pH 7.4[3]

Next, we characterized the intrinsic stability of the compounds in HEPES buffer (pH 7.4). A 1 mM solution of the fragment in HEPES buffer (pH 7.4) with 10 % acetonitrile with a 0.1 mM

solution of indoprofen or papaverine as the internal standard was incubated (providing results of stability). The reaction mixture was analyzed by HPLC-MS sampling after 0 and 1 h, respectively. The measurement was performed in technical duplicates. The AUC (area under the curve) values were determined via integration of HPLC or MS chromatograms and then corrected with the internal standard.

1.2.3 DdlB residual activity measurements and determination of IC_{50} values

A recombinant *E. coli* DdlB enzyme (D-alanine:D-alanine ligase) was expressed in *E. coli*.[7,8] Inhibition of the enzyme was determined using an end-point malachite green assay by detecting the orthophosphate formed during the enzymatic reaction. The final mixture (50 µL) contained: 50 mM HEPES, pH 8.0, 0.005% Triton X-114, 5 mM $MgCl_2$, 6.5 mM $(NH_4)_2SO_4$, 10 mM KCl, 700 µM D-Ala, 500 µM ATP, purified DdlB (diluted in 50 mM HEPES, pH 8.0), and the test compound dissolved in DMSO. The final DMSO concentration was 5%. The compounds were first pre-incubated with the enzyme for 30 min, followed by the addition of substrates to start the reaction. After 20 min of incubation at 37 ℃, the reaction was terminated by adding Biomol[R] reagent (100 µL) and after 5 min at room temperature the absorbance was measured at 650 nm using a microplate reader (Synergy H4, BioTek Instruments, Inc., USA). A parallel experiment without the enzyme was performed to detect insoluble compounds under the assay conditions and subtracted from each measurement. All experiments were performed in duplicates. RAs were calculated with respect to blank experiments without tested compounds and with 5% DMSO. IC_{50} values were determined by measuring the residual activities in duplicate at seven different compound concentrations and calculated using GraphPad Prism (GraphPad Software, San Diego, CA, USA) and reported as mean ± standard deviation.

1.2.4 STAT3 fluorescence polarization assay

The STAT3 protein (residues 127–770) was expressed and purified as described in our recent work.[9] Fluorescence polarization assay was performed on a Molecular Devices SpectraMax iD5 Multimode Microplate Reader (San Jose, CA, USA) using Greiner black 384-well flat-bottom nonbinding microplates with 40 µL final well volumes. The fluorescent peptide (5-FAM-G(pTyr)LPQTV-$CONH_2$, purchased from GenScript Biotech Ltd., Piscataway, NJ, USA), as well as the protein were diluted with a buffer containing 50 mM NaCl, 10 mM HEPES, 1 mM EDTA, 2 mM TCEP, and 0.1% Triton X-100, pH 7.5. The final concentration of the STAT3 protein was 200 nM, and the fluorescent peptide was added at a final concentration of 5 nM. The wells were treated with varying concentrations of inhibitor compounds, with a 2% final DMSO content. Protein and inhibitors were incubated at 37 °C for 1 h. Then the fluorescence peptide was added, and the plate was subsequently incubated for another 20 min prior to the fluorescence readout (extinction wavelength: 475 nm, emission wavelength: 520 nm). The measurements were carried out using 3 parallel technical replicas. Fluorescence polarization

was calculated from the perpendicular and parallel fluorescence intensities and then plotted against concentration. The inhibitor dose-response curves were analyzed in GraphPad Prism 8.0.1 (GraphPad Software, La Jolla, CA, USA) using a normalized nonlinear regression model to determine IC_{50} values (with 95% confidence intervals). Data was normalized to control conditions, where the control containing DMSO+STAT3+FAM-peptide was defined as 0% inhibition and the control containing DMSO+FAM-peptide only was defined as 100% inhibition. Normalized values were then fitted to a sigmoidal dose-response (variable slope) curve to calculate the half-maximal inhibitory concentration (IC_{50}) for each compound reported as mean ± standard deviation.

1.2.5 HDAC4 catalytic domain assay

The assay was performed in a 96-well microtiter plate (MTP) using HDAC assay buffer (25 mM Tris-HCl, 75 mM KCl, pH 8.0, 0.0001% Pluronic). 250 µM fragment and 1 nM cHDAC4 are mixed and then incubated for 1 hour at 30 °C and 600 rpm. Then, 20 µM substrate solution (Boc-Lys-(Tfa)-AMC (7-amino-4-methylcoumarin)) is added and incubated for another hour at 30 °C and 600 rpm. To stop the reaction and develop the fluorescence signal, 0.42 mg/mL trypsin and 1.67 µM SATFMK are added, and the mixture is incubated again for one hour at 30 °C. At least three wells with positive controls (without inhibitors/fragments) and three with blanks are included. The latter consists only of buffer, substrate solution, and stop solution. The signal is then measured in the fluorescence reader at an excitation wavelength of 350 nm and an emission wavelength of 450 nm. The blank values are subtracted from all values, then the residual activities are calculated by dividing the values by the positive control and multiplying by 100%. For all fragments with a residual activity of less than 50%, the IC_{50} value is then determined.

To do this, a 1:3 dilution series is performed and subsequently, 1 nM cHDAC4 is added and incubated for one hour. Afterward, 20 µM substrate (Boc-Lys-(Tfa)-AMC) is added, incubated for one hour, and then the stop solution is added. This is carried out at 30 °C and 600 rpm. The fluorescence signal is then measured, the blank value is subtracted from the other values, and these are divided by the positive control to obtain a relative value of cHDAC4 activity between 0 and 1. The relative activity is plotted against the concentration of the fragments. A 4-parameter logistic fit is then performed using the following formula: Y = Bottom + (Top-Bottom)/(1+10^(LogIC_{50}-X)*HillSlope). The fit yields the IC_{50} value (with 95% confidence intervals) and the curves were analyzed in GraphPad Prism 8.0.1 (GraphPad Software, La Jolla, CA, USA).

All values were determined in triplicate and are reported as mean ± standard deviation.

1.2.6 Mass spectrometry experiments

1.2.6.1 Sample preparation of DdlB, cHDAC4, STAT3, KRAS^{G12D} and FBW7 *for intact protein LC-MS screen*

The KRas^{G12D} protein was expressed and purified as described in our recent work.[9]

The FBW7 protein (residue 263-707) was expressed and purified as described in our recent work.[10]

The proteins were diluted with PBS buffer (pH=7.4) to 1 mg/mL. In a microcentrifuge tube (Eppendorf Protein LoBind tube, PCR clean, withstand, capacity 0.5 mL) 0.2 µL of fragment solution in DMSO was added and diluted with the protein solution to 10 µL (protein/compound ratio was 1:25 and the final sample contained 2 % DMSO). The samples were incubated at 37 °C for 60 minutes, then diluted with MilliQ water to 0.2 mg/mL right before the measurement.

1.2.6.2 Sample preparation for digestion of DdlB, cHDAC4, KRAS^{G12D} and FBW7

The proteins were diluted with PBS buffer (pH=7.4) to 1 mg/mL. In a microcentrifuge tube (Eppendorf Protein LoBind tube, PCR clean, withstand, capacity 0.5 mL) 1 µL of fragment solution in DMSO was added and diluted with the protein solution to 50 µL (protein/compound ratio was 1:25 and the final sample contained 2 % DMSO). The samples were incubated at 37 °C for 60 minutes, then buffer exchange was performed three times with Sartorius Vivaspin 500 10000 MWCO at 12000 g for 7 minutes each time to remove the excess of the compounds. Afterwards, the concentrations were set to 1 mg/mL, and the samples were subjected to MS/MS measurement.

1.2.6.3 Intact mass spectrometry measurements of DdlB, STAT3, cHDAC4, KRas^{G12D} and FBW7

The prepared samples were transferred into microvials and the labeling was analyzed in intact mass spectrometry by using a Triple TOF 5600+ hybrid Quadrupole-TOF LC-MS/MS system (Sciex, Singapore, Woodlands) equipped with a DuoSpray IonSource coupled with a Shimadzu Prominence LC20 UFLC (Shimadzu, Japan) system consisting of a binary pump, an autosampler, and a thermostated column compartment, equipped with a Phenomenex SecurityGuard Widepore C4 4x3mm cartridge. The separation was achieved using mobile phase A (5% ACN in 0.1% formic acid) and B (95% ACN, 5% water and 0.1% formic acid) using a gradient elution. A 4 min gradient (both in solvent composition and flow rate) was used with an initial flow of 0.5 mL/min and 10% eluent B. A 2 min linear increase was applied to reach the final flow of 1 mL/min and maximum eluent composition of B at 65%. These parameters were held for 0.5 min, and a 0.5 min linear gradient was used to reach the initial flow rate and eluent composition. This was followed by a 1 min equilibrating part. Data acquisition and processing were performed using Analyst TF software version 1.7.1 (AB Sciex Instruments, CA, USA).

1.2.6.4 Digestion and LC-MS/MS measurements of DdlB, cHDAC4 and FBW7 samples on TripleTOF 5600+ instrument

After the labelling was completed, 50 µL of the sample and 10 µL 0.2% (w/v) RapiGest SF (Waters, Milford, USA) solution buffered with 50 mM ammonium bicarbonate were mixed (pH=7.8), and 6 µL of 45 mM dithiotreitol (DTT) in 100 mM NH_4HCO_3 were added and kept at 37.5 °C for 30 min. After cooling the sample to room temperature, 7 µL of 100 mM iodoacetamide in 100 mM NH_4HCO_3 was added and placed in the dark at room temperature for 30 min. The reduced and alkylated protein was then digested by 6.7 µL (1 mg/mL) trypsin (the enzyme-to-protein ratio was 1:10) (Sigma, St Louis, MO, USA). The sample was incubated at 37 °C overnight. To degrade the surfactant, 6 µL of formic acid (500 mM) solution was added to the digested protein sample to obtain the final 40 mM concentration (pH ≈ 2), and was incubated at 37 °C for 30 min. For LC-MS analysis, the acid-treated sample was centrifuged for 5 min at 13,000 rpm, and the supernatant was pipetted into a microvial.

To get more precise information on the structure, samples were further analyzed by a Triple TOF 5600+ hybrid Quadrupole-TOF LC/MS/MS system (Sciex, MA, USA) equipped with a DuoSpray IonSource coupled with a Shimadzu Prominence LC20 UFLC (Shimadzu, Japan) system consisting of a quaternary pump, an autosampler and a thermostated column compartment.

Data acquisition and processing were performed using Analyst TF software version 1.7.1 (AB Sciex Instruments, CA, USA). Chromatographic separation was achieved on the Discovery® BIO Wide Pore C-18-5 (250 mm × 2.1mm, 5 µm, 300 Å) HPLC column. The sample was eluted in gradient elution mode using solvent A (0.1% formic acid in water) and solvent B (0.1% formic acid in ACN). The initial condition was 5% B for 7 min, followed by a linear gradient to 90% B by 48 min, from 55 to 63 min 90% B was retained; and from 63 to 65 min, back to the initial condition with 5 % eluent B and retained for 10 min. Flow rate was set to 0.2 ml/min. The column temperature was 40 °C and the injection volume was 15 µl. Nitrogen was used as the nebulizer gas (GS1), heater gas (GS2), and curtain gas with the optimum values set at 35, 35 and 35 (arbitrary units), respectively. The source temperature was 350 °C and the spray voltage was set to 5000 V.

Advanced **Information Dependent Acquisition (IDA**) mode was used on the TripleTOF 5600+ system to obtain MS/MS spectra on the 8 most abundant parent ions present in the TOF survey scan. In the **IDA** LC-MS/MS experiment, the mass spectra and tandem mass spectra were recorded in "high-sensitivity" mode with a resolution of ~35,000 full-width half-maximum.

In the first period (positive TOF MS mode) the data were acquired in the mass range of m/z=300 to 2500, with 0.1 s accumulation time. Declustering potential value was set to 60 V. The intensity threshold for precursor ion selection in TOF survey scan mode was 1000 cps. In

the MS2 experiment (Product Ion scan mode): the mass range was m/z=50 to 3000, with an accumulation time of 0.1 s.

Initial data was handled with PeakView software (version 2.2, Sciex) and Sciex OS (version 3.4.5.828). Acquisition files were converted for open-source applications by MSConvert (version 3.0.25071, ProteoWizard). Bottom-up proteomics search was executed by SearchGUI (version 4.3.15, CompOmics) with four algorithms (Comet, Tide, MetaMorpheus, and Sage). Results were analyzed by PeptideShaker (version 3.0.11, CompOmics). Novel self-made software, developed with the Python programming language (version 3.11.0), pyOpenMS (version 3.3.0), and with the help of Spyder Integrated Development Environment (version 5.5.0), was used for the final evaluation.

1.2.6.5 Digestion and LC-MS/MS measurements of KRAS^{G12D} samples on Waters instrument

Mass spectrometric experiments for KRAS^{G12D} were performed on a high-resolution hybrid quadrupole-time-of-flight mass spectrometer (Waters Select Series Cyclic IMS, Waters Corp., Wilmslow, U.K.). The mass spectrometer operated in positive V mode. Leucine enkephalin was used as Lock Mass standard. Chromatographic separations were performed on a Waters Acquity I-Class UPLC system, coupled directly to the mass spectrometer. Modification sites were determined by RPLC-MS/MS peptide mapping after proteolysis using trypsin. Briefly, the protein was enzymatically digested after buffer exchange using Amicon Ultra-0.5 mL Centrifugal Filter units (10 kDa, Merck Millipore). Protein samples were reduced by dithiothreitol at 37 °C for 30 min. Tryptic cleavage was performed in 50 mM ammonium bicarbonate solution (pH 7.8) with sequencing-grade trypsin (Promega Corporation, Madison, USA) using 1:20 enzyme:protein ratio at 37°C for 12 hours. Digestion was stopped by adding formic acid in a final concentration of 0.2% (V/V).

Gradient elution was performed on a Waters Acquity Peptide BEH C18 UPLC column (2.1x150 mm, 1.7 µm) under the following parameters: mobile phase "A": 0.1% formic acid in water, mobile phase "B": 0.1% formic acid in acetonitrile; flow rate: 300 µL/min; column temperature: 60 °C; gradient: 2 min: 2% B, 20 min: 55% B, 20.5 min: 90% B. MS^E experiments were performed using collision voltage ramping under the following parameters: *m/z* 50-2000, scan time: 0.3 s, single Lock Mass: leucine enkephalin; low energy: 6 V, high energy: ramping 19-45 V.

BiopharmaLynx 1.3.5 software (Waters Corp., Wilmslow, U.K.) was used to for data analysis.

1.2.7 Computational methods

1.2.7.1 DdlB

Protein structure with PDB ID 4C5A[8] was prepared with Maestro's Protein Preparation Workflow[11] with default settings except that only the positions of H-atoms were minimized.

Ligands were prepared using LigPrep[11] (Schrödinger Release 2025-2: LigPrep, Schrödinger, LLC, New York, NY, 2025.) using default settings. Covalent docking was performed using CovDock[12] (Pose Prediction docking mode), targeting residue K144. Binding site mapping was performed on the same PDB structure with the FTMap webserver[13,14] (default settings), as well as SiteMap [15,16](full protein mapping, and reporting max. 50 sites). The detected binding sites were checked against the location of labeled lysines and inspected visually to infer structural or functional significance.

1.2.7.2 HDAC4

Protein structure with PDB ID 2VQJ[17] were prepared with Maestro's Protein Preparation Workflow,[11] using default method, while ligands were prepared using LigPrep[11] (Schrödinger Release 2025-2: LigPrep, Schrödinger, LLC, New York, NY, 2025.) with default method. Dockings were performed using Glide[18,19] and CovDock.[12] For the docking runs the grid was defined using the targeted Lys as centroid of the box. Non-covalent docking was performed using Glide SP, with default settings, while covalent docking was performed using CovDock in Pose Prediction mode. Binding site mapping was performed on the same PDB structure with the FTMap webserver[13,14] (default settings), as well as SiteMap [15,16](full protein mapping, and reporting max. 50 sites). The detected binding sites were checked against the location of labeled lysines and inspected visually to infer structural or functional significance.

1.2.7.3 STAT3

Protein structure with PDB ID 6QHD[4] was prepared with Maestro's Protein Preparation Workflow[11] at a pH of 7.4, while ligands were prepared using LigPrep[11] (Schrödinger Release 2025-2: LigPrep, Schrödinger, LLC, New York, NY, 2025.) at a pH range of 7.4±1.0. Covalent docking was performed using CovDock[12] (Pose Prediction docking mode), targeting residue Lys591 located at the SH2 domain. Binding site mapping was performed on the same PDB structure with the FTMap webserver[13,14] (default settings), as well as SiteMap [15,16](full protein mapping, and reporting max. 50 sites). The detected binding sites were checked against the location of labeled lysines and inspected visually to infer structural or functional significance.

1.2.7.4 KRAS^{G12D}

Protein structure with PDB ID 6OIM[5] were prepared with Maestro's Protein Preparation Workflow,[11] using default method, while ligands were prepared using LigPrep (Schrödinger Release 2025-2: LigPrep, Schrödinger, LLC, New York, NY, 2025.) with default method. Dockings were performed using Glide[18,19] and CovDock.[12] For the docking runs the grid was defined using the targeted Lys as centroid of the box. Noncovalent docking was performed using Glide SP, with default settings, while covalent docking was performed using CovDock in Pose Prediction mode. Binding site mapping was performed on the same PDB structure with the FTMap webserver[13,14] (default settings), as well as SiteMap [15,16](full protein mapping, and

reporting max. 50 sites). The detected binding sites were checked against the location of labeled lysines and inspected visually to infer structural or functional significance.

1.2.7.5 FBW7

Protein structure with PDB ID 2OVP[6] was prepared with Maestro's Protein Preparation Workflow[11] at a pH of 7.4, while ligands were prepared using LigPrep[11] (Schrödinger Release 2025-2: LigPrep, Schrödinger, LLC, New York, NY, 2025.) at a pH range of 7.4±1.0. Covalent docking was performed using CovDock[12](Pose Prediction docking mode), targeting either residue Lys326, Lys343, Lys404 or Lys412. Binding site mapping was performed on the same PDB structure with the FTMap webserver[13,14] (default settings), as well as SiteMap [15,16](full protein mapping, and reporting max. 50 sites). The detected binding sites were checked against the location of labeled lysines and inspected visually to infer structural or functional significance. Binding site mapping was performed on the same PDB structure with the FTMap webserver[13,14] (default settings), as well as SiteMap [15,16](full protein mapping, and reporting max. 50 sites). The detected binding sites were checked against the location of labeled lysines and inspected visually to infer structural or functional significance.

1.3 Results

1.3.1 Dose-response curves for DdlB

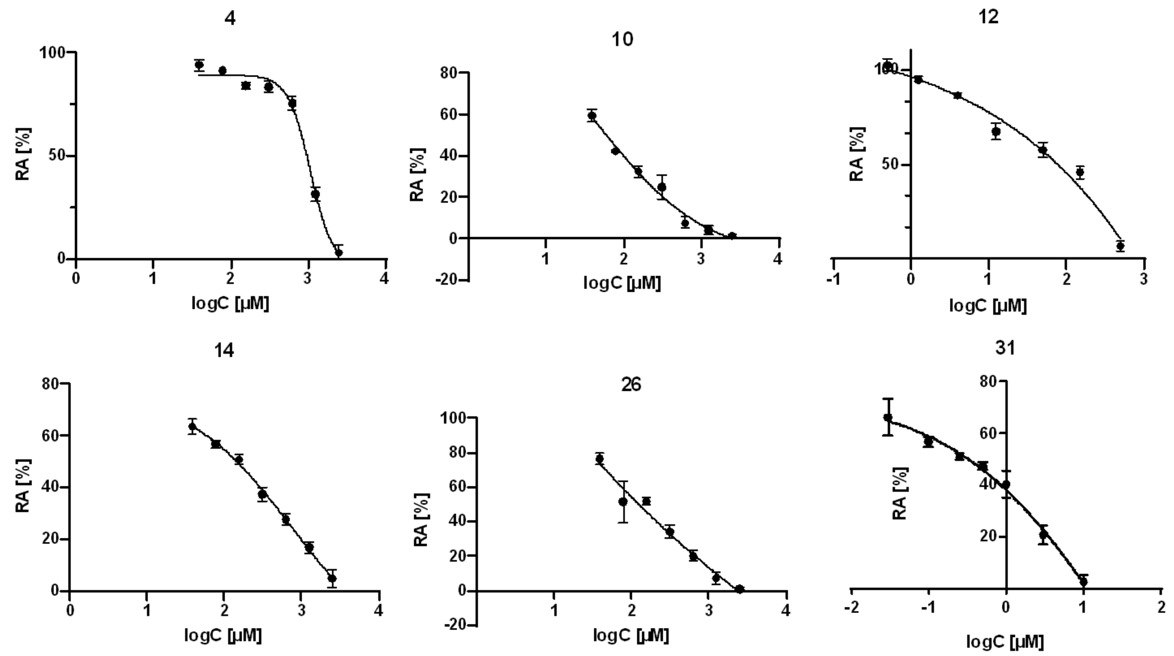

Figure S11. Dose-response curves against DdlB measured in the end-point malachite green assay by detecting the orthophosphate formed during the enzymatic reaction. IC_{50} values were determined for the single-point screen hits by measuring the residual activities (RA) at seven different compound concentrations after 30 min pre-incubation at 37 °C. Error bars represent standard deviation (SD) from technical duplicates.

4: IC_{50}=1160 ± 77 μM; R^2=0.9908

10: IC_{50}=96 ± 7 μM; R^2=0.9924

12: IC_{50}=102 ± 9 μM; R^2=0.9960

14: IC_{50}=531 ± 28 μM; R^2=0.9982

26: IC_{50}=309 ± 16 μM; R^2=0.9960

31: IC_{50}=4 ± 0.8 μM; R^2=0.9923)

1.3.2 Intact mass spectrometry results of DdlB

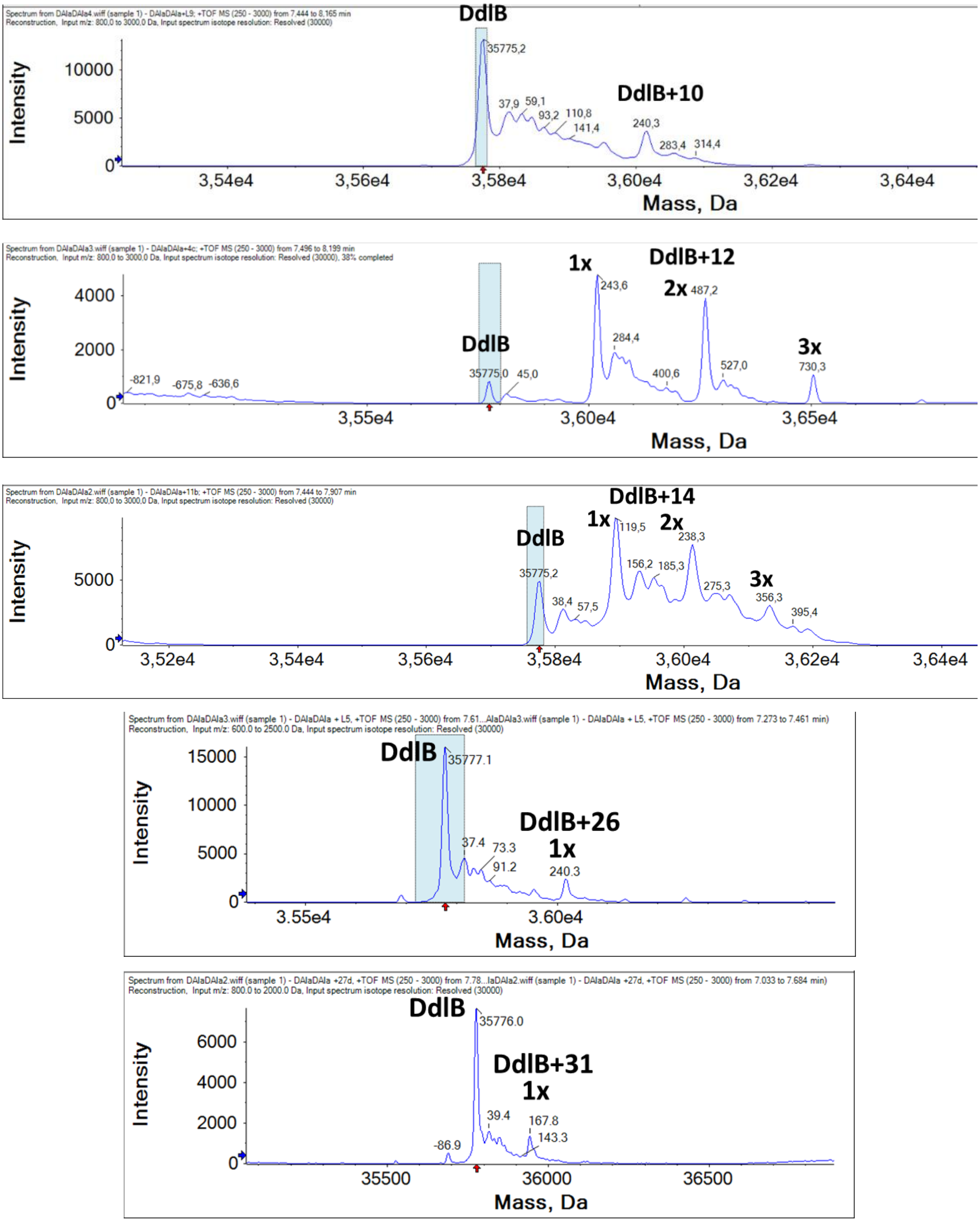

Figure S12. Deconvoluted mass spectrum of **reference** DdlB and **10** (+240 Da), **12** (+243 Da), **14** (+119 Da), **26** (+240 Da) and **31** (+166 Da) modified DdlB.

1.3.3 Digestion results of DdlB

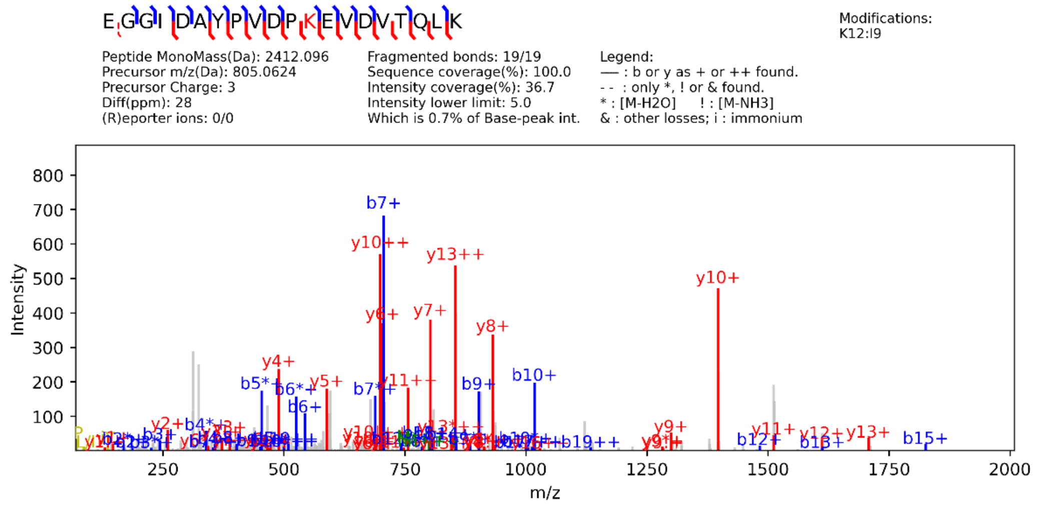

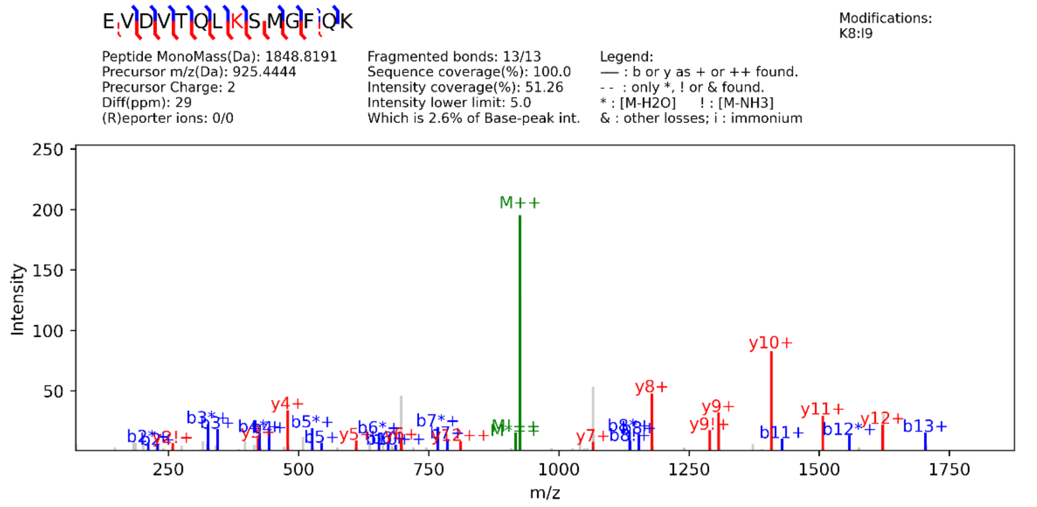

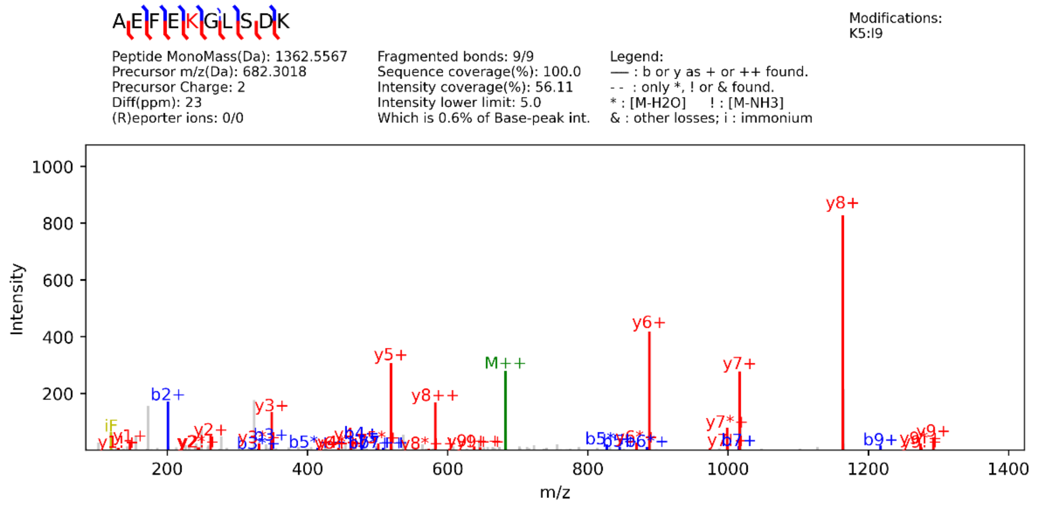

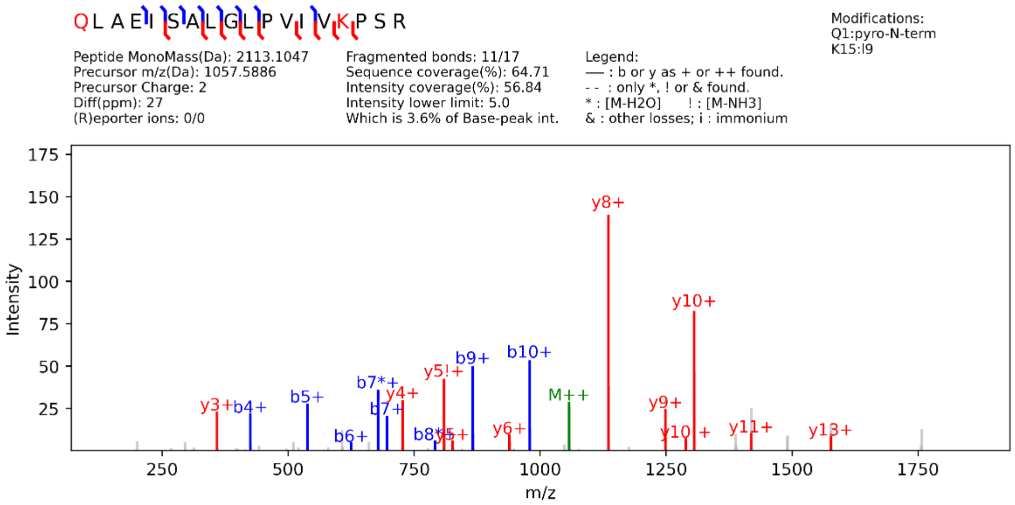

Sequence	Position start	Position end	Missed Cleavages	RT (min)
EGGIDAYPVDPKEVDVTQLK	32	51	1	33.07
EVDVTQLKSMGFQK	44	57	1	33.94
AEFEKGLSDK	120	129	1	31.36
QLAEISALGLPVIVKPSR	130	147	1	38.25

Figure S13. MS/MS spectra and analytical data of **10** modified DdlB enzyme peptides detected in the peptide mapping analysis using tryptic digestion. Labelled amino acids are **K43**, **K51**, **K124**, and **K144**, respectively.

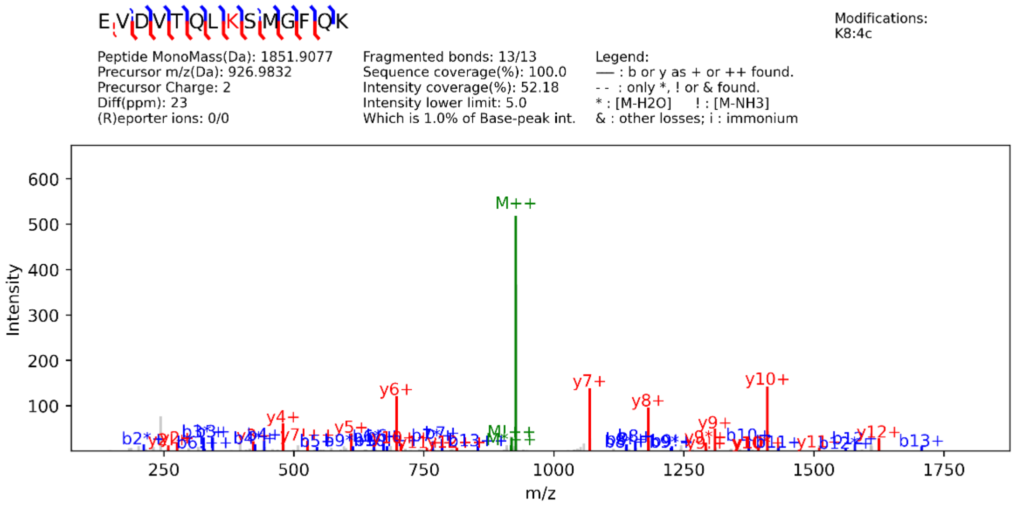

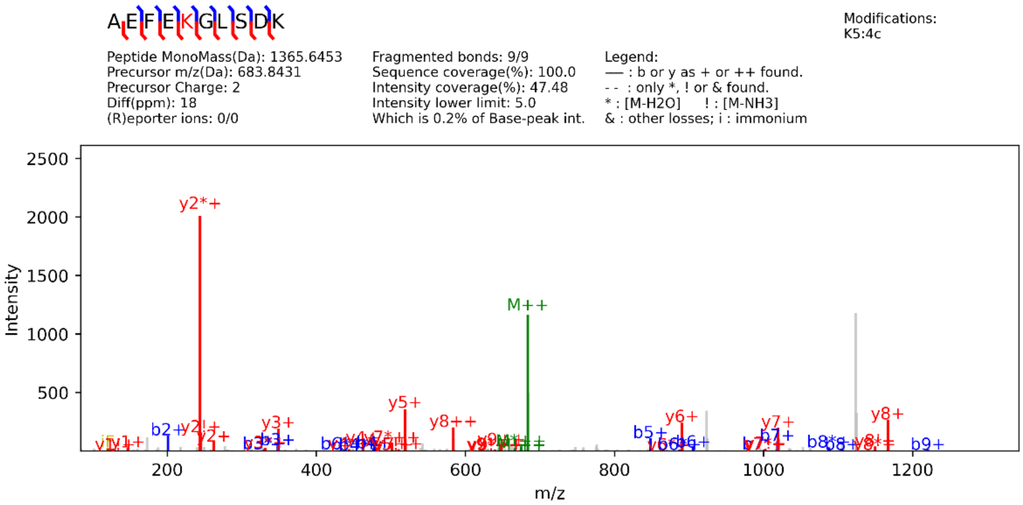

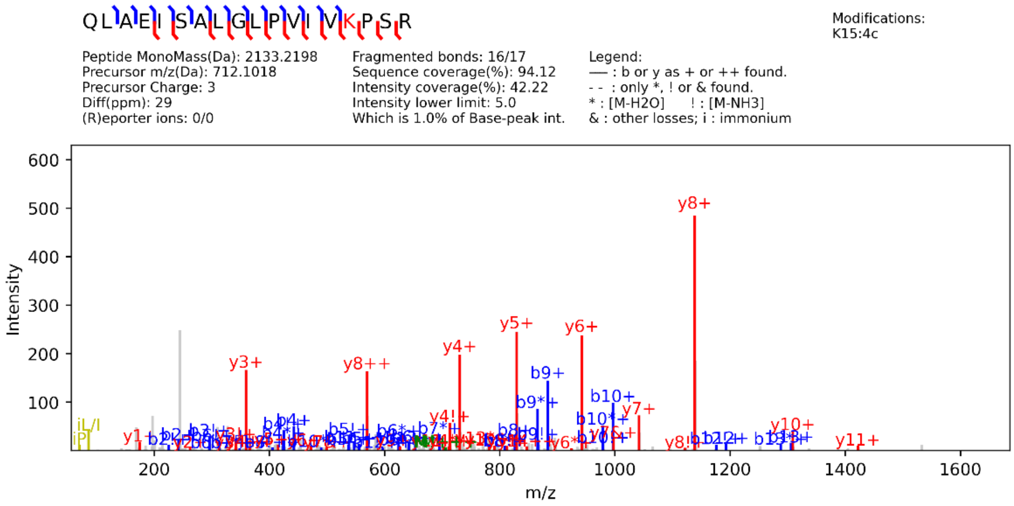

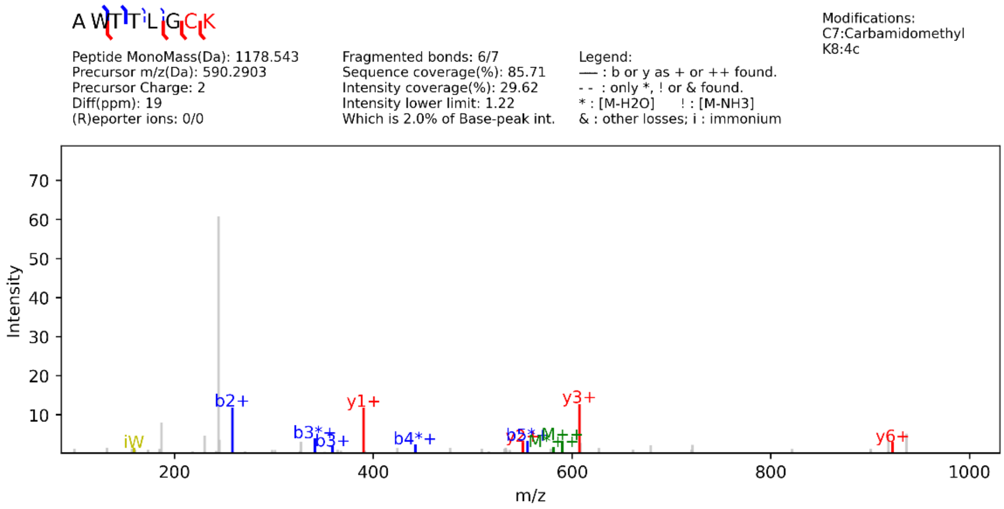

Sequence	Position start	Position end	Missed Cleavages	RT (min)
EVDVTQLKSMGFQK	44	57	1	32.87

AEFEKGLSDK	120	129	1	30.24
QLAEISALGLPVIVKPSR	130	147	1	35.13
AWTTLGCK	244	251	0	33.57

Figure S14. MS/MS spectra and analytical data of **12** modified DdlB enzyme peptides detected in the peptide mapping analysis using tryptic digestion. Labelled amino acids are **K51**, **K124**, **K144**, and **K251**, respectively.

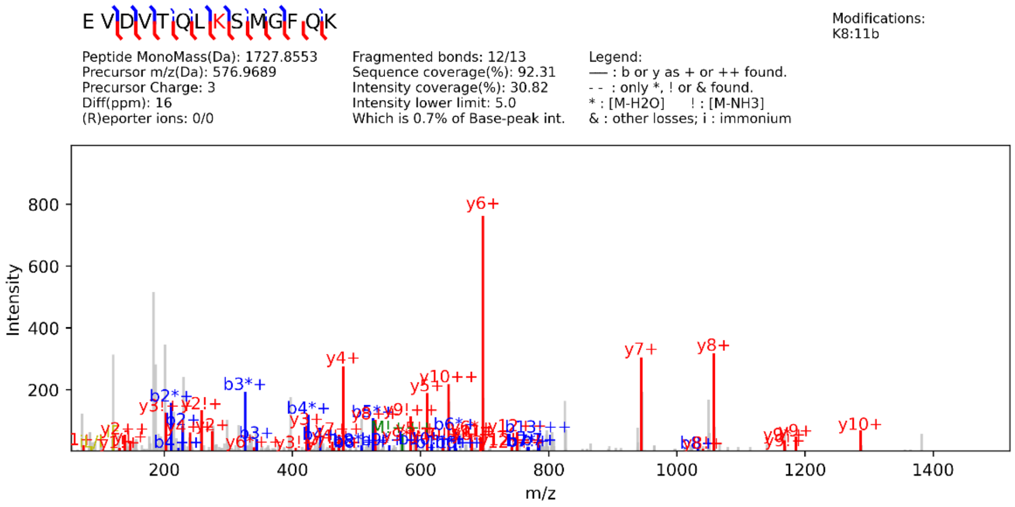

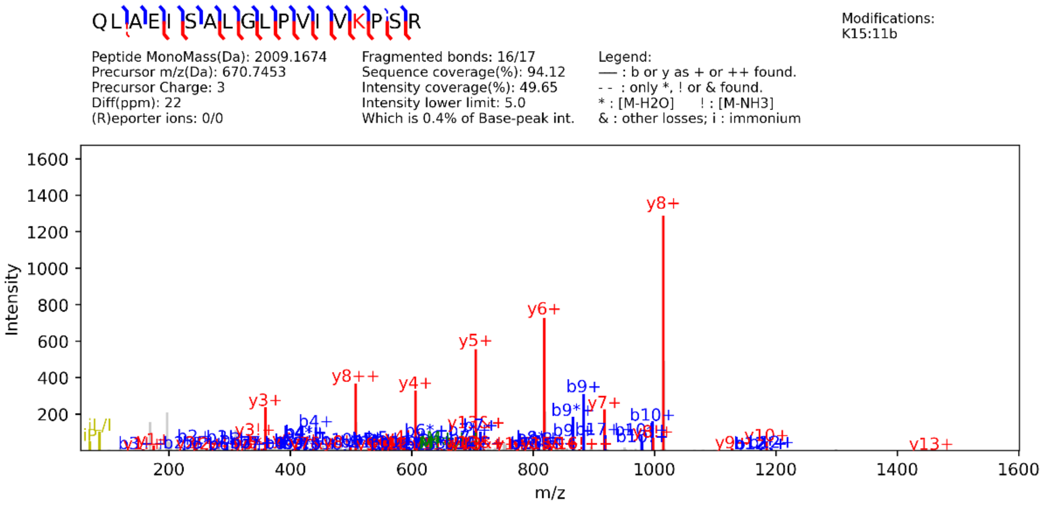

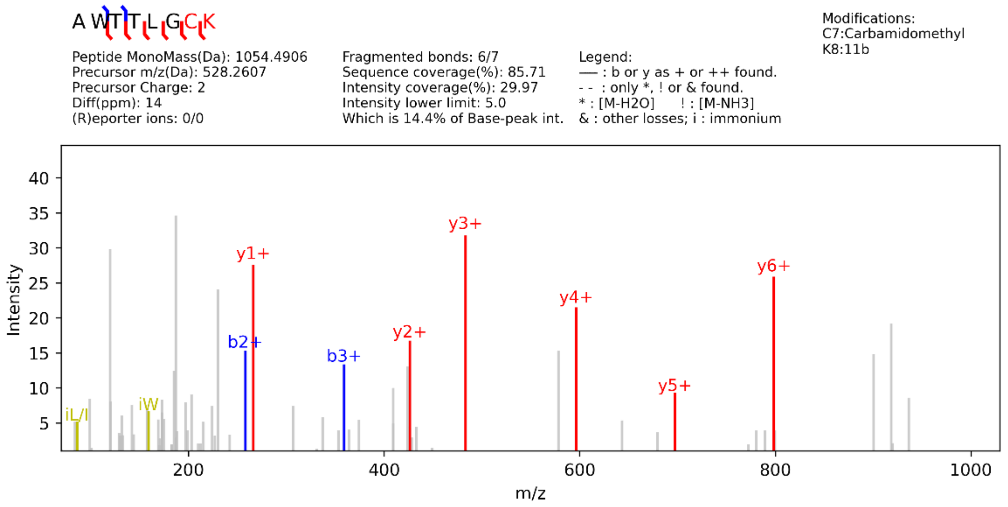

Sequence	Position start	Position end	Missed Cleavages	RT (min)
EVDVTQLKSMGFQK	44	57	1	28.27
QLAEISALGLPVIVKPSR	130	147	1	33.66
AWTTLGCK	244	251	0	27.22

Figure S15. MS/MS spectra and analytical data of **14** modified DdlB enzyme peptides detected in the peptide mapping analysis using tryptic digestion. Labelled amino acids are **K51**, **K144**, **K251**, respectively.

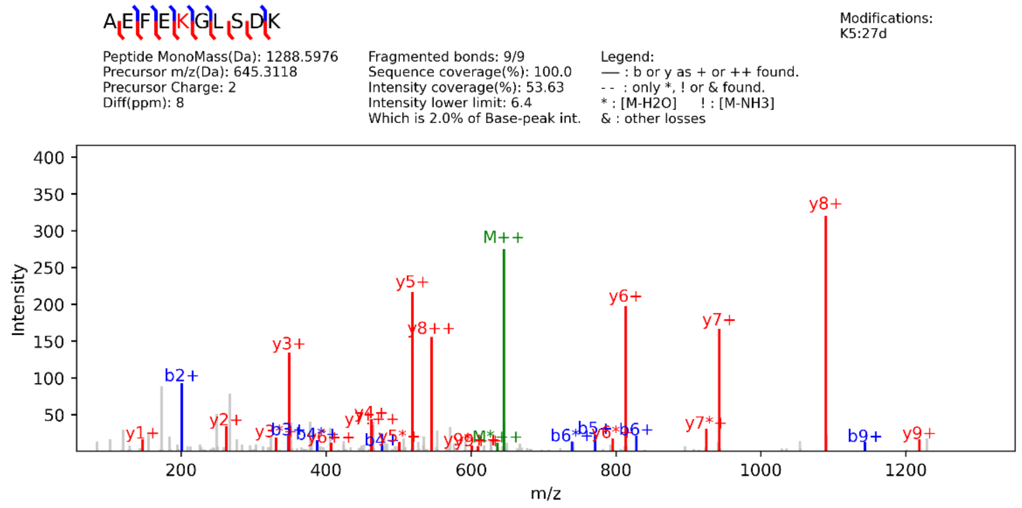

Figure S16. MS/MS spectra and analytical data of a **31** modified DdlB enzyme peptide detected in the peptide mapping analysis using tryptic digestion. Labelled amino acid is **K124**.

1.3.4 Dose-response curves for STAT3

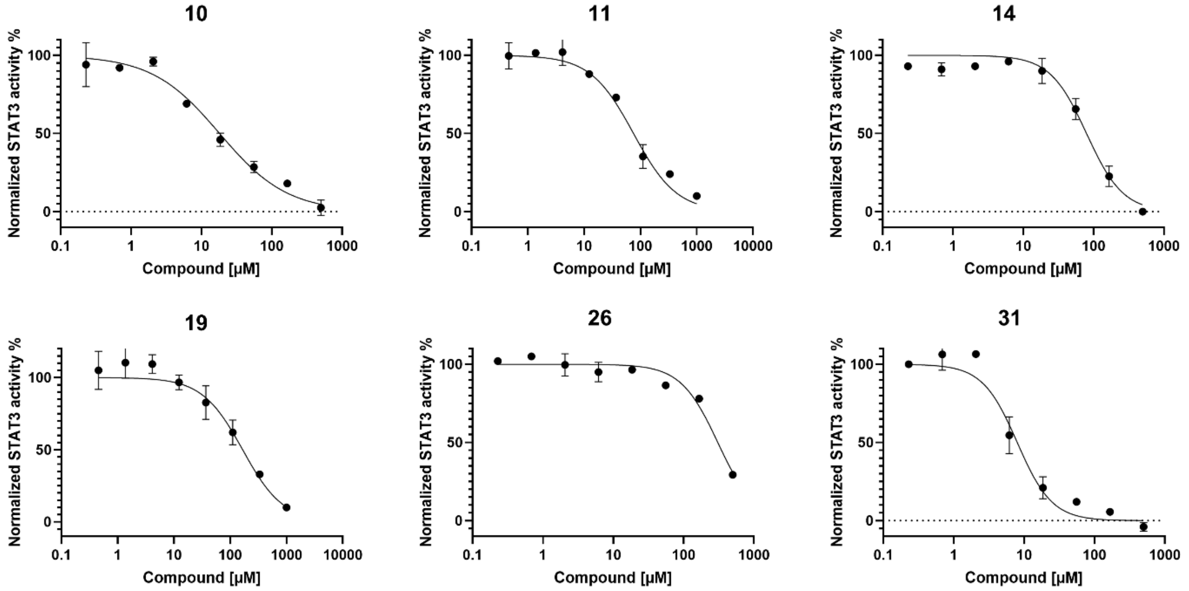

Figure S17. Fluorescence polarization assay results after pre-incubation for 1h at 37 °C, after which fragment binding was assessed by displacement of a potent fluorescein-labeled phosphotyrosine peptide (FAM-GpYLPQTV) from STAT3. IC_{50}s are presented with 95% confidence intervals, error bars represent standard deviation (SD) from technical triplicates.

10: IC_{50}=18.2±4.9 µM; R^2=0.9696

11: IC_{50}=75.5±17.5 µM; R^2=0.9708

14: IC_{50}=79.4±11.9 µM; R^2=0.9688

19: IC_{50}=166.3± 49.3 µM; R^2=0.8819

26: IC_{50}=306.4±44.5 µM; R^2=0.9590

31: IC_{50}=8.0±1.7 µM; R^2=0.9537

1.3.5 Intact mass spectrometry results of STAT3

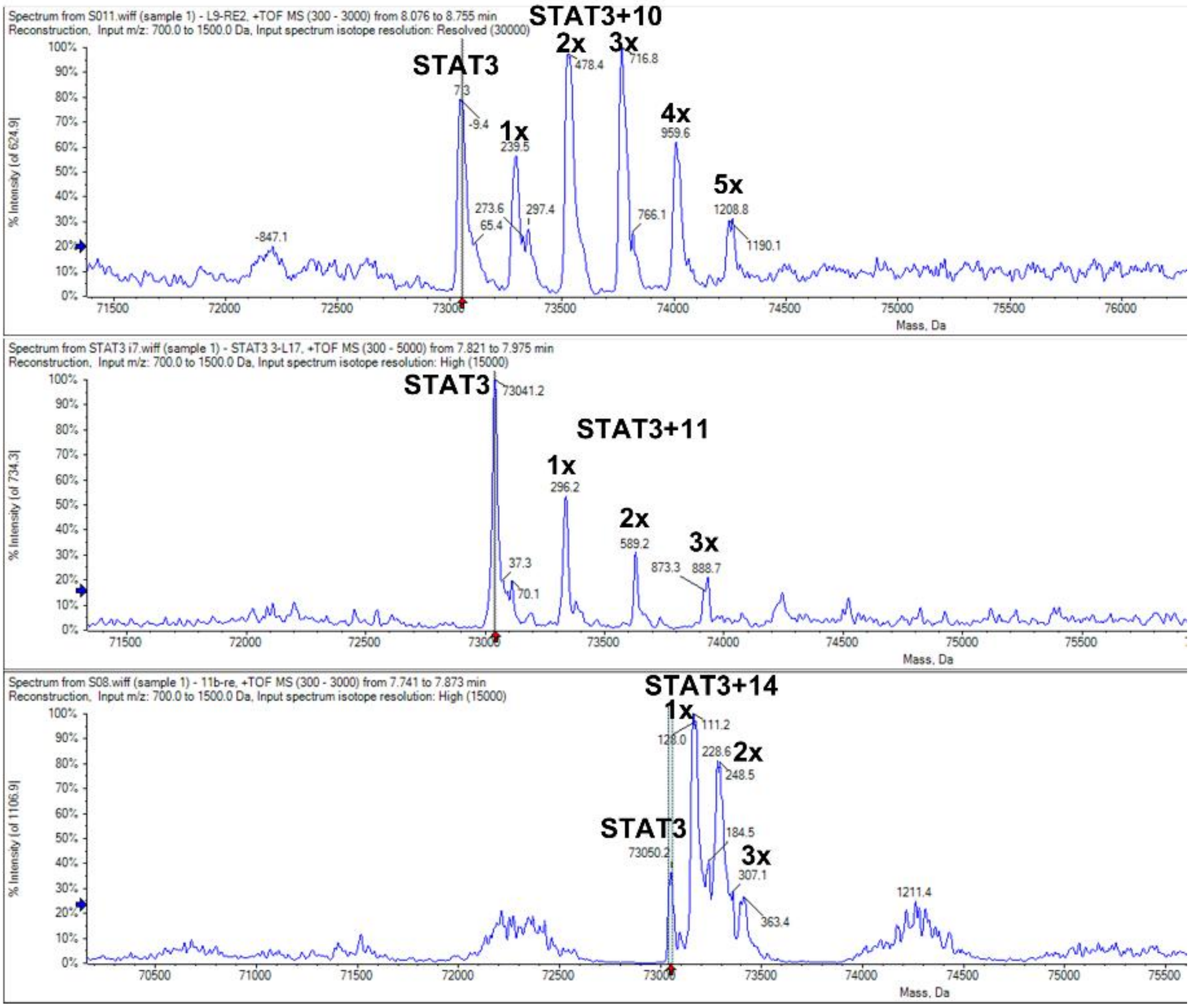

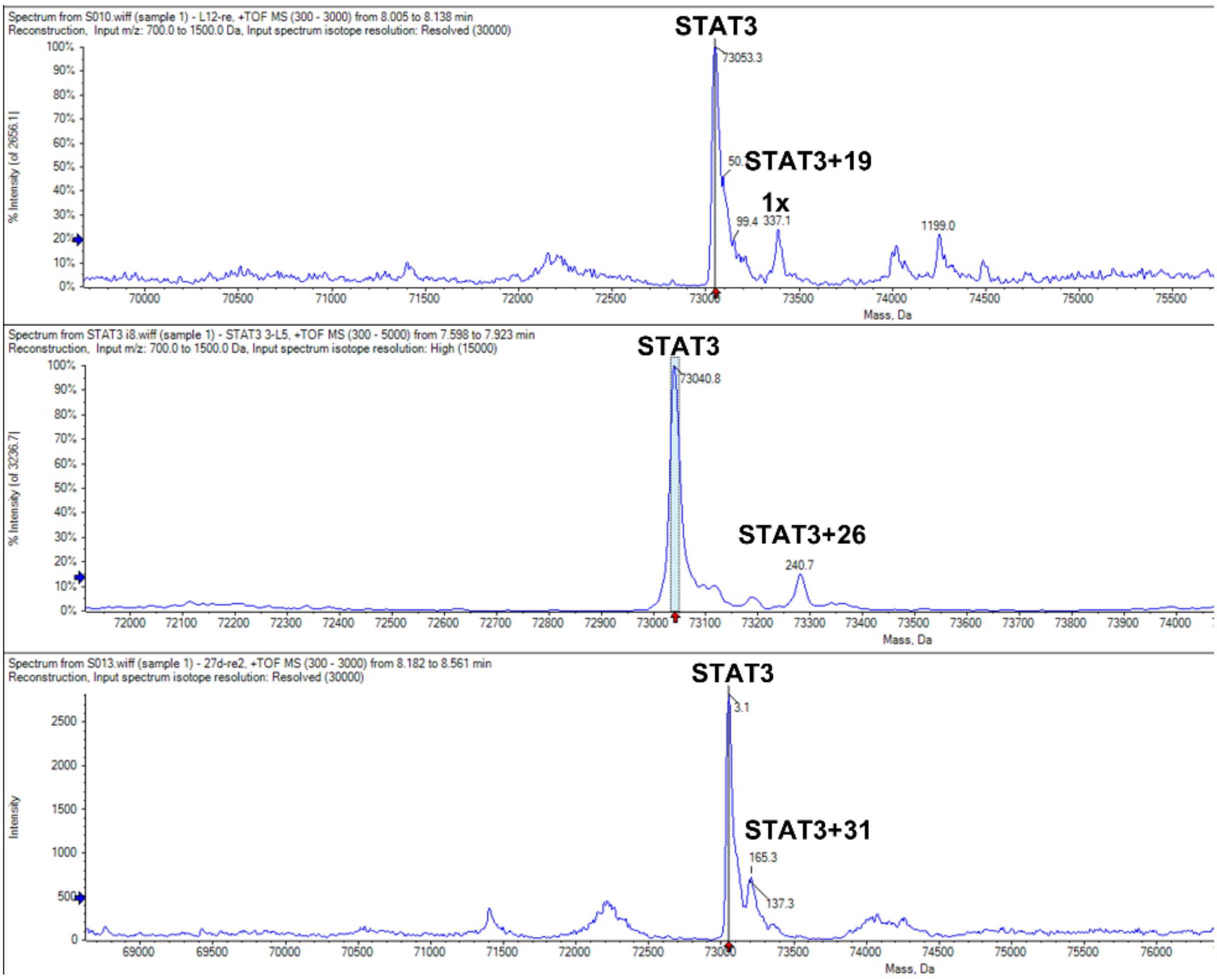

Figure S18. Deconvoluted mass spectrum of **STAT3** labeled by **10** (+240 Da), **11** (+296 Da), **14** (+119 Da),**19** (+340 Da) **26** (+240 Da) and **31** (+166 Da), respectively.

1.3.6 Dose-response curves for cHDAC4

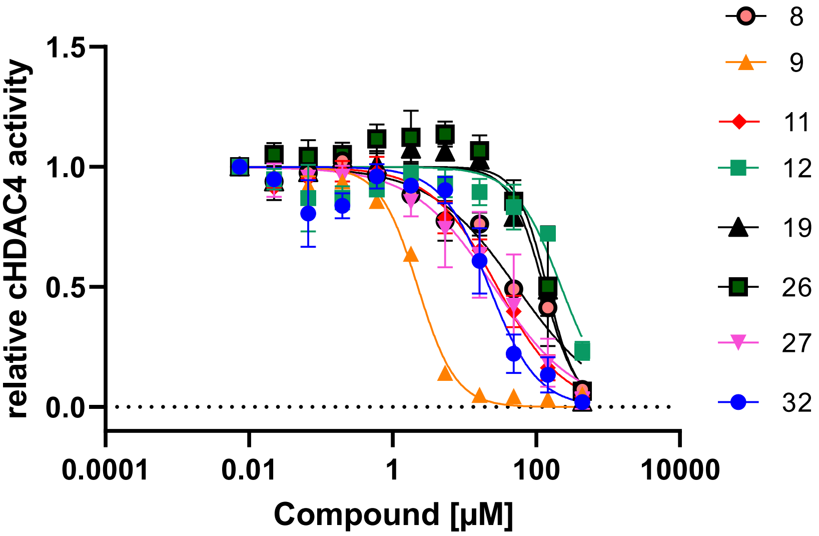

Figure S19. Dose-response curves against cHDAC4. IC_{50} values were determined for the single-point screen hits by measuring the residual activities after 1 h pre-incubation at 30 °C. Relative cHDAC4 activity was determined following control-based normalization. Error bars represent standard deviation (SD) from technical triplicates.

8: IC_{50}=52.9±11.4 μM; R^2=0.9503

9: IC_{50}= 2.2±0.2 μM; R^2=0.9884

11: IC_{50}=28.8±4.3 μM; R^2=0.9784

12: IC_{50}= 223.3±51.3 μM; R^2=0.8017

19: IC_{50}=126.9±16.4 μM; R^2= 0.9643

26: IC_{50}= 141.5±30.4 μM; R^2=0.8875

27: IC_{50}= 25.8±7.6 μM; R^2=0.9187

32 IC_{50}= 22.2±5.4 μM; R^2=0.9178

1.3.7 Intact mass spectrometry results of HDAC4

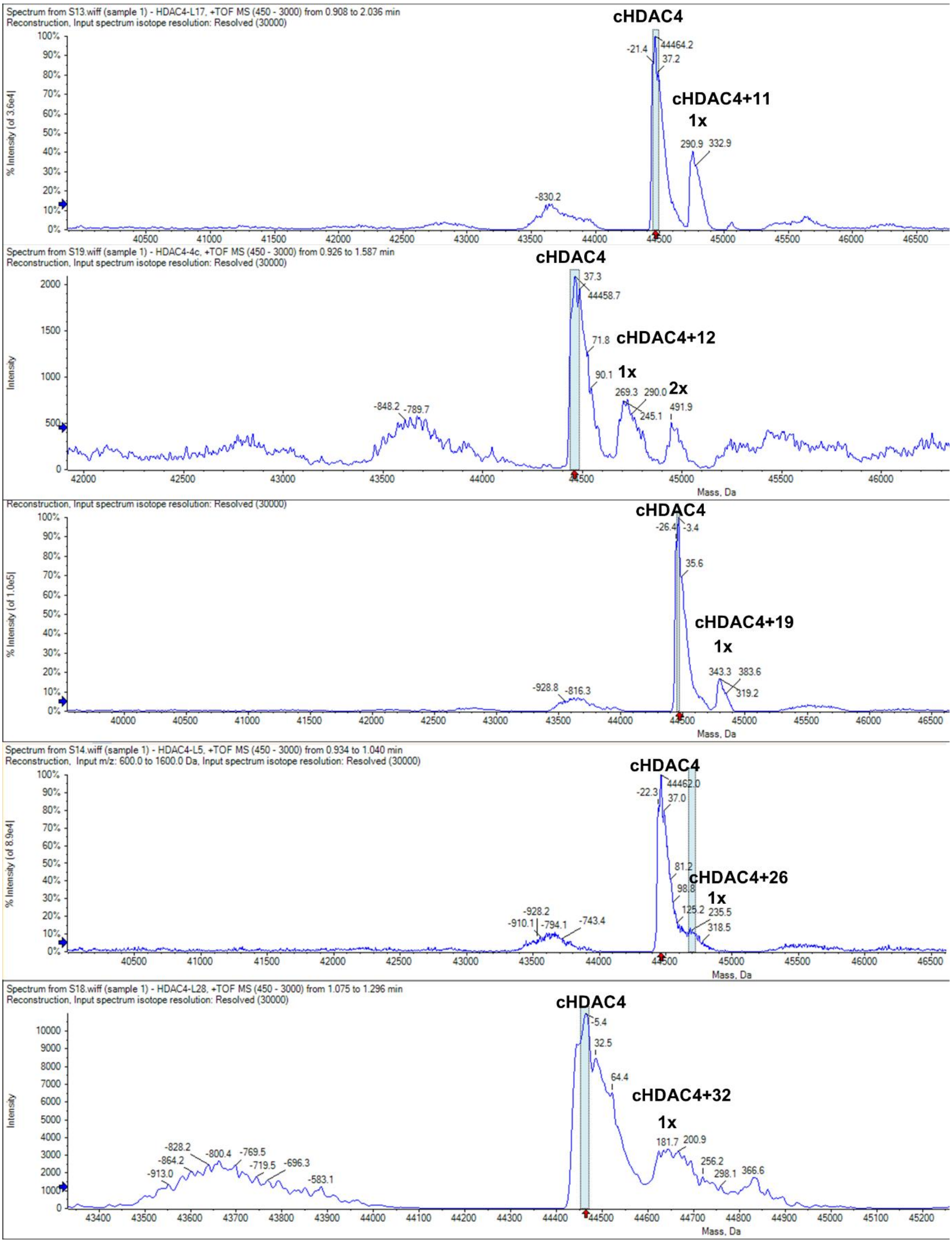

Figure S20. Deconvoluted mass spectrum of **cHDAC4** labelled by **11** (+296 Da), **12** (+243 Da), **19** (+340 Da), **26** (+240 Da) and **32** (+180 Da), respectively.

1.3.8 Digestion results of HDAC4

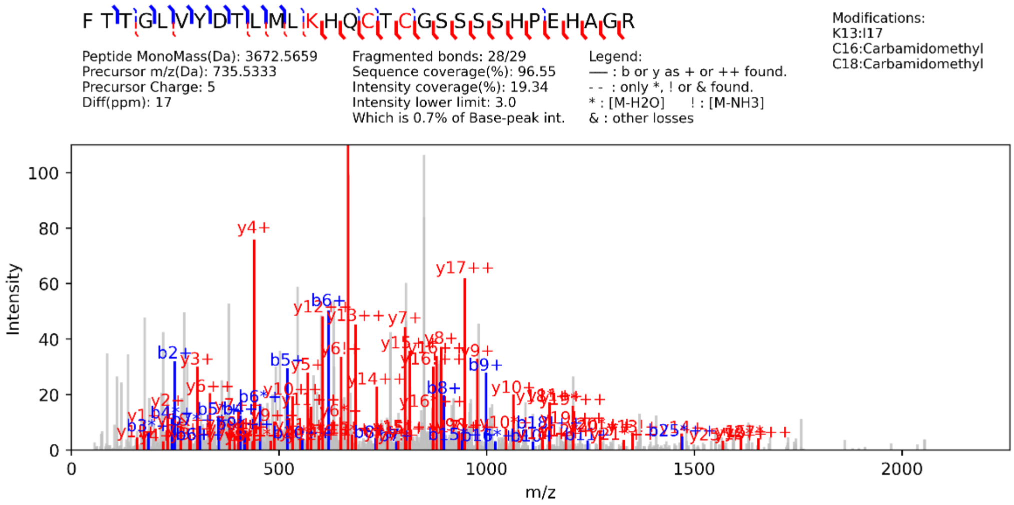

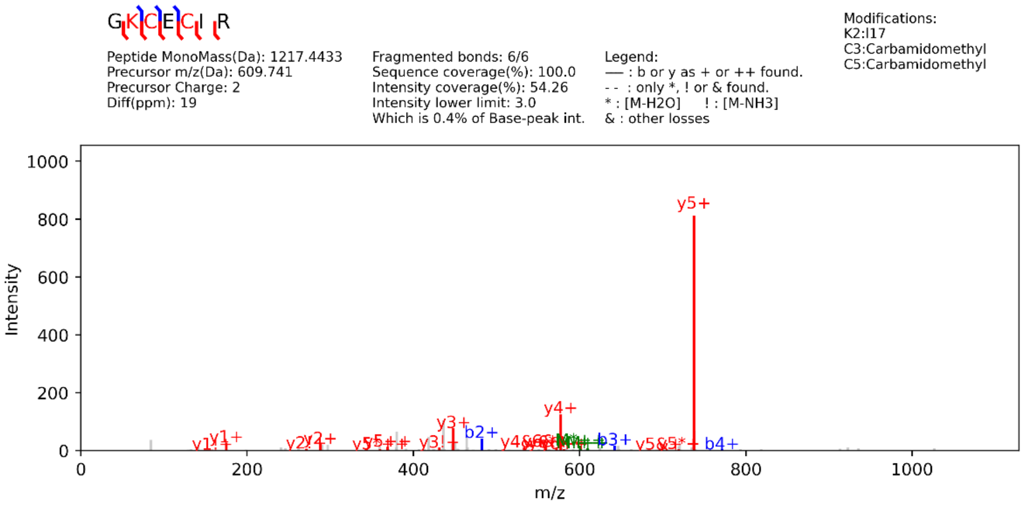

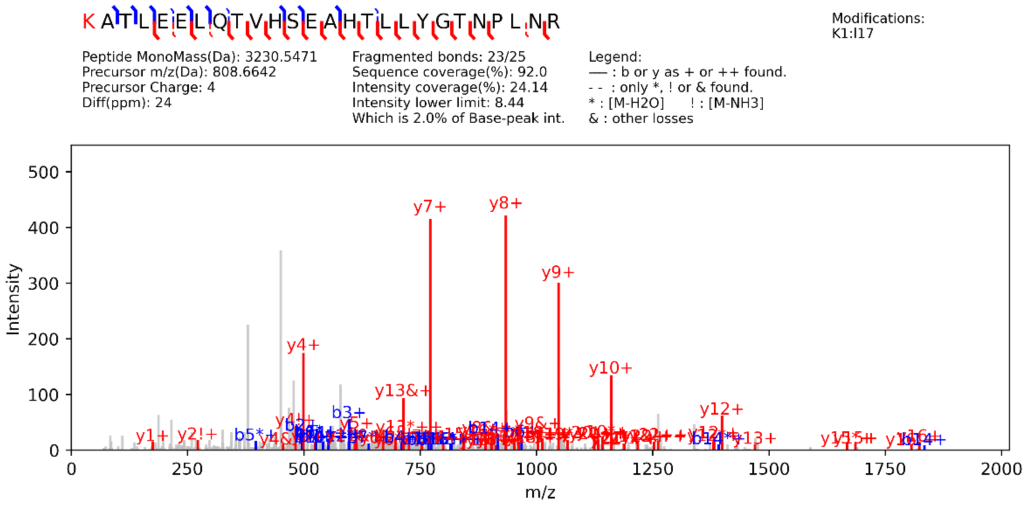

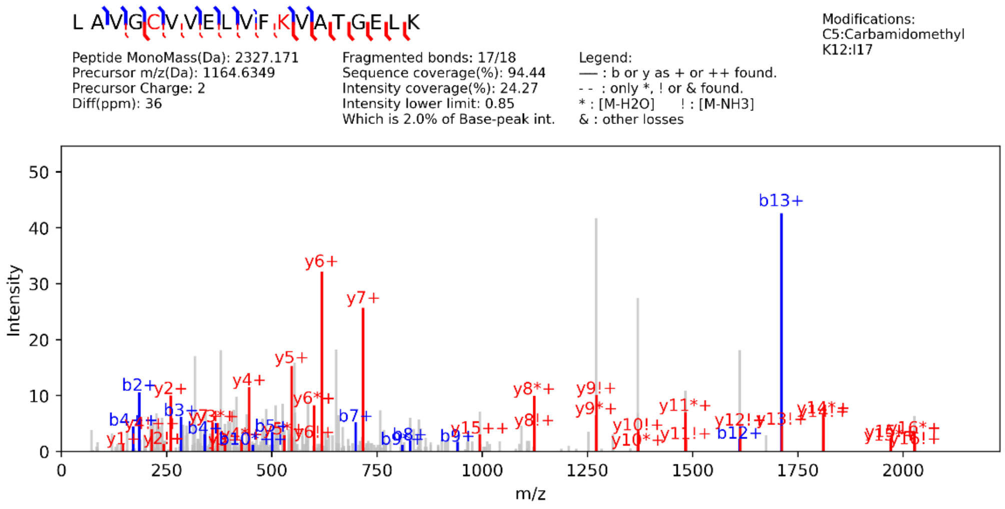

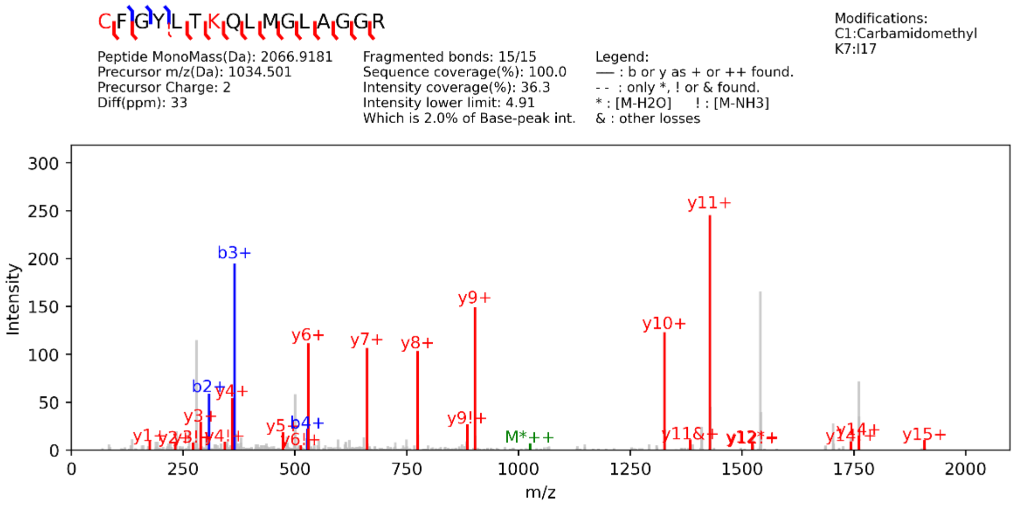

Sequence	Position start	Position end	Missed Cleavages	RT (min)
FTTGLVYDTLMLKHQCTCGSSSSHPEHAGR	8	38	1	33.28
GKCECIR	52	58	1	30.82
KATLEELQTVHSEAHTLLYGTNPLNR	61	86	1	32.25
LAVGCVVELVFKVATGELK	129	147	1	43.02
CFGYLTKQLMGLAGGR	308	323	1	35.41

Figure S21. MS/MS spectra and analytical data of **11** modified **cHDAC4** enzyme peptides detected in the peptide mapping analysis using tryptic digestion. Labelled amino acids are **K20**, **K53**, **K61**, **K140** and **K314**, respectively.

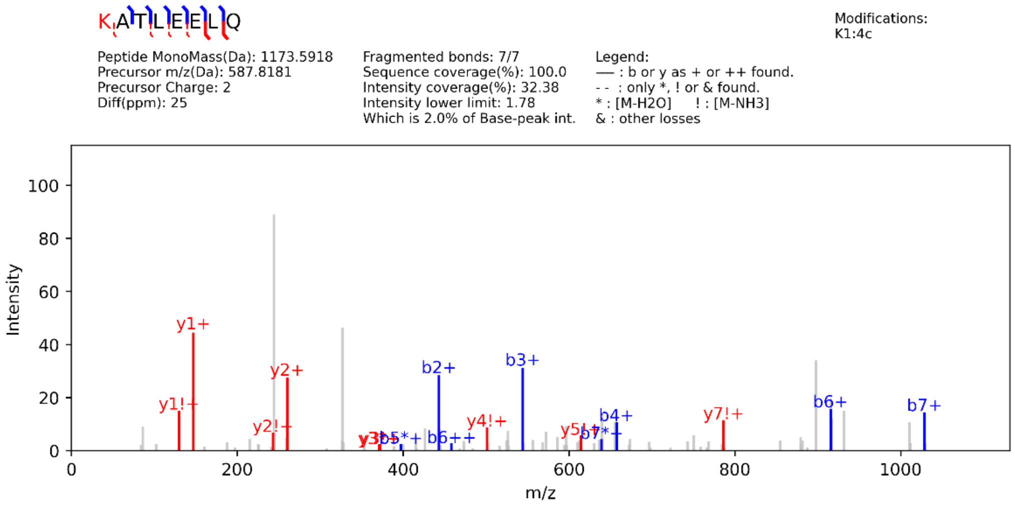

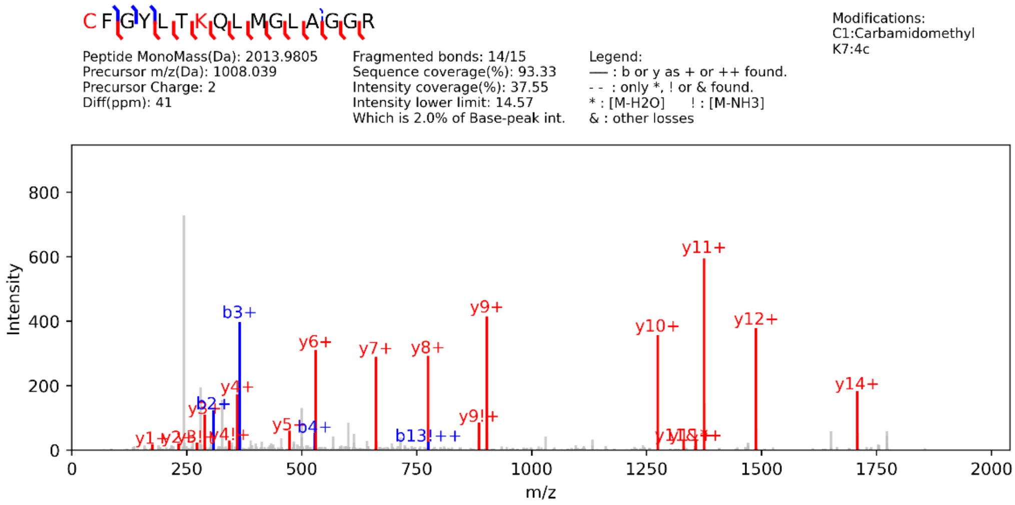

Sequence	Position start	Position end	Missed Cleavages	RT (min)
KATLEELQ	61	68	1	31.92
CFGYLTKQLMGLAGGR	308	323	1	34.72

Figure S22. MS/MS spectra and analytical data of **12** modified **cHDAC4** enzyme peptides detected in the peptide mapping analysis using tryptic digestion. Labelled amino acids are **K61** and **K314**, respectively.

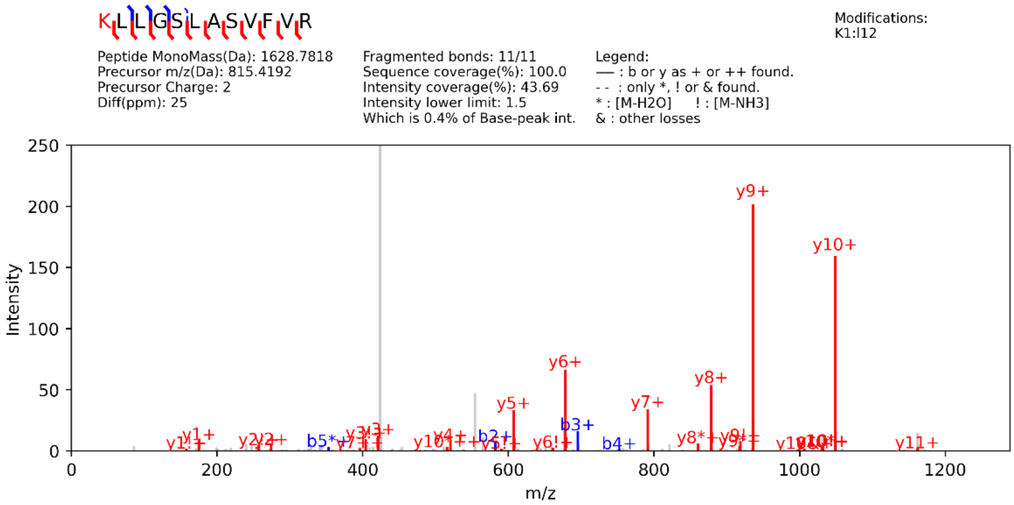

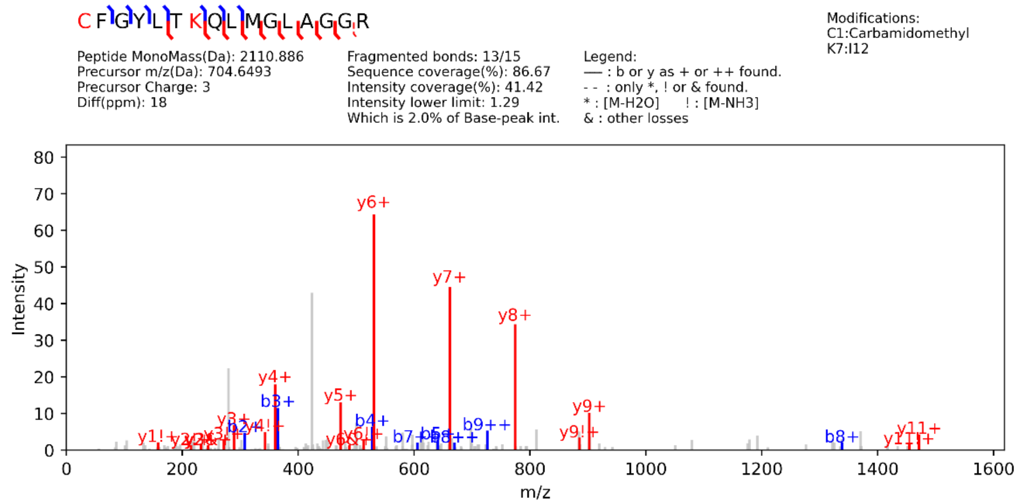

Sequence	Position start	Position end	Missed Cleavages	RT (min)
KLLGSLASVFVR	93	104	1	40.31
CFGYLTKQLMGLAGGR	308	323	1	37.73

Figure S23. MS/MS spectra and analytical data of **19** modified **cHDAC4** enzyme peptides detected in the peptide mapping analysis using tryptic digestion. Labelled amino acids are **K93** and **K314**, respectively.

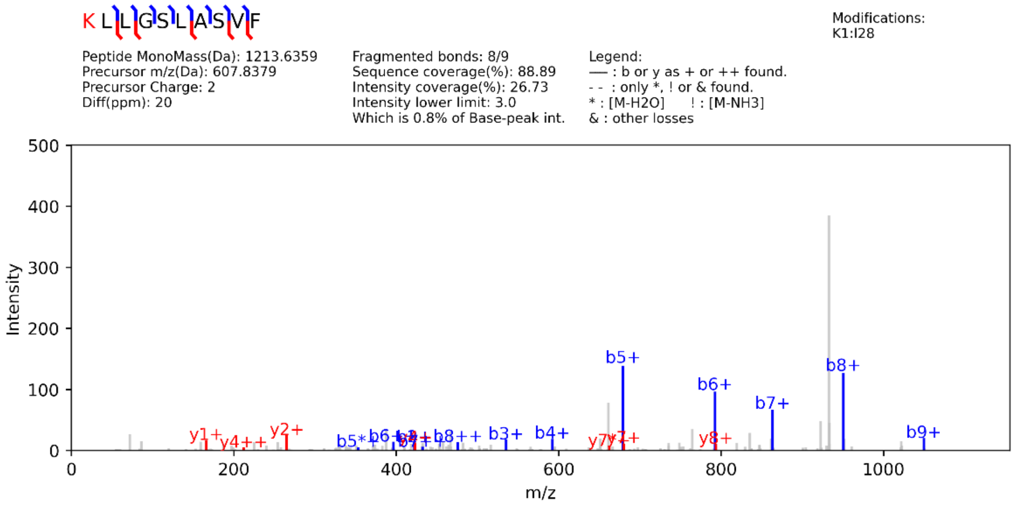

Sequence	Position start	Position end	Missed Cleavages	RT (min)
KLLGSLASVF	93	104	1	32.56

Figure S24. MS/MS spectra and analytical data of **32** modified **cHDAC4** enzyme peptides detected in the peptide mapping analysis using tryptic digestion. Labelled amino acid is **K93**.

1.3.9 Intact MS results of KRAS^{G12D}

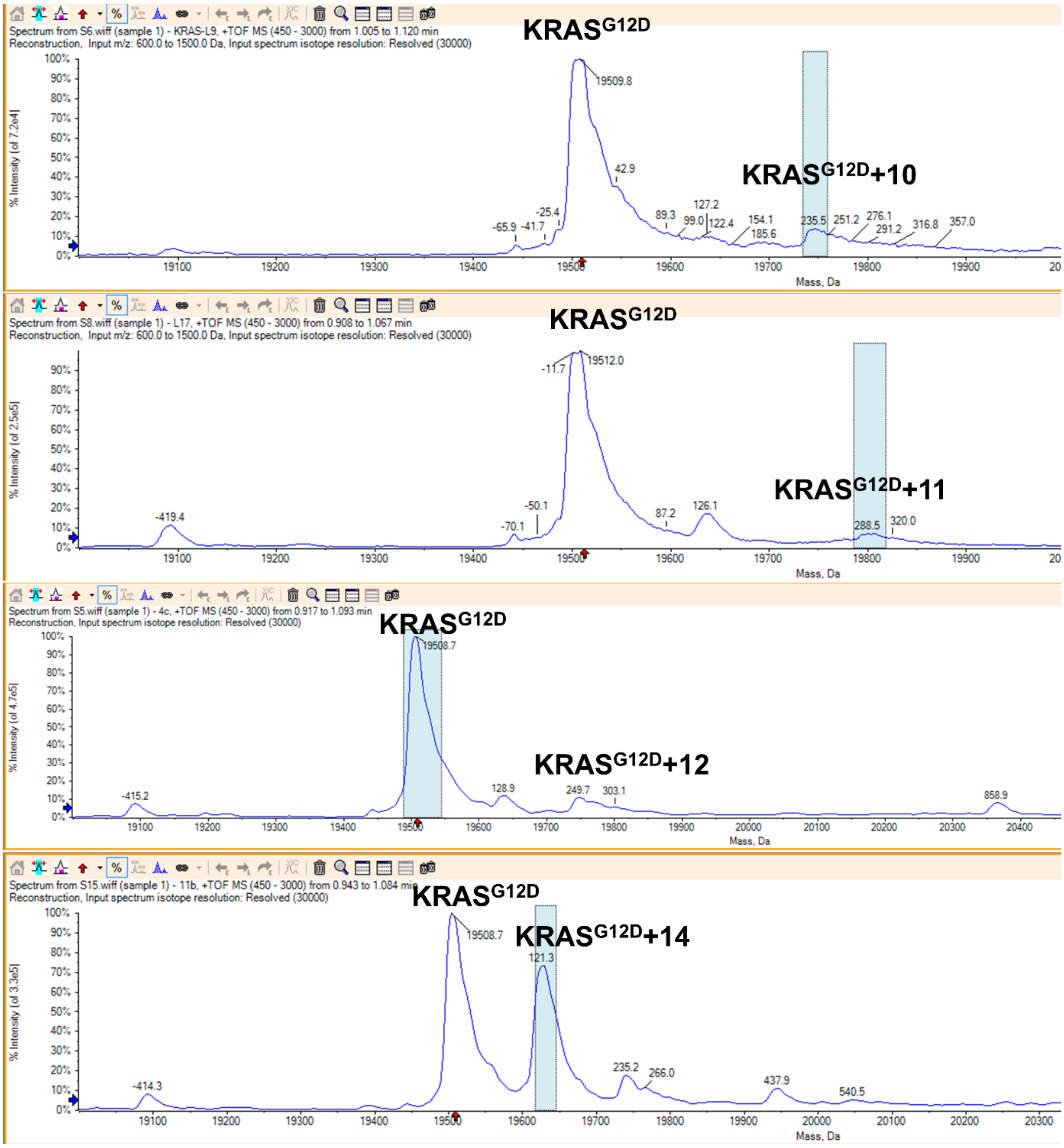
KRAS^G12D
Spectrum from S6.wiff (sample 1) - KRAS-L9, +TOF MS (450 - 3000) from 1.005 to 1.120 min
Reconstruction, Input m/z: 600.0 to 1500.0 Da, Input spectrum isotope resolution: Resolved (30000)
19509.8
KRAS^G12D+10
% Intensity (of 7.2e4)
Mass, Da
KRAS^G12D
Spectrum from S8.wiff (sample 1) - L17, +TOF MS (450 - 3000) from 0.908 to 1.067 min
Reconstruction, Input m/z: 600.0 to 1500.0 Da, Input spectrum isotope resolution: Resolved (30000)
19512.0
KRAS^G12D+11
% Intensity (of 2.5e5)
Mass, Da
KRAS^G12D
Spectrum from S5.wiff (sample 1) - 4c, +TOF MS (450 - 3000) from 0.917 to 1.093 min
Reconstruction, Input spectrum isotope resolution: Resolved (30000)
19508.7
KRAS^G12D+12
% Intensity (of 4.7e5)
Mass, Da
KRAS^G12D
Spectrum from S15.wiff (sample 1) - 11b, +TOF MS (450 - 3000) from 0.943 to 1.084 min
Reconstruction, Input spectrum isotope resolution: Resolved (30000)
19508.7
KRAS^G12D+14
121.3
% Intensity (of 3.3e5)
Mass, Da

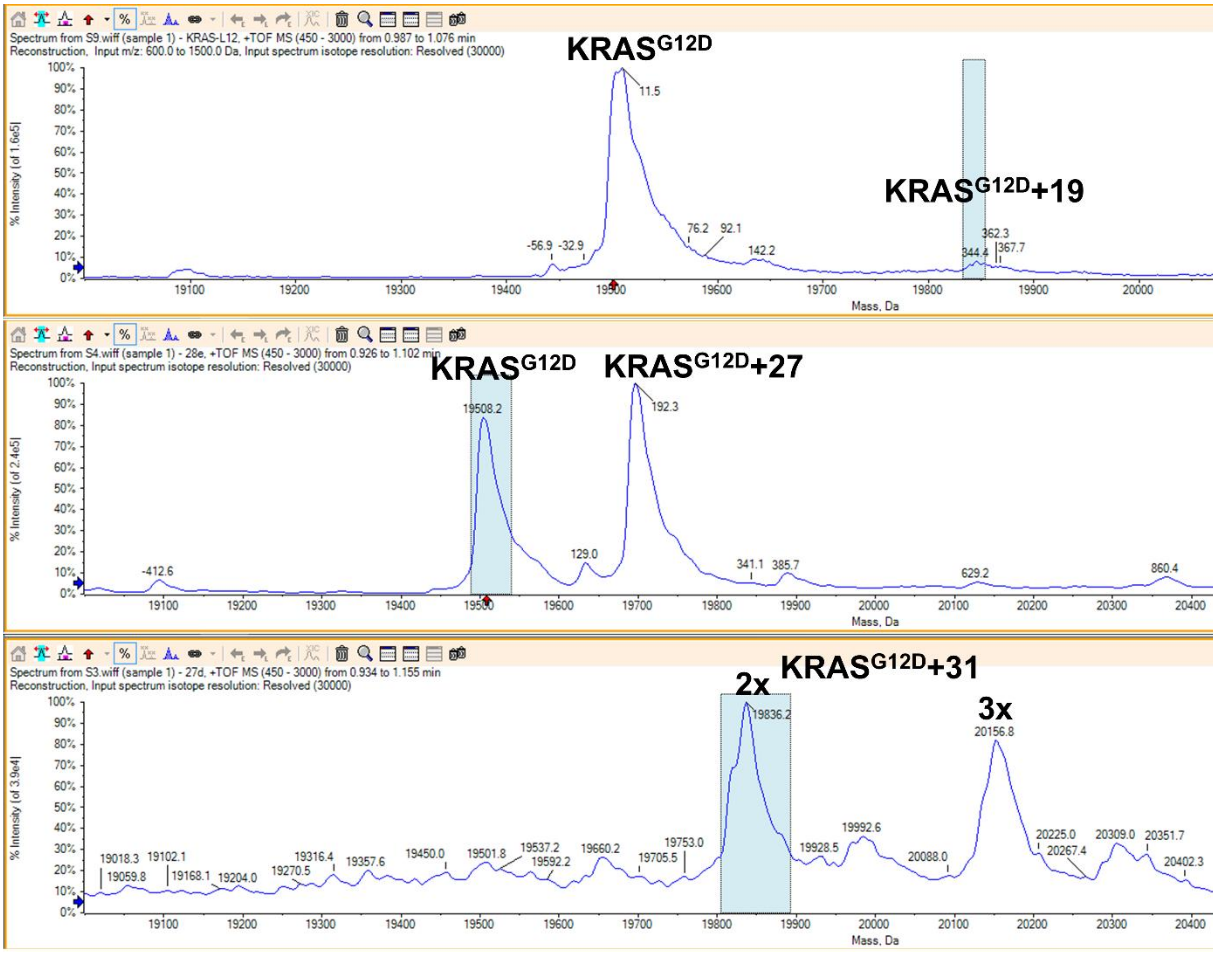

Figure S25. Deconvoluted mass spectrum of **KRAS^{G12D}** labelled by **10** (+240 Da), **11** (+296 Da), **12** (+243 Da), **14** (+119 Da), **19** (+340 Da) **27** (+192 Da) and **31** (+166 Da), respectively.

1.3.10 Digestion results of KRAS^{G12D}

GH-MTEY^{5}**K**LVVVGADGVGK

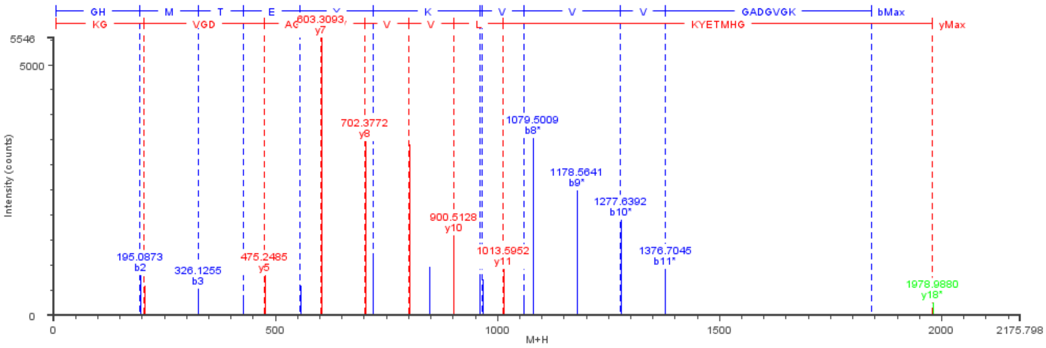

V^{104}**K**DSEDVPMVLVGNK

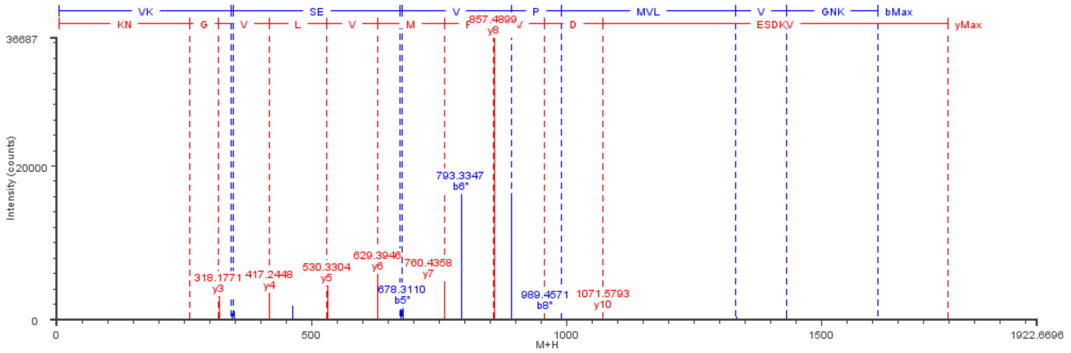

GIPFIETSA^{147}**K**TRQGVDDAFY

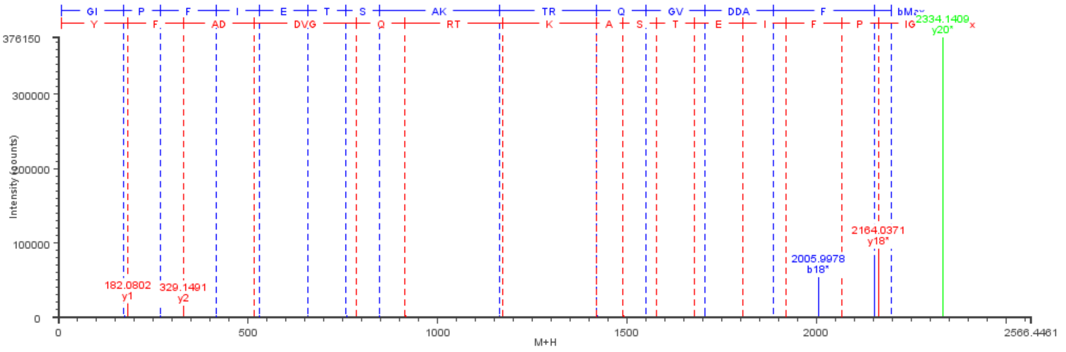

Sequence	Position start	Position end	Missed Cleavages	m/z	Charge	RT (min)
GH-MTEY**K**LVVVGADGVGK	1	16	1	660.3436	3	11.2
V**K**DSEDVPMVLVGNK	103	117	1	583.6381	3	11.4
GIPFIETSA**K**TRQGVDDAFY	138	157	2	778.7220	3	12.9

Figure S26. MS/MS spectra and analytical data of **14** modified **KRAS**$^{\mathbf{G12D}}$ peptides detected in the peptide mapping analysis using tryptic digestion. Labelled amino acids are **K5**, **K104** and **K147**, respectively.

GH-MTEY^{5}**K**LVVVGADGVGK

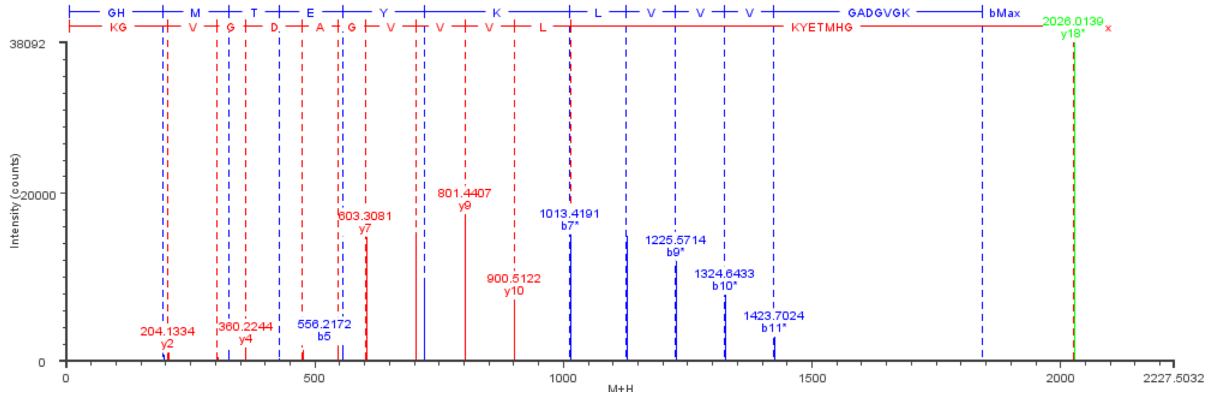

V^{104}**K**DSEDVPMVLVGNK

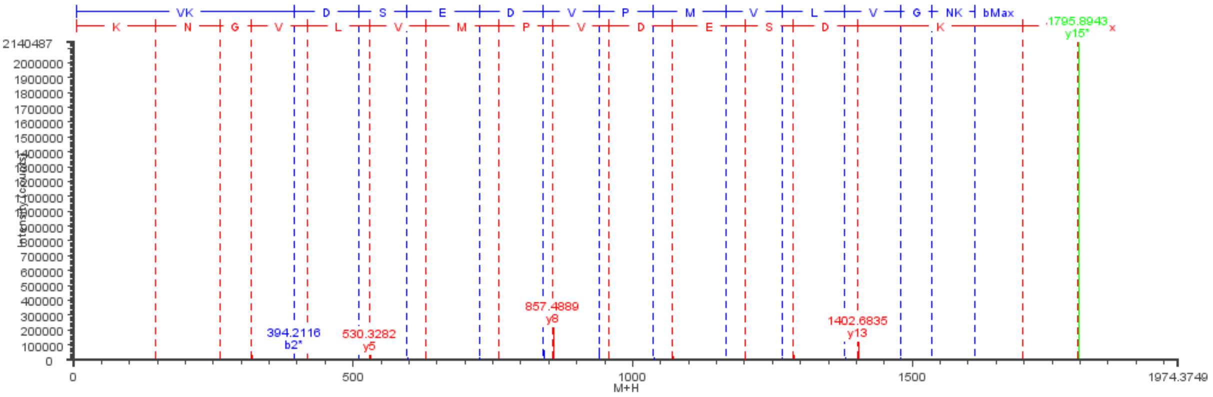

GIPFIETSA^{147}**K**TRQGVDDAFY

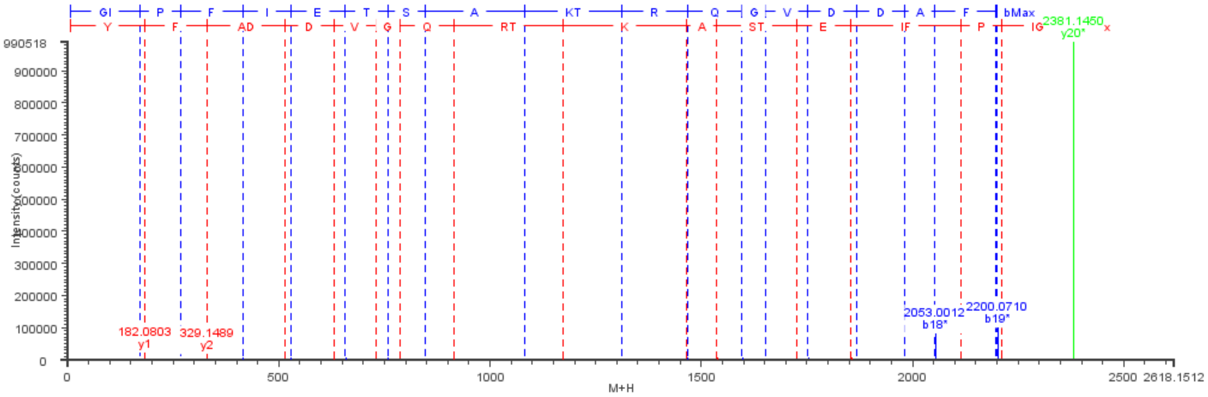

Sequence	Position start	Position end	Missed Cleavages	m/z	Charge	RT (min)
GH-MTEY**K**LVVVGADGVGK	1	16	1	676.0119	3	12.6
V**K**DSEDVPMVLVGNK	103	117	1	898.4539	2	12.9
GIPFIETSA**K**TRQGVDDAFY	138	157	2	794.3903	3	14.1

Figure S27. MS/MS spectra and analytical data of **31** modified **KRAS^{G12D}** peptides detected in the peptide mapping analysis using tryptic digestion. Labelled amino acids are **K5**, **K104** and **K147**, respectively.

1.3.11 Intact MS results of FBW7

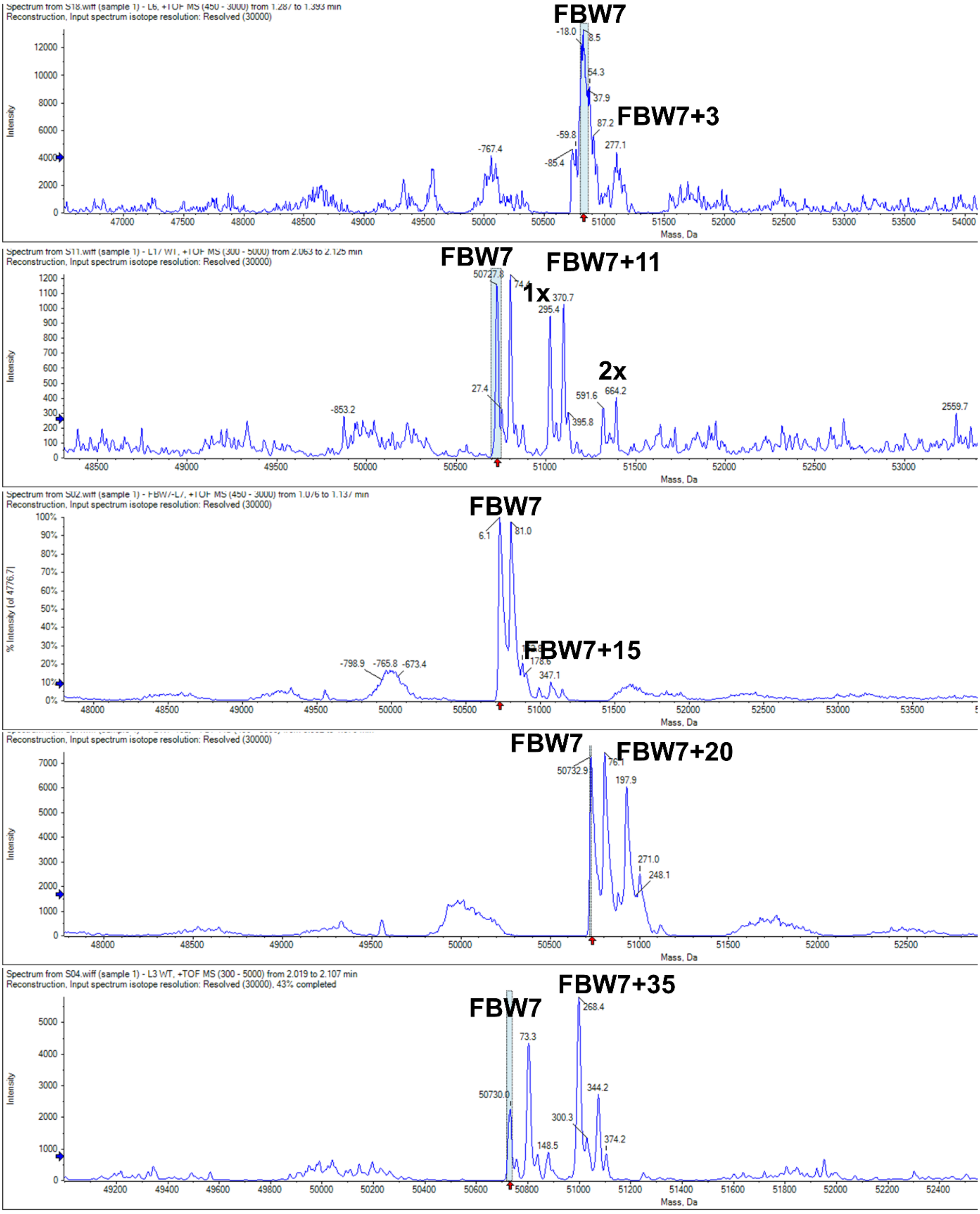

Figure S28. Deconvoluted mass spectrum of **FBW7** labeled by **3** (+271 Da), **11** (+296 Da), **15** (+345 Da), **20** (+198 Da) and **35** (+271 Da), respectively.

1.3.12 Digestion results of FBW7

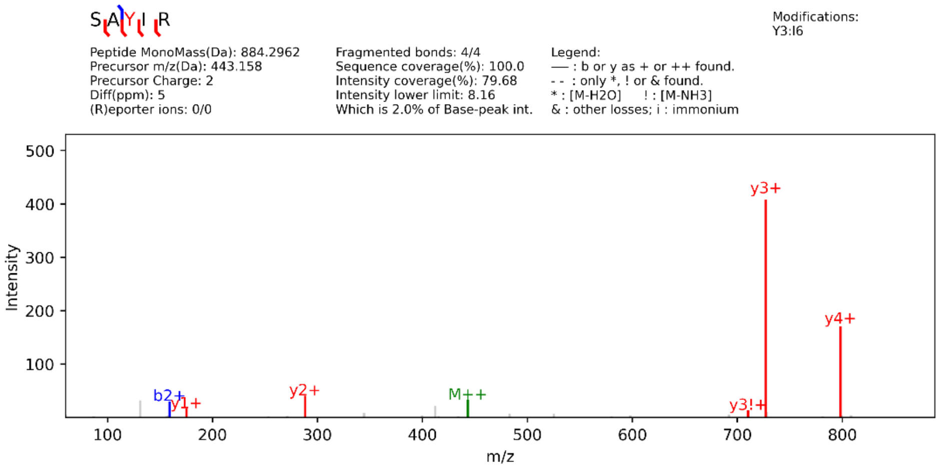

Sequence	Position start	Position end	Missed Cleavages	RT (min)
SAYIR	353	357	0	33.59

Figure S29. MS/MS spectra and analytical data of **3** modified **FBW7** peptides detected in the peptide mapping analysis using tryptic digestion. Labelled amino acid is **Y355**.

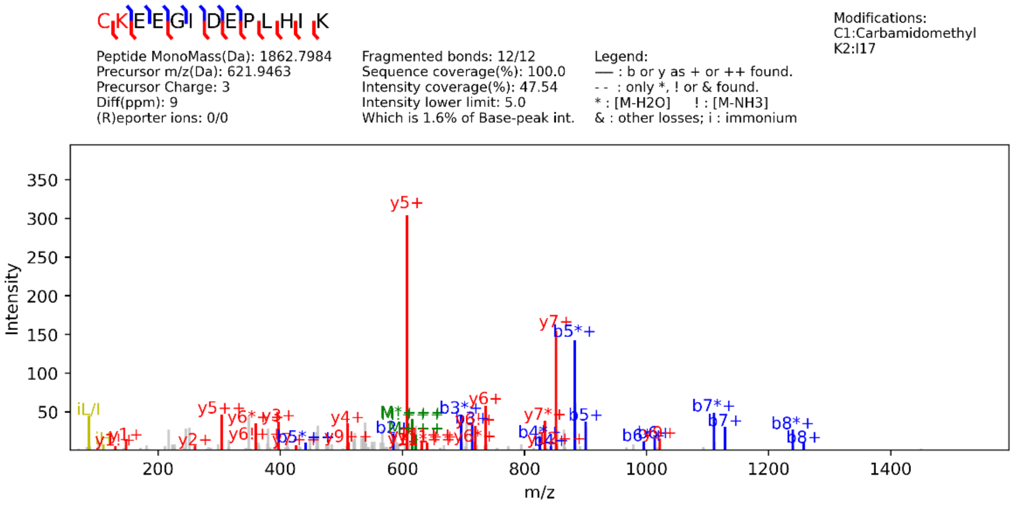

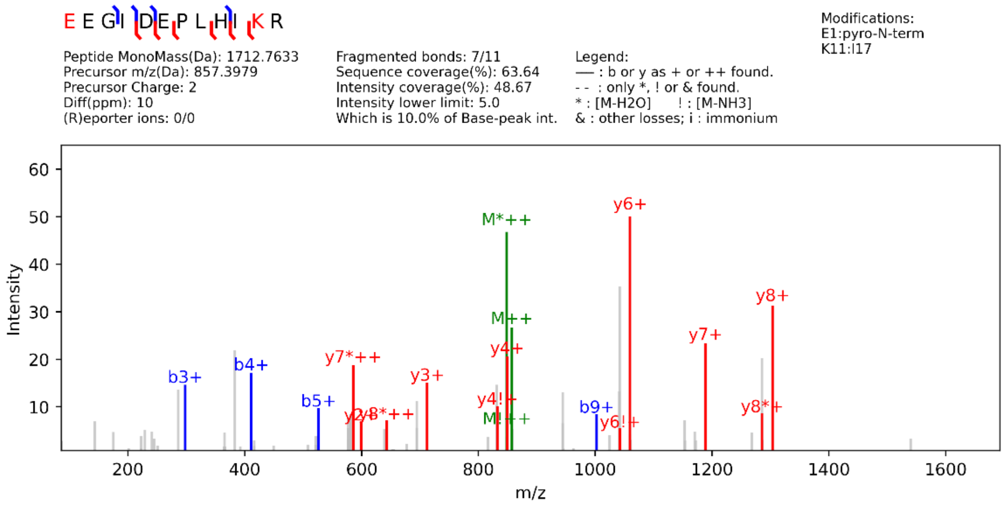
E E G I D E P L H I K R
Peptide MonoMass(Da): 1712.7633
Precursor m/z(Da): 857.3979
Precursor Charge: 2
Diff(ppm): 10
(R)eporter ions: 0/0
Fragmented bonds: 7/11
Sequence coverage(%): 63.64
Intensity coverage(%): 48.67
Intensity lower limit: 5.0
Which is 10.0% of Base-peak int.
Legend:
—— : b or y as + or ++ found.
- - : only *, ! or & found.
* : [M-H2O] ! : [M-NH3]
& : other losses; i : immonium
Modifications:
E1:pyro-N-term
K11:I17
Intensity
m/z

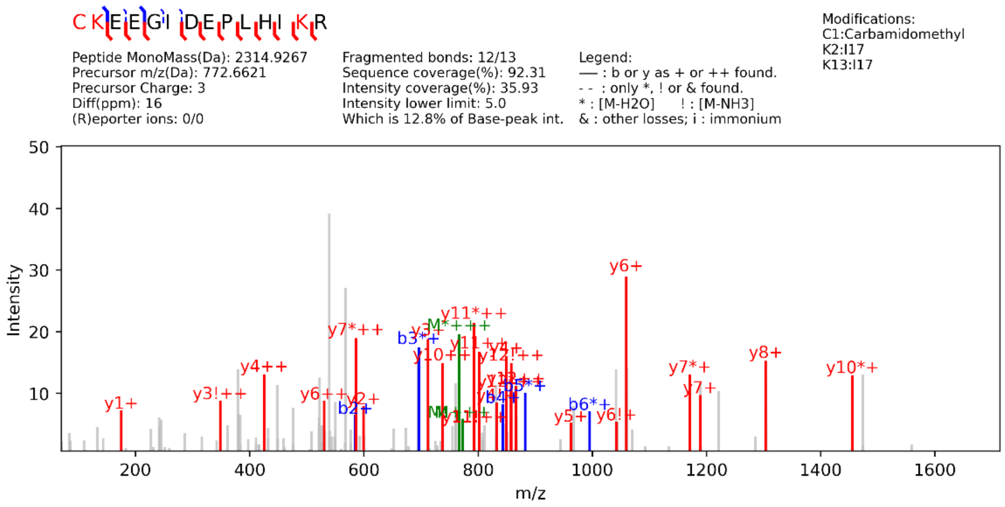
C K E E G I D E P L H I K R
Peptide MonoMass(Da): 2314.9267
Precursor m/z(Da): 772.6621
Precursor Charge: 3
Diff(ppm): 16
(R)eporter ions: 0/0
Fragmented bonds: 12/13
Sequence coverage(%): 92.31
Intensity coverage(%): 35.93
Intensity lower limit: 5.0
Which is 12.8% of Base-peak int.
Legend:
—— : b or y as + or ++ found.
- - : only *, ! or & found.
* : [M-H2O] ! : [M-NH3]
& : other losses; i : immonium
Modifications:
C1:Carbamidomethyl
K2:I17
K13:I17
Intensity
m/z

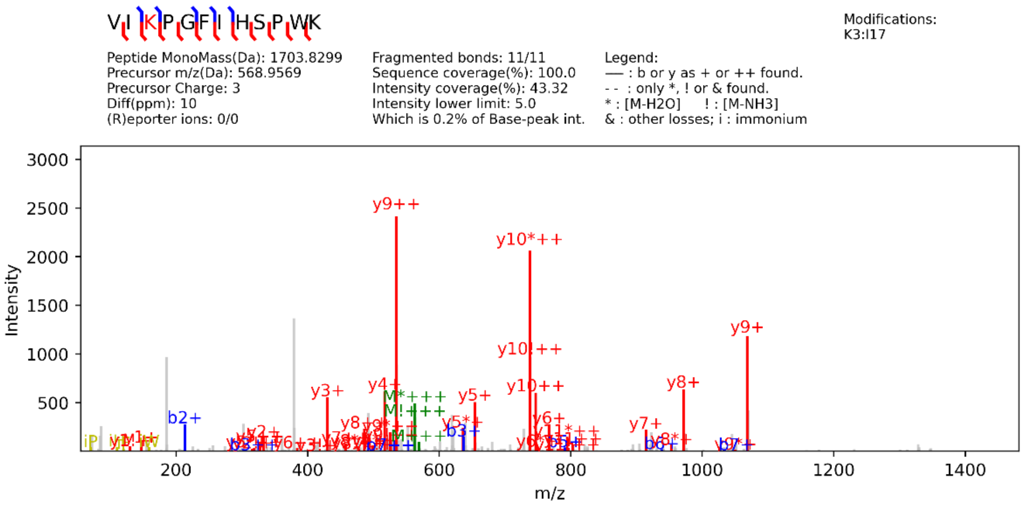
V I K P G F I H S P W K
Peptide MonoMass(Da): 1703.8299
Precursor m/z(Da): 568.9569
Precursor Charge: 3
Diff(ppm): 10
(R)eporter ions: 0/0
Fragmented bonds: 11/11
Sequence coverage(%): 100.0
Intensity coverage(%): 43.32
Intensity lower limit: 5.0
Which is 0.2% of Base-peak int.
Legend:
—— : b or y as + or ++ found.
- - : only *, ! or & found.
* : [M-H2O] ! : [M-NH3]
& : other losses; i : immonium
Modifications:
K3:I17
Intensity
m/z

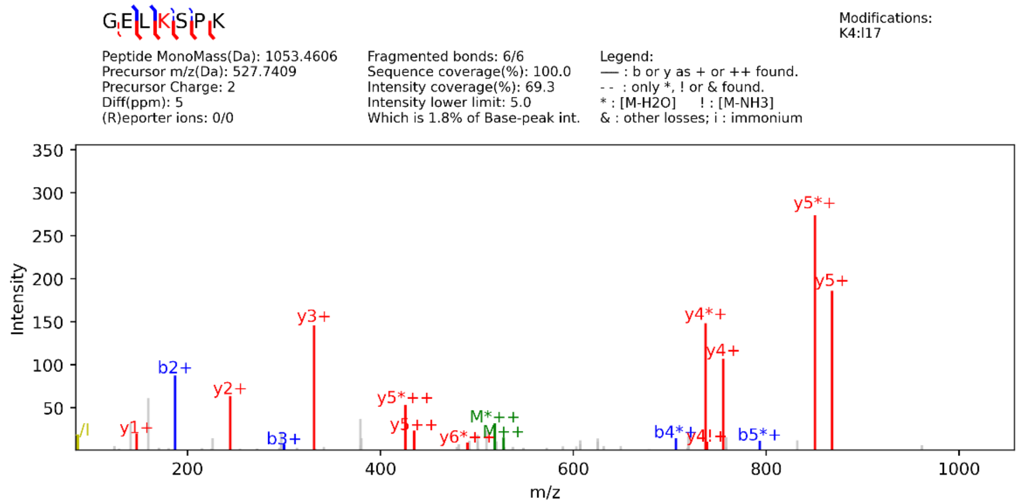
G E L K S P K
Modifications:
K4:I17
Peptide MonoMass(Da): 1053.4606
Precursor m/z(Da): 527.7409
Precursor Charge: 2
Diff(ppm): 5
(R)eporter ions: 0/0
Fragmented bonds: 6/6
Sequence coverage(%): 100.0
Intensity coverage(%): 69.3
Intensity lower limit: 5.0
Which is 1.8% of Base-peak int.
Legend:
── : b or y as + or ++ found.
- - : only *, ! or & found.
* : [M-H2O] ! : [M-NH3]
& : other losses; i : immonium
Intensity
m/z
y5*+
y5+
y3+
y4*+
y4+
b2+
y2+
y5*++
M*++
y1+
b3+
b4*+
b5*+

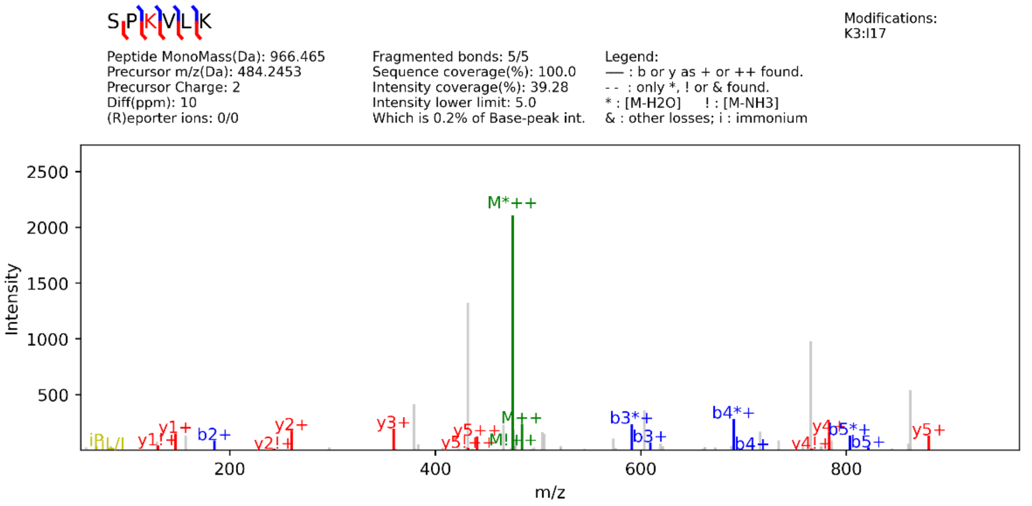
S P K V L K
Modifications:
K3:I17
Peptide MonoMass(Da): 966.465
Precursor m/z(Da): 484.2453
Precursor Charge: 2
Diff(ppm): 10
(R)eporter ions: 0/0
Fragmented bonds: 5/5
Sequence coverage(%): 100.0
Intensity coverage(%): 39.28
Intensity lower limit: 5.0
Which is 0.2% of Base-peak int.
Legend:
── : b or y as + or ++ found.
- - : only *, ! or & found.
* : [M-H2O] ! : [M-NH3]
& : other losses; i : immonium
Intensity
m/z
M*++
b3*+
b4*+
y1+
b2+
y2+
y3+
b3+
b4+
b5+
y5+

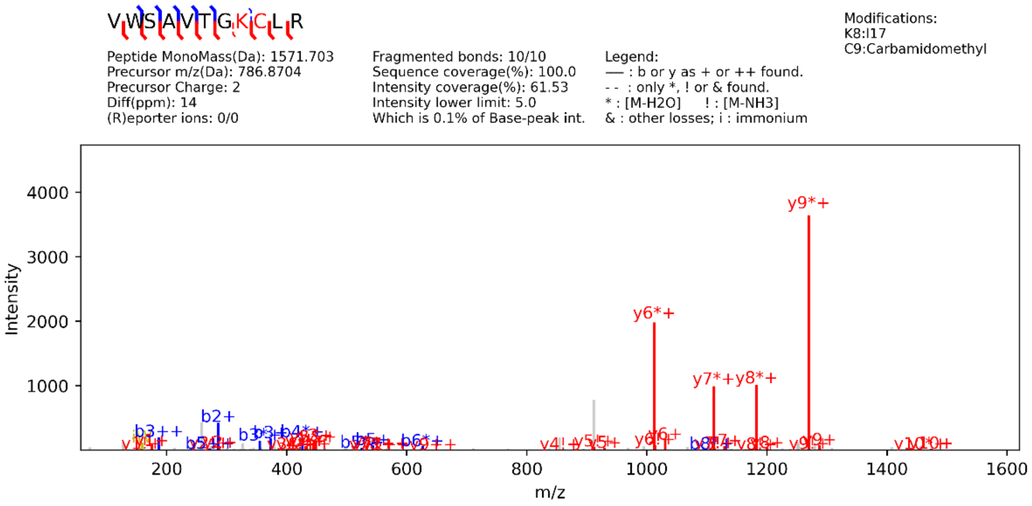
V W S A V T G K C L R
Modifications:
K8:I17
C9:Carbamidomethyl
Peptide MonoMass(Da): 1571.703
Precursor m/z(Da): 786.8704
Precursor Charge: 2
Diff(ppm): 14
(R)eporter ions: 0/0
Fragmented bonds: 10/10
Sequence coverage(%): 100.0
Intensity coverage(%): 61.53
Intensity lower limit: 5.0
Which is 0.1% of Base-peak int.
Legend:
── : b or y as + or ++ found.
- - : only *, ! or & found.
* : [M-H2O] ! : [M-NH3]
& : other losses; i : immonium
Intensity
m/z
y9*+
y6*+
y7*+
y8*+
b2+

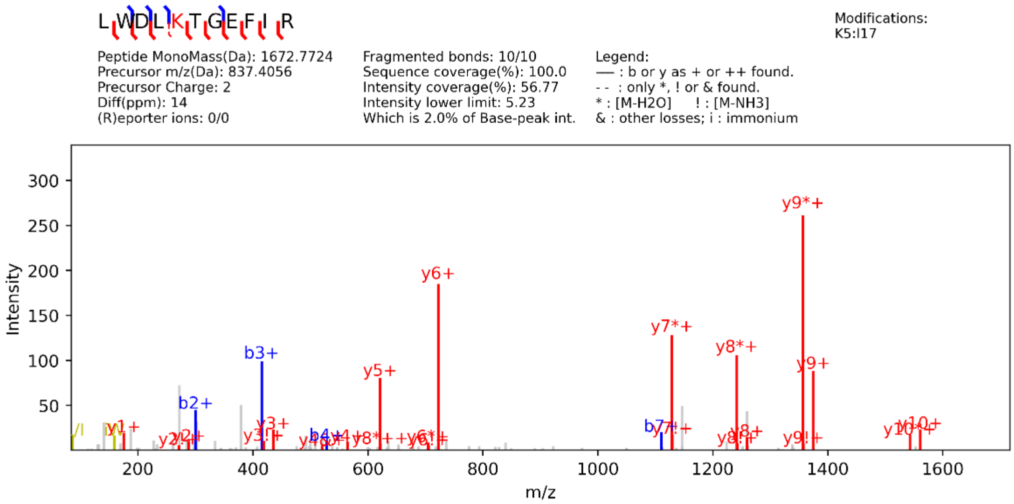

Sequence	Position start	Position end	Missed Cleavages	RT (min)
C**K**EEGIDEPLHIK	325	337	1	31.83
EEGIDEPLHI**K**R	327	338	1	33.77
C**K**EEGIDEPLHI**K**R	325	338	2	36.51
VI**K**PGFIHSPWK	341	352	1	34.37
GEL**K**SPK	368	374	1	31.47
SP**K**VLK	372	377	1	32.98
VWSAVTG**K**CLR	405	415	1	34.56
LWDL**K**TGEFIR	648	658	1	37.39

Figure S30. MS/MS spectra and analytical data of **11** modified **FBW7** peptides detected in the peptide mapping analysis using tryptic digestion. Labelled amino acids are **K326**, **K337**, **K343**, **K371**, **K374**, **K412** and **K652**, respectively.

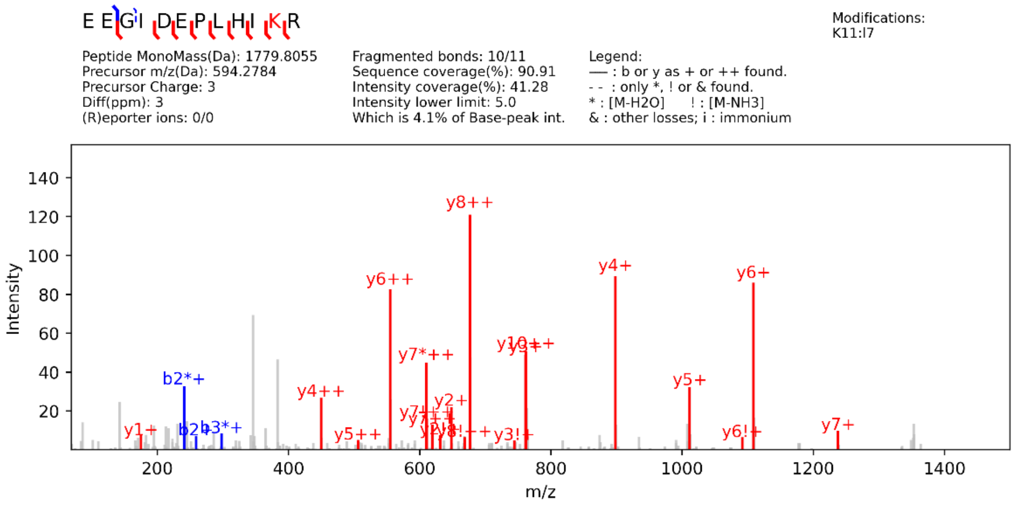

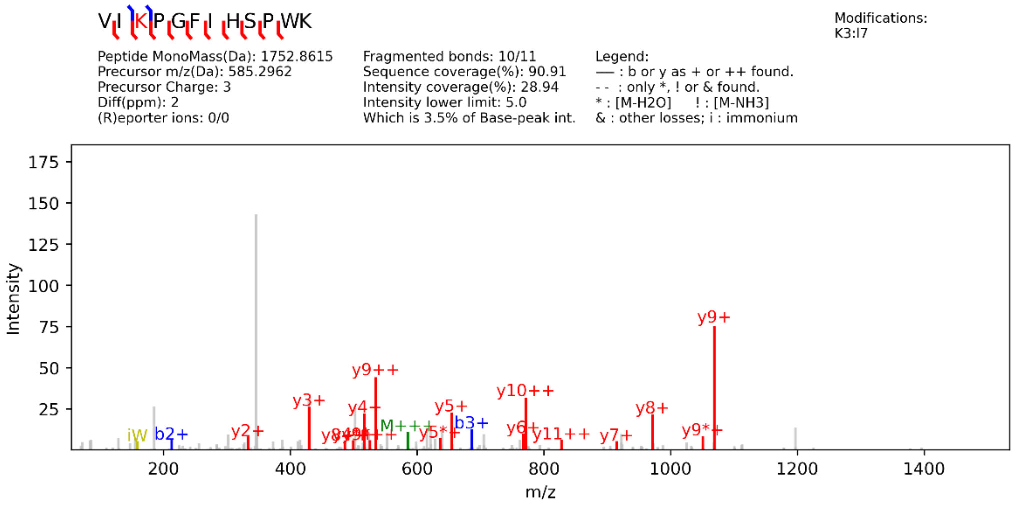

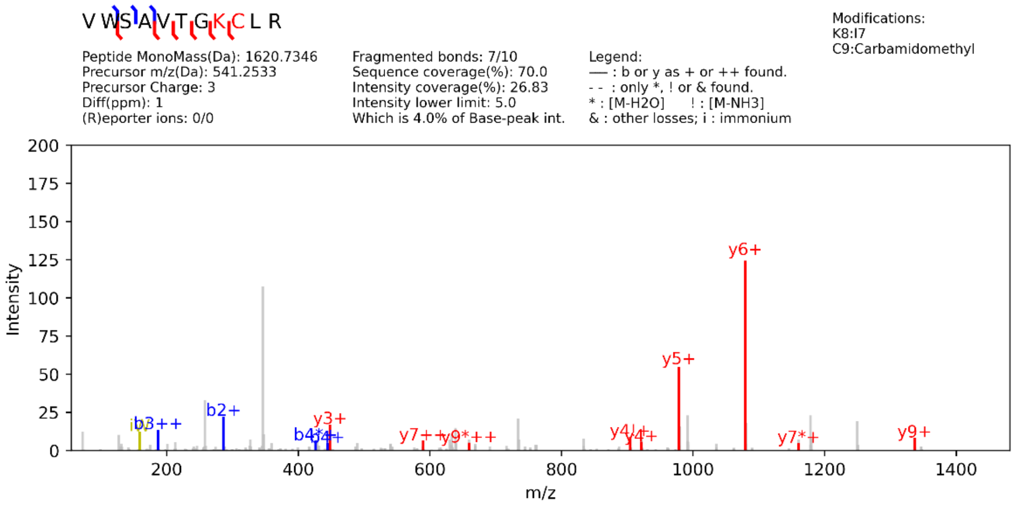

Sequence	Position start	Position end	Missed Cleavages	RT (min)

EEGIDEPLHI**K**R	327	338	1	36.36
VI**K**PGFIHSPWK	341	352	1	37.90
VWSAVTG**K**CLR	405	415	1	38.40

Figure S31. MS/MS spectra and analytical data of **15** modified **FBW7** peptides detected in the peptide mapping analysis using tryptic digestion. Labelled amino acids are **K337**, **K343** and **K412**, respectively.

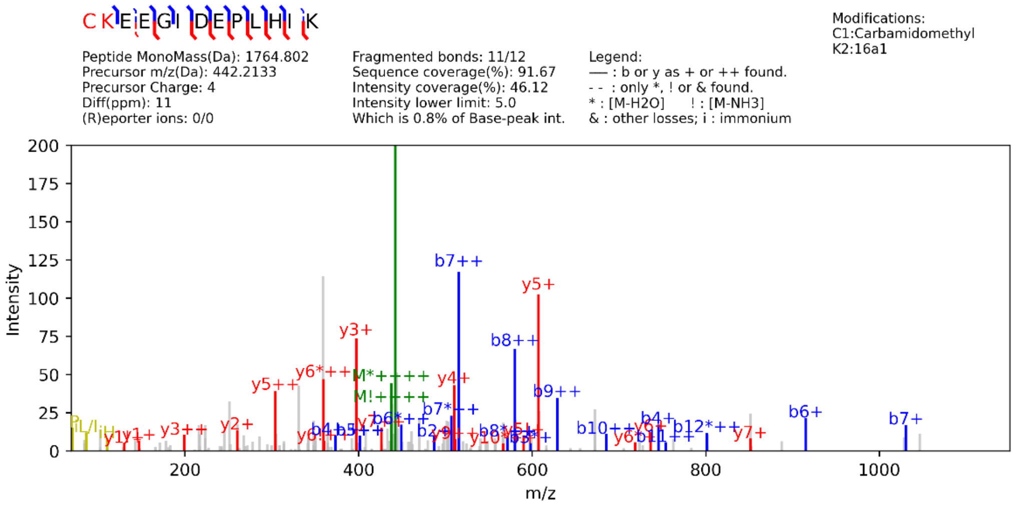

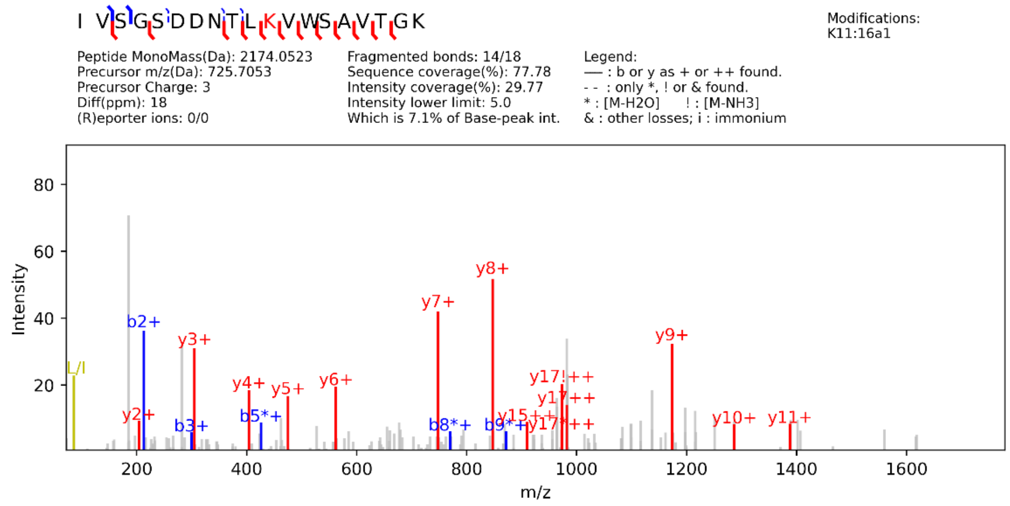

Sequence	Position start	Position end	Missed Cleavages	RT (min)
C**K**EEGIDEPLHIK	325	337	1	26.32
IVSGSDDNTL**K**VWSAVTGK	394	412	1	30.88

Figure S32. MS/MS spectra and analytical data of **20** modified **FBW7** peptides detected in the peptide mapping analysis using tryptic digestion. Labelled amino acids are **K326** and **K404**, respectively.

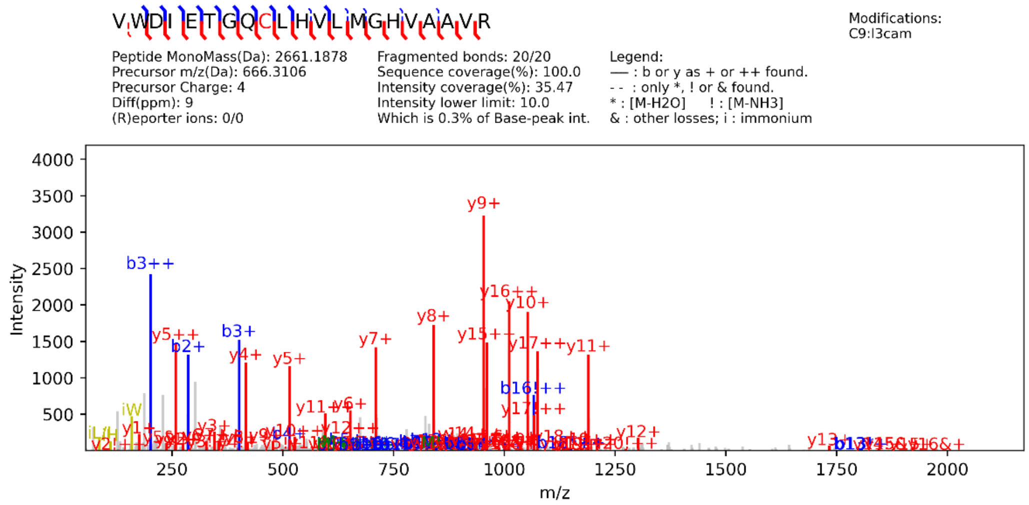

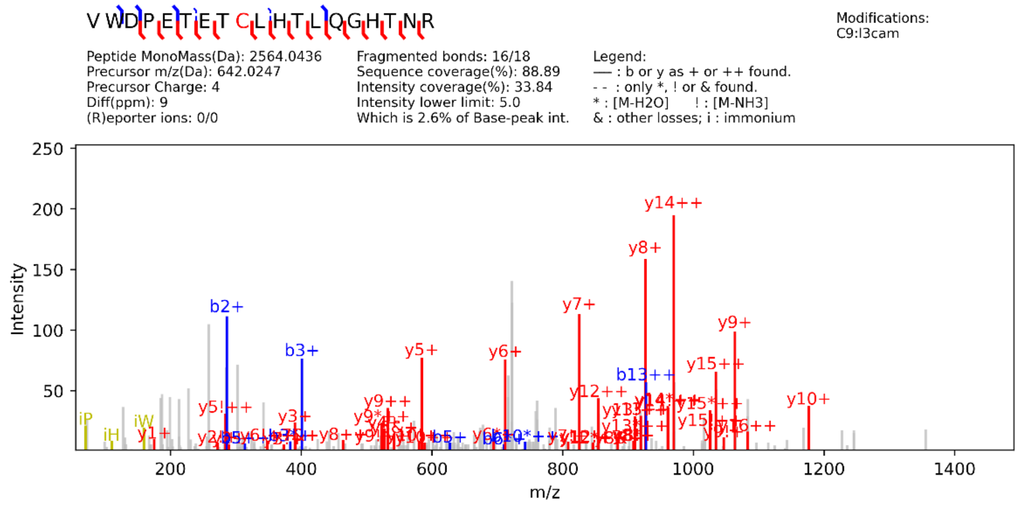

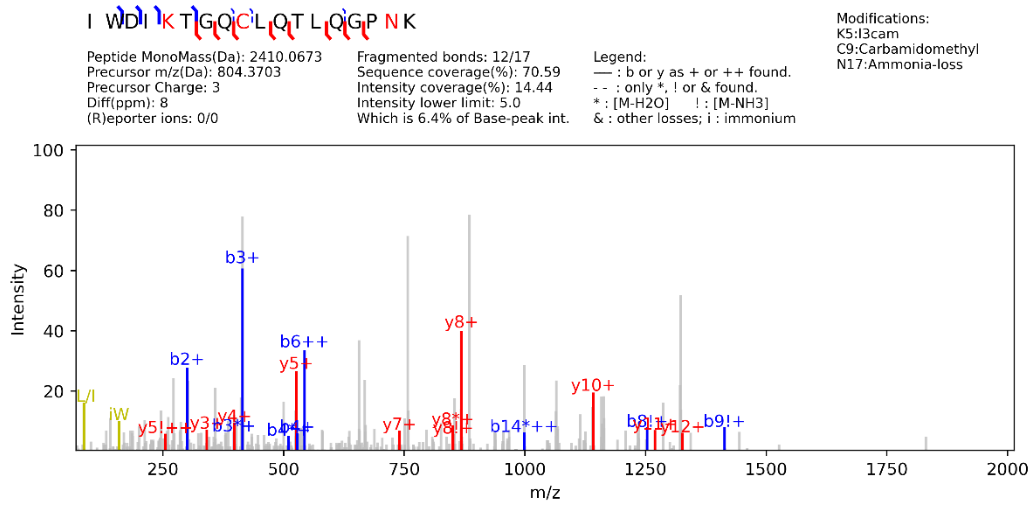

Sequence	Position start	Position end	Missed Cleavages	RT (min)
VWDIETGQ**C**LHVLMGHVAAVR	485	505	0	34.09
VWDPETET**C**LHTLQGHTNR	525	543	0	30.63
IWDI**K**TGQCLQTLQGPNK	605	622	1	37.43

Figure S33. MS/MS spectra and analytical data of **35** modified **FBW7** peptides detected in the peptide mapping analysis using tryptic digestion. Labelled amino acids are **C493**, **C533** and **K609**, respectively.

1.4 Bibliography

1. Tung, C. L., Wong, C. T. T., Fung, E. Y. M. & Li, X. Traceless and Chemoselective Amine Bioconjugation via Phthalimidine Formation in Native Protein Modification. *Org. Lett.* **18**, 2600–2603 (2016).

2. Chen, P. *et al.* 2-Ethynylbenzaldehyde-Based, Lysine-Targeting Irreversible Covalent Inhibitors for Protein Kinases and Nonkinases. *J. Am. Chem. Soc.* **145**, 3844–3849 (2023).

3. Lefèbre, J. *et al.* Covalent Activation of the C-type Lectin DC-SIGN. *Angewandte Chemie - Int. Ed.* **2025**, e20594 (2025).

4. Belo, Y. *et al.* Unexpected implications of STAT3 acetylation revealed by genetic encoding of acetyl-lysine. *Biochimica et Biophysica Acta (BBA) - General Subjects* **1863**, 1343–1350 (2019).

5. Canon, J. *et al.* The clinical KRAS(G12C) inhibitor AMG 510 drives anti-tumour immunity. *Nature* **575**, 217–223 (2019).

6. Hao, B., Oehlmann, S., Sowa, M. E., Harper, J. W. & Pavletich, N. P. Structure of a Fbw7-Skp1-Cyclin E Complex: Multisite-Phosphorylated Substrate Recognition by SCF Ubiquitin Ligases. *Mol. Cell* **26**, 131–143 (2007).

7. Proj, M., Bozovičar, K., Hrast, M., Frlan, R. & Gobec, S. DNA-encoded library screening on two validated enzymes of the peptidoglycan biosynthetic pathway. *Bioorg. Med. Chem. Lett.* **73**, 128915 (2022).

8. Batson, S. *et al.* Inhibition of D-Ala:D-Ala ligase through a phosphorylated form of the antibiotic D-cycloserine. *Nat. Commun.* **8**, 1939 (2017).

9. Wéber, E. *et al.* Target-Templated Construction of Functional Proteomimetics Using Photo-Foldamer Libraries. *Angew. Chem. Int. Ed.* **64**, e202410435 (2025).

10. Martínez-Cartró, M. *et al.* Discovering Uncharted Binding Pockets on E3 Ligases Leads to the Identification of FBW7 Allosteric Modulators. *Advanced Science* **12**, e06068 (2025).

11. Madhavi Sastry, G., Adzhigirey, M., Day, T., Annabhimoju, R. & Sherman, W. Protein and ligand preparation: parameters, protocols, and influence on virtual screening enrichments. *J. Comput. Aided. Mol. Des.* **27**, 221–234 (2013).

12. Zhu, K. *et al.* Docking Covalent Inhibitors: A Parameter Free Approach To Pose Prediction and Scoring. *J. Chem. Inf. Model.* **54**, 1932–1940 (2014).

13. Kozakov, D. *et al.* The FTMap family of web servers for determining and characterizing ligand-binding hot spots of proteins. *Nat. Protoc.* **10**, 733–755 (2015).

14. Brenke, R. *et al.* Fragment-based identification of druggable 'hot spots' of proteins using Fourier domain correlation techniques. *Bioinformatics* **25**, 621–627 (2009).

15. Halgren, T. A. Identifying and Characterizing Binding Sites and Assessing Druggability. *J. Chem. Inf. Model.* **49**, 377–389 (2009).

16. Halgren, T. New method for fast and accurate binding-site identification and analysis. *Chem. Biol. Drug Des.* **69**, 146–148 (2007).

17. Bottomley, M. J. *et al.* Structural and Functional Analysis of the Human HDAC4 Catalytic Domain Reveals a Regulatory Structural Zinc-binding Domain. *Journal of Biological Chemistry* **283**, 26694–26704 (2008).

18. Friesner, R. A. *et al.* Glide: A New Approach for Rapid, Accurate Docking and Scoring. 1. Method and Assessment of Docking Accuracy. *J. Med. Chem.* **47**, 1739–1749 (2004).

19. Halgren, T. A. *et al.* Glide: A New Approach for Rapid, Accurate Docking and Scoring. 2. Enrichment Factors in Database Screening. *J. Med. Chem.* **47**, 1750–1759 (2004).
